# Role of PER-WDR5 interactions in the mammalian circadian clock

**DOI:** 10.1038/s41467-026-77583-0

**Published:** 2026-09-08

**Authors:** Arne Börgel, Florian Hof, Mareen Welzel, Audrey Rawleigh, Marcel Wedemann, Rafaela Mororo Cavalcanti Keller, Vanessa Maissl, Kyra Ricci, Sandra Schick, Helen L. May-Simera, Steven Brown, Achim Kramer, Katja Luck, Jürgen A. Ripperger, Eva Wolf

**Affiliations:** 1 Institute of Molecular Physiology (IMP), Johannes Gutenberg-University Mainz, Mainz, Germany; 2 Integrative Systems Biology Group, Institute of Molecular Biology (IMB), Mainz, Germany; 3 Chronobiology and Sleep Research Group, Institute of Pharmacology and Toxicology, University of Zürich, Zürich, Switzerland; 4 Laboratory of Chronobiology, Charité-Universitätsmedizin Berlin, Berlin, Germany; 5 Chromatin Regulation Group, Institute of Molecular Biology (IMB), Mainz, Germany; 6 Department of Biology, University of Fribourg, Fribourg, Switzerland; 7 Present Address: Technische Hochschule Bingen, Bingen, Germany

**Keywords:** Circadian rhythm signalling peptides and proteins, X-ray crystallography

## Abstract

In the mammalian circadian clock, the transcription factor CLOCK/BMAL1 cycles between an active state recruiting co-activators like MLL1 and repressed states associated with PER1/2 and CRY1/2 clock proteins. The MLL1 complex component WDR5 was also found in a repressive PER complex. Here we show that WDR5 directly binds to the CRY binding domain (CBD) regions of PER1 and PER2. PER2 binds WDR5 via a WBM motif within the PER2/CRY interface, imposing competition between PER2/WDR5-, PER2/CRY- and RbBP5/WDR5 complexes. PER1 binds WDR5 predominantly via a WIN motif outside the CBD, enabling formation of trimeric PER1_WIN_/WDR5/RbBP5_WBM_ - and PER1/WDR5/CRY complexes. WDR5 WIN site inhibitors weaken PER1/WDR5 interactions and impact cellular *Bmal1-* and *Per2* expression dependent on the PER1 WIN motif, likely modulating the primary BMAL1/CLOCK/PER/CRY- and secondary BMAL1/REV-ERB/ROR circadian feedback loop. Together, our studies revealed a role of WDR5 as a direct PER interaction partner with critical differences between PER1 and PER2.

## Abstract (web summary)

Here the authors reveal that the mammalian clock proteins PERIOD 1 and 2 (PER1, PER2) interact with WDR5 using different WDR5 binding sites and that disrupting the PER1/WDR5 complex with WDR5 WIN site inhibitors affects circadian clock function.

## Introduction

In mammals, circadian clocks regulate many physiological processes such as metabolism, sleep-wake cycles and immune response. Circadian clocks are operated by cell-autonomous transcriptional and translational feedback loops^1^. The transcription factors CLOCK and BMAL1 heterodimerize to activate genes of the clock proteins Period1/2 (PER1/2) and Cryptochrome1/2 (CRY1/2), which feedback repress CLOCK/BMAL1. In a second feedback loop, the circadian expression of *Bmal1* is ensured by two nuclear receptors, RORα/β and REV-ERBα/β, where RORα/β activates- and REV-ERBα/β represses *Bmal1*^2,3^. During the circadian cycle, CLOCK/BMAL1 transitions between transcriptionally active-, early repressed-, and late repressed-states^4^. In the active state, CLOCK/BMAL1 recruits co-activators such as the histone methyltransferase Mixed-lineage leukaemia 1 (MLL1)^5^. In the early repressed state, CRY and PER repress CLOCK/BMAL1 within a large multi-subunit complex, that contains casein kinase 1δ/ε (CK1δ/ε) as well as RNA binding- and chromatin modifying proteins such as histone deacetylases, repressive methyltransferases and the WD repeat-containing protein 5 (WDR5)^2,6–11^. In the late repressed state, only CRY1 remains bound to CLOCK/BMAL1^2,4^.

**Fig. 1:**
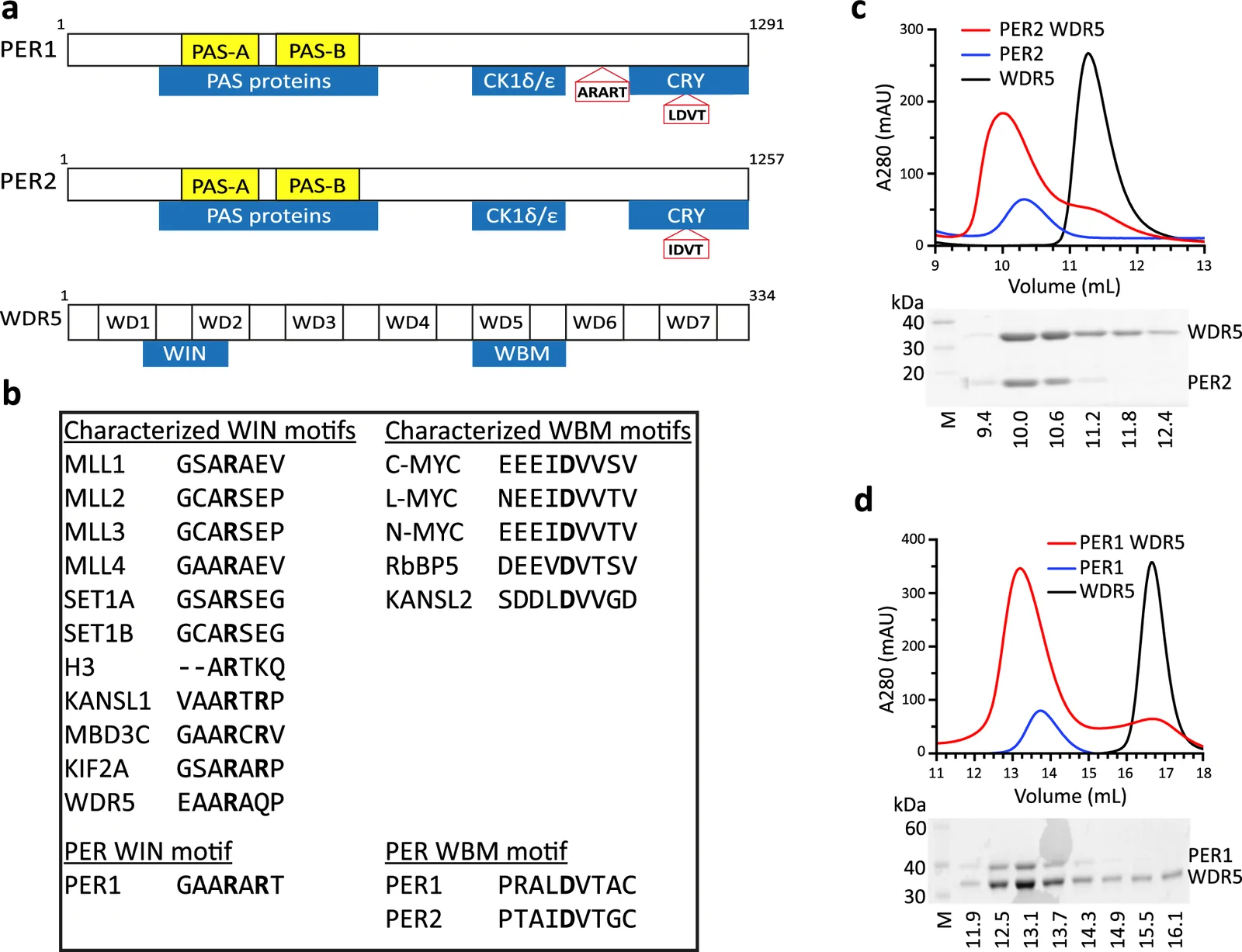
WDR5 directly binds to the CRY-binding regions of PER1 and PER2. **a** Domains (yellow) and interaction partners (blue) of PER1, PER2 and WDR5. Red boxes indicate potential WDR5 binding WIN (ARART) - and WBM (L/IDVT) motifs in PER1/2. PER1/2 have two PAS (PER-ARNT-SIM) domains (PAS-A, PAS-B) to dimerize and interact with other proteins. The middle and C-terminal regions of PER1/2 interact with CK1δ/ε and CRY1/2, respectively. WDR5 consists of 7 WD repeats (WD1-7) and has two binding sites to interact with proteins containing a WIN- or WBM motif. **b** Alignment of WDR5 binding motifs. WIN motifs have a conserved central arginine (bold), WBM motifs consist of a conserved aspartate (bold) flanked by aliphatic amino acids. **c** Analytical SEC (S75 10/300) of WDR5 22-334 and PER2 1132-1252. Top: chromatograms of WDR5 22-334 (black), PER2 1132-1252 (blue), and PER2/WDR5 complex (red). Bottom: SDS-PAGE of PER2/WDR5 complex SEC. M: molecular weight marker. The PER2/WDR5 complex elutes earlier (10 mL) than free WDR5 (>11 mL). SEC results are representative of *n* = 3 independent experiments. **d** Analytical SEC (S200 10/300) of WDR5 22-334 and PER1 1013-1291. Top: chromatograms of WDR5 22-334 (black), PER1 1013-1291 (blue) and PER1/WDR5 complex (red). Bottom: SDS-PAGE of PER1/WDR5 complex SEC. The PER1/WDR5 complex elutes earlier (~13 mL) than free WDR5 (>16 mL). SEC results are representative of *n* = 3 independent experiments. Source data for **c**, **d** are provided as a Source data file.

PER1 and PER2 are thought to have partially redundant roles but also have distinct impacts on the circadian clock. While disruption of PER1 leads to a shorter circadian period in mice^12,13^ and a lower circadian amplitude and shortened period in cultured cells^14^, PER2 inactivation results in a loss of circadian rhythm in both systems^14,15^. Furthermore, PER1 is present on circadian promotors several hours before PER2^4,7,9^ and associated with different chromatin modifying complexes than PER2^9^. Both, PER1 and PER2 can dimerize or interact with other proteins via their PAS (PER-ARNT-SIM) domains^16,17^. Additionally, CK1δ/ε binds to the central PER region^18^, while CRY1/2 bind to the C-terminal region of PER1 and PER2 (Fig. 1a)^19–21^.

WDR5 is a scaffold protein in various multiprotein complexes, such as the SET1/MLL histone H3K4 methyltransferase complexes, the non-specific lethal (NLS) histone acetyltransferase complex or the nucleosome remodeling and deacetylase (NuRD) complex^22^. It possesses two binding pockets that interact with WDR5 interacting motifs (=WIN motifs, A/S/C-A-**R-**A/S/C/T) and with WDR5 binding motifs (=WBM motifs, -L/I/V-**D**-V-V/T) provided by its varying interaction partners (Fig. 1a, b)^22^. The active MLL1 complex consists of five proteins, namely the catalytically active Histone-lysine N-methyltransferase 2 A (KMT2A, MLL1), WDR5, Retinoblastoma binding protein 5 (RbBP5), Absent small homeotic-2-like protein (ASH2L) and DPY30, and its presence enhances histone methylation activity by several hundred fold^23^. Within this complex WDR5 binds directly to the WIN motif (GSARAEV) of MLL1 and to the WBM motif (EEVDVT) of RbBP5 (Fig. 1b)^24–28^. In the circadian clock, WDR5 was identified as part of the MLL1 complex, which is recruited to CLOCK/BMAL1 in a circadian manner to co-activate circadian genes by di- and tri-methylation of histone H3 lysine K4 (H3K4)^5,29^, as well as in repressive PER containing complexes^7^. Furthermore, WDR5 was reported to interact with PER1 and PER2 in cell-based Co-IP studies^7^. Depletion of WDR5 abolishes circadian methylation rhythms of both H3K4 and H3K9, suggesting a role of WDR5 in transcriptional activation (H3K4 methylation) and repression (H3K9 methylation)^7^. Hence, PER-WDR5 interactions could affect CLOCK/BMAL1 activity, perhaps by modulating the activity of PER and CRY or the activity of corepressors (e.g. H3 K9 methylation) within repressive PER containing complexes.

While WDR5 is a well characterized MLL1 complex component, its role in repressive PER containing complexes of the mammalian circadian clock is unknown. To elucidate the functional roles of PER/WDR5 complexes, we analyzed the molecular interactions of PER1 and PER2 with WDR5 and their relationship to repressive CRY- and activating MLL1 complexes. We show that WDR5 directly binds to the C-terminal CRY-binding region (CBD) of PER1 and PER2. PER2 binds to the WBM site of WDR5 via a WBM motif within the CBD, resulting in binding competition between CRY and WDR5. In contrast, PER1 predominantly binds to the WDR5 WIN site via a WIN motif outside the CBD and exhibits a 15-fold higher affinity for WDR5 than PER2. Thereby, PER1 can form trimeric PER1/WDR5/RbBP5- and PER1/WDR5/CRY complexes potentially at the transition from the activating MLL1/WDR5/RbBP5 complex towards repressive PER/CRY containing complexes. We also show that WDR5 WIN-site inhibitors weaken WDR5-PER1 interactions in vitro. In mouse dermal fibroblast (MDF) cells, the inhibitors weaken the circadian oscillation amplitude of a PER2::Luc reporter and affect *Per2*- and *Bmal1* expression dependent on PER1 with a functional WIN motif. Together, our studies revealed a role of WDR5 as a direct PER interaction partner with critical differences between PER1 and PER2. Furthermore, our study uncovered the PER1/WDR5 complex as a target of WDR5 WIN site inhibitors, that can affect clock function by modulating the primary CLOCK/BMAL1/CRY/PER- and the secondary BMAL1/REV-ERB/ROR feedback loop of the mammalian circadian clock.

## Results

### WDR5 directly interacts with the CRY-binding regions of PER1 and PER2

To find out if WDR5 directly interacts with PER and to determine the involved interaction sites, we searched for potential WDR5 interaction motifs in the PER proteins. Interestingly, we found a potential WBM motif within the C-terminal CRY-binding domain (CBD)^19^ of mouse PER1 (^1196^L**D**VT^1199^) and PER2 (^1205^I**D**VT^1208^). PER1 additionally contains a potential WIN motif (^1110^A**R**ART^1114^) N-terminal to the CBD, that is not conserved in PER2 (Fig. 1a, b and Supplementary Fig. 3a).

Using analytical size exclusion chromatography (SEC), we showed that purified mouse WDR5 22-334 directly interacts with a C-terminal CRY binding mouse PER2 1132-1252 fragment^19^ (Fig. 1c). Likewise, a purified C-terminal mouse PER1 1013–1291 fragment including the WDR5 binding WBM- and WIN motifs forms a stable complex with WDR5 in SEC (Fig. 1d). This fragment also includes the PER1 1027-1291 region that interacts with CRY1 in cell-based Co-IP studies^21^. Hence, we identified WDR5 as a direct binding partner of PER1 and PER2, targeting the C-terminal PER region that also interacts with CRY.

### PER2 interacts with WDR5 via the WBM motif

**Fig. 2:**
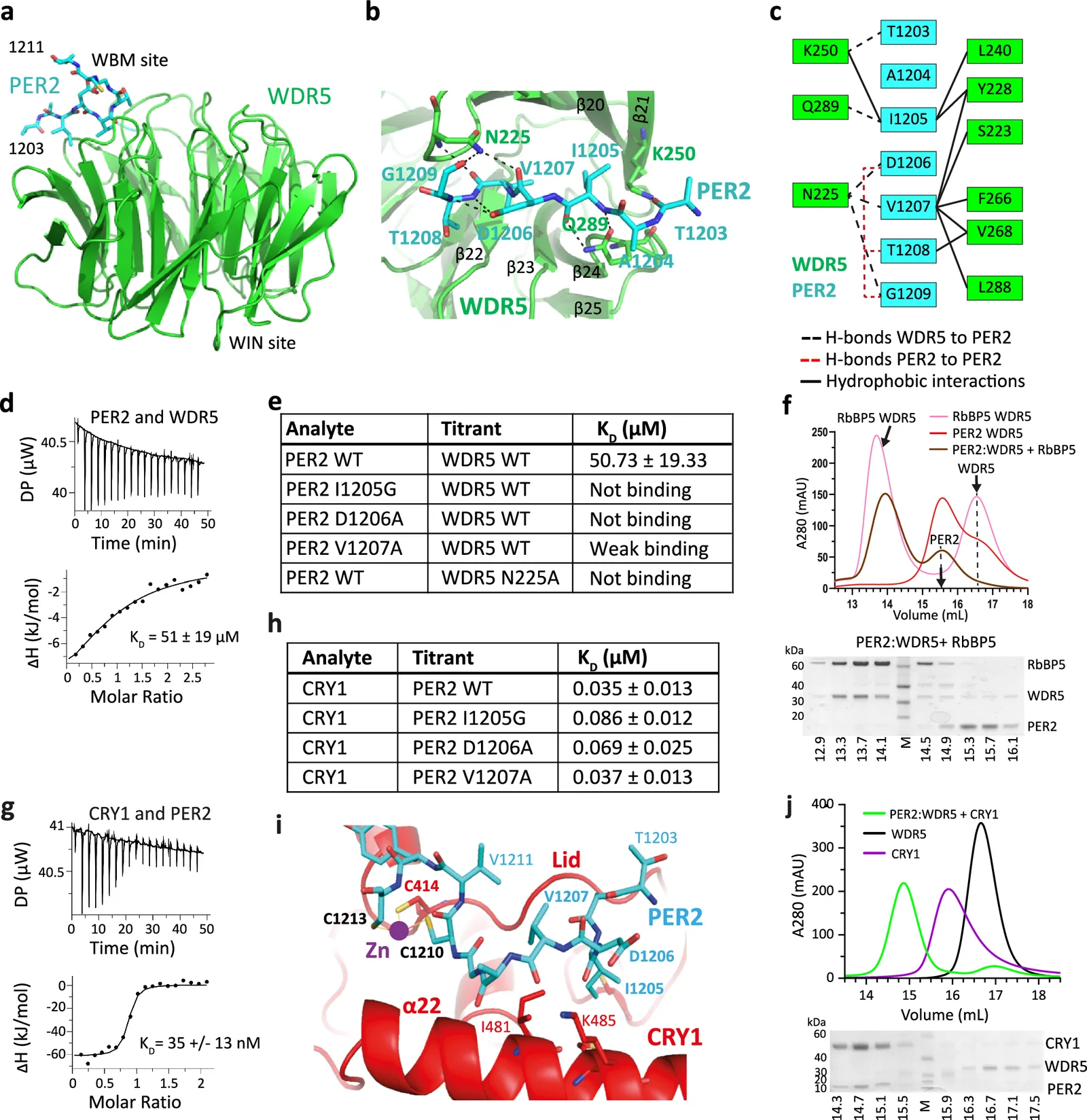
The PER2 WBM motif interacts with WDR5 and CRY1. **a**–**c** Crystal structure of WDR5 22-334 (green) bound to the PER2 1198-1211 WBM motif peptide (cyan). **a** Overview of the PER2/WDR5 complex. **b** Close-up view of the PER2/WDR5 interaction site. **c** Schematic representation of PER2-WDR5 interactions. Hydrogen bonds were defined by a maximum donor-acceptor distance of 3.1 Å, hydrophobic interactions by a maximum distance of 4.1 Å. ITC experiments of wildtype (WT) PER2 1132-1252 (CBD) with **d** WDR5 22-334 (titrant WDR5) and **g** CRY1 1-496 (PHR) (titrant PER2). Data were fit with a one-site binding model (lower panel). ITC results are representative of *n* = 3 independent experiments. ITC results (K_D_ values) for interactions of PER2 1132-1252 (WT, I1205G, D1206A, V1207A) with **e** WDR5 22-334 (WT, N225A) and **h** CRY1-PHR (mean *K*_D_ values ± SD from triplicates) (Supplementary Table 2 and Supplementary Figs. 1e–g, 2a, 3b–d). **f** Analytical SEC (S200 10/300) of PER2 1132-1252, RbBP5 and WDR5 22-334. Top: chromatograms of PER2/WDR5 (red)- and RbBP5/WDR5 (magenta) complexes (1:3 excess WDR5) and of a competition experiment of a preformed PER2/WDR5 complex (PER2:WDR5) with added equimolar RbBP5 (brown). Bottom: SDS-PAGE of the competition experiment (SEC peak fractions). No trimeric PER2/WDR5/RbBP5 complex was observed, but only the dimeric WDR5/RbBP5 complex. PER2 is displaced by RbBP5. SEC results are representative of *n* = 2 independent experiments. **i** Close up view of PER2 WBM motif region (cyan) bound to CRY1-PHR (red) (PDB: 4CT0^19^,). **j** SEC analysis (S200 10/300) of WDR5 22-334, CRY1 1-496 and PER2 1132-1252. Top: chromatograms of WDR5 (black), CRY1 (magenta) and of a preformed PER2/WDR5 complex (PER2:WDR5) mixed with equimolar CRY1 (green). Bottom: SDS-PAGE of SEC run with the PER2:WDR5 + CRY1 mixture. No trimeric PER2/WDR5/CRY1 complex was observed, but only a PER2/CRY1 complex eluting at 14.3–15.1 mL. WDR5 is displaced by CRY1 and co-elutes with monomeric WDR5 (16.3–17.5 mL). SEC results are representative of *n* = 2 independent experiments. Source data for **d**, **f**, **g**, **j** are provided as a Source data file.

To verify that PER2-CBD/WDR5 complex formation is mediated by interactions of the PER2 WBM motif with the WBM site of WDR5, we determined the crystal structure of a complex of WDR5 with a PER2 WBM motif peptide (Y^1198^TGGLPTAI**D**VTGCV^1211^) (Fig. 2a–c and Supplementary Table 1). Residues Thr1203 to Val1211 of the PER2 peptide are well resolved in the electron density (Supplementary Fig. 1a). Our 3D-structure revealed that the PER2 WBM motif ^1205^I**D**VTG^1209^ indeed binds to the WBM motif binding pocket of WDR5, inserting Ile1205 and Val1207 into its two hydrophobic sub-pockets (Fig. 2b). The side chain of the highly conserved aspartate (Asp1206 in PER2) forms intramolecular hydrogen bonds to the PER2 backbone amino groups of Thr1208 and Gly1209. Additionally, Asn225, Gln289 and Lys250 of WDR5, which are located in two WDR5 loops flanking the WBM motif binding pocket, form hydrogen bonds to the PER2 backbone carbonyl oxygens of Gly1209, Val1207, Asp1206 (Asn225) and Thr1203 (Lys250) as well as to the backbone carbonyl and amino groups of Ile1205 (Gln289) (Fig. 2b, c). Overall, the WBM motif of PER2 binds WDR5 in a similar location and orientation as the WBM motif of RbBP5^25^ (Supplementary Fig. 1b).

To show that the interaction between the PER2 WBM motif and the WBM site of WDR5 also occurs in solution and in the context of the PER2-CBD, we mutated three WDR5 interacting amino acids of PER2 (Ile1205, Asp1206, Val1207) as well as Asn225 of WDR5 to alanine (D1206A, V1207A, N225A) or glycine (I1205G) and analyzed the interactions between PER2 1232-1252 (CBD) and WDR5 22-334 (wildtype and mutants) by SEC and Isothermal titration calorimetry (ITC) (Fig. 2d, e, Supplementary Fig. 1c–g, 2a and Supplementary Table 2). Although the PER2-CBD and WDR5 form a stable complex in SEC (Fig. 1c), their interaction affinity is rather low with a *K*_D_ value of about 50 µM (Fig. 2d). The D1206A mutation completely disrupted the PER2/WDR5 complex in SEC and ITC, suggesting that the intramolecular interactions of Asp1206 are important, likely by stabilizing the PER2 conformation required for WDR5 binding. The I1205G mutation also completely disrupted the PER2-WDR5 interaction in SEC and ITC, while the V1207A mutation led to a partial dissociation of the complex in SEC and a very low WDR5 affinity (*K*_D_ about 100 µM) (Fig. 2e and Supplementary Fig. 1c–g). Furthermore, the WDR5 N225A mutation eliminates PER2 binding in ITC (Fig. 2e and Supplementary Fig. 2a), confirming that PER2 binds to the WBM site of WDR5. We also crystallized the WDR5 N225A mutant protein and could see that the overall structure of WDR5 was not affected beyond the region around the mutation site (Supplementary Fig. 2b and Supplementary Table 1). Collectively our data show that the ^1205^IDVT^1208^ WBM motif of PER2 mediates interactions of the PER2-CBD with the WBM site of WDR5.

### PER2 and RbBP5 compete for binding to the WBM site of WDR5

In the MLL1 complex, which co-activates CLOCK/BMAL1, RbBP5 binds to the WBM site of WDR5, while the WIN site is occupied by MLL1^24–28^. RbBP5 binds WDR5 with a *K*_D_ of 0.6 µM, i.e. about 80 times stronger than PER2 (Supplementary Fig. 2d). Consistent with its higher WDR5 affinity and binding competition with PER2 for the WBM site, RbBP5 displaces the PER2-CBD from a preformed PER2/WDR5 complex to form a dimeric RbBP5/WDR5 complex, and a trimeric PER2/WDR5/RbBP5 complex cannot be formed (Fig. 2f).

### The PER2 WBM motif binds CRY1 near the Zinc interface, but WBM mutations do not disrupt CRY1-PER2 interactions

Interestingly, the PER2 WBM motif is located within the CRY1-PER2 interface, between helix α22 and the “Lid” loop of the CRY1 photolyase homology region (PHR) and next to a zinc ion that is jointly coordinated by Cys1210/Cys1213 of PER2 and Cys414/His473 of CRY1 (Fig. 2i and Supplementary Fig. 3a)^19^. To elucidate the role of the WBM motif in PER2-CRY1 interactions, we performed ITC experiments with PER2 1132-1252 (WT, I1205G, D1206A, V1207A) and mouse CRY1 1-496, which includes the PHR region of CRY1, shown to be sufficient to bind PER2^19^. All PER2 proteins bound to CRY1 with a high nM affinity, with *K*_D_ values of 35 nM (wildtype), 86 nM (I1205G), 69 nM (D1206A), and 37 nM (V1207A) (Fig. 2g, h and Supplementary Fig. 3b–d). Hence, the WBM motif mutations, while weakening or disrupting WDR5-PER2 interactions, do not impact the CRY1 binding affinity much, despite the striking location of the WBM motif near the zinc interface. This is likely due to the large CRY1-PER2 interface area^19^ where the PER2 WBM motif only provides a minor contribution, as well as the much higher affinity of PER2 to CRY1 (nM) compared to WDR5 (µM).

### CRY1 and WDR5 compete for PER2 binding

To assess if WDR5 and CRY1 compete for PER2 binding due to their overlapping binding sites in the C-terminal PER2 CBD, we performed SEC experiments with a pre-purified PER2-CBD/WDR5 complex incubated with equimolar CRY1 1-496 (PHR) (Fig. 2j). A trimeric CRY1/PER2/WDR5 complex was not observed but only a dimeric CRY1/PER2 complex. Therefore, WDR5 and CRY1 indeed compete for binding to PER2. Moreover, CRY1 sequesters PER2 from a preformed PER2/WDR5 complex, consistent with its much higher affinity for PER2.

### PER1 has a higher WDR5 affinity than PER2 and forms a tight complex with CRY1

**Fig. 3:**
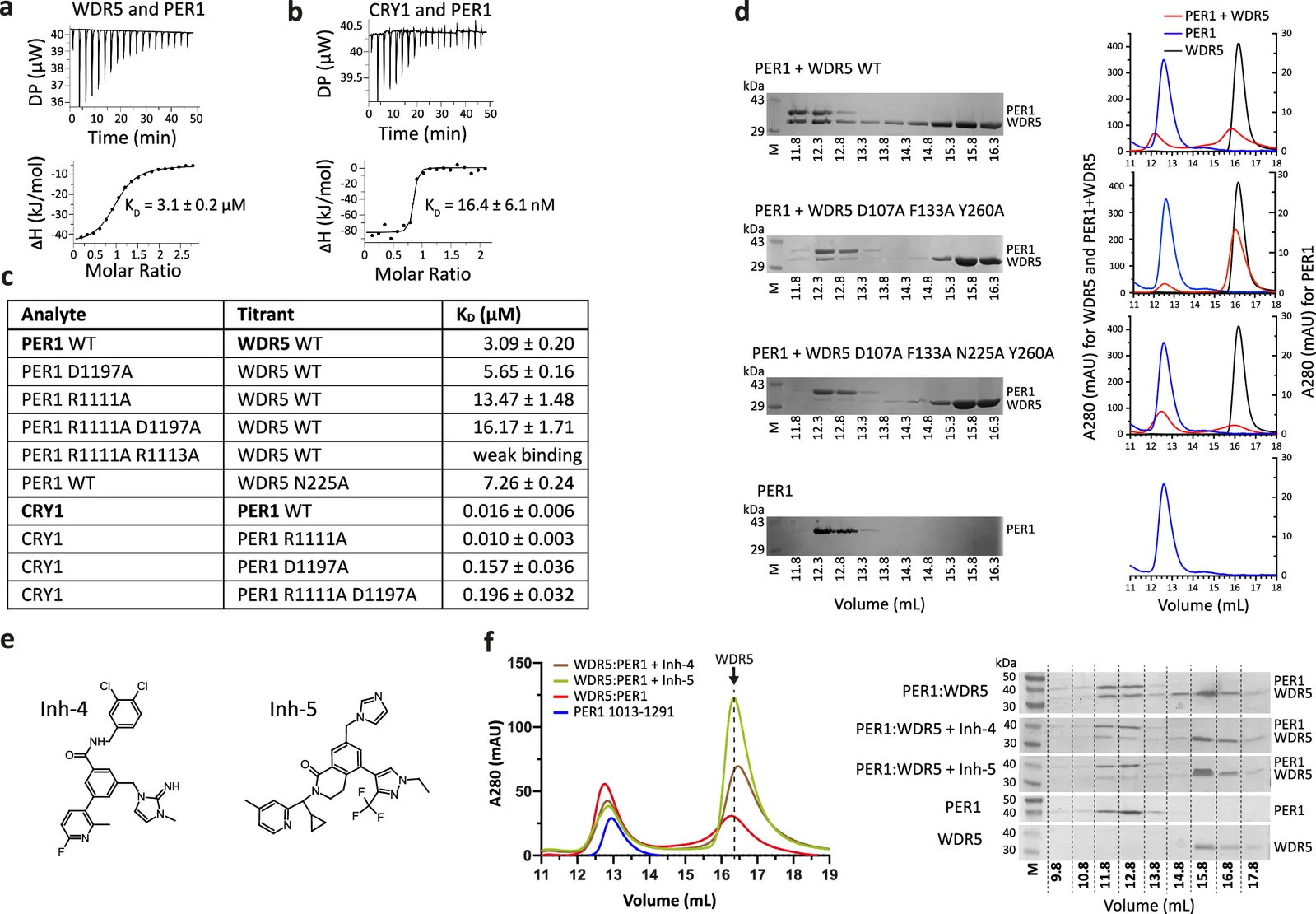
PER1/WDR5 complexes are mostly mediated by WIN site interactions and destabilized by WDR5 WIN site Inhibitors, whereas the PER1 WBM motif is involved in CRY1 binding. ITC analyses of the interaction of PER1 1013-1291 with **a** WDR5 22-334 (titrant WDR5) and **b** CRY1 1-496 (PHR) (titrant PER1). Data were fit with a one-site binding model (lower panel). ITC results are representative of *n* = 3 independent experiments. **c** ITC results (*K*_D_) for interactions of PER1 1013-1291 (WT, R1111A, D1197A, R1111A D1197A, R1111A R1113A) with WDR5 22-334 (WT, N225A) or CRY1 1-496 (mean *K*_D_ values ± SD from triplicates) (Supplementary Table 2, Supplementary Fig. 4a–d and 5a–c). ITC results are representative of *n* = 3 independent experiments. **d** SEC (S200 10/300) analyses of the interaction of PER1 1013-1291 with WDR5 22-334 WT (top), the WIN site triple mutant WDR5 D107A F133A Y260A, and the WIN/WBM site mutant WDR5 D107A F133A Y260A N225A. The SEC run of PER1 alone is shown for comparison (bottom). Left: SDS-PAGE analyses of peak SEC fractions, right: SEC chromatograms of the PER1/WDR5 complex (red), PER1 alone (blue), and WDR5 alone (black). SEC results are representative of *n* = 2 independent experiments. **e** Chemical structure of the WDR5 WIN site inhibitors Inh-4^31^ and Inh-5^32^**. f** Inh-4 and Inh-5 weaken the PER1 1013-1291 interaction with WDR5 22-334 in SEC (S200 10/300). Left: Chromatograms of the PER1/WDR5 complex without (red) and in presence of Inh-4 (brown) or Inh-5 (green). PER1 alone (blue) and WDR5 (black arrow) are shown for comparison. Right: SDS-PAGE analyses. SEC results are representative of *n* = 2 independent experiments. Source data for **a**, **b**, **d**, **f** are provided as a Source data file.

Using SEC and ITC, we showed that the C-terminal PER1 1013-1291 fragment directly interacts with WDR5 22-334 (Figs. 1d and 3a, c, d) and with CRY1 1-496 (PHR) (Fig. 3b, c). Interestingly, PER1 binds to WDR5 with a *K*_D_ value of 3.1 µM (Fig. 3a, c), which corresponds to a ~15 times higher affinity than PER2 (~50 µM) (Fig. 2d, e). Furthermore, PER1 1013-1291 binds to CRY1 with a *K*_D_ value of 16.4 nM (Fig. 3b, c), that is with a similar affinity as PER2 (Fig. 2g, h). The comparably high nM range affinities of PER1 and PER2 for CRY1 are in sharp contrast to their overall much lower (µM range) and unequal affinities for WDR5.

### The WIN site is more important for PER1-WDR5 interactions than the WBM site

To find out if the potential WBM and WIN motifs of PER1 (Fig. 1a, b and Supplementary Fig. 3a) are involved in WDR5 binding, we mutated the conserved Arg1111 (WIN) and Asp1197 (WBM) to Ala and determined the WDR5 binding affinities by ITC. The single mutations increased the *K*_D_ values to 13.5 µM (R1111A) and 5.7 µM (D1197A), respectively, the R1111A D1197A double mutant to 16.2 µM (Fig. 3c and Supplementary Fig. 4a–c). Given the rather mild effects of these mutations, we hypothesized that the second arginine, Arg1113, present in the potential WIN motif of PER1 could also play a role in WDR5 binding, akin to the KANSL1/WDR5 WIN site interaction in the non-specific lethal (NSL) complex (PDB 4CY2^30^). In the KANSL1/WDR5 complex, the first highly conserved arginine of the KANSL1 WIN motif (corresponding to Arg1111 in PER1) is inserted in the WIN motif binding pocket of WDR5 nearby Phe133, whereas the second arginine (Arg1113 in PER1) forms a salt bridge to Asp107_WDR5_ at the surface of the WDR5 WIN site (Supplementary Fig. 4e). Indeed, the R1111A R1113A double mutation drastically reduces the WDR5 affinity of the PER1 CBD (*K*_D_ > 100 µM) (Fig. 3c and Supplementary Fig. 4d), suggesting that Arg1113 is important for PER1 binding to the WDR5 WIN site. To confirm that PER1 binds to the WIN site of WDR5, we generated*—*based on the KANSL1/WDR5 complex crystal structure (Supplementary Fig. 4e)*—*a WDR5 D107A F133A Y260A triple mutant protein designed to disrupt interactions with PER1 residues Arg1111 (F133A), Arg1113 (D107A), and Thr1114 (Y260A). The triple mutant mostly disrupted the WDR5/PER1 complex in SEC (Fig. 3d), confirming the importance of the WDR5 WIN site for PER1 binding. Likewise, the simultaneous disruption of the WIN- and WBM sites of WDR5 by combining the D107A F133A Y260A triple mutant (WIN site) with the N225A mutation (WBM site) dissociated the PER1/WDR5 complex in SEC (Fig. 3d).

The moderate effects of the D1197A mutation in the PER1 WBM motif (*K*_D_ = 5.7 µM) and of the single WDR5 N225A mutation (*K*_D_ = 7.3 µM) on the PER1-WDR5 interaction affinity (Fig. 3c and Supplementary Figs. 2c, 4a) as well as the very low WDR5 affinity of the PER1 R1111A R1113A WIN site mutant (*K*_D_ > 100 µM) indicate a minor importance of the WBM site for PER1/WDR5 complex formation. This contrasts with PER2 and RbBP5, which bind WDR5 exclusively via their WBM motif, leading to a much more drastic effect of the WDR5 N225A mutation by disrupting PER2-WDR5 interactions (Fig. 2e and Supplementary Fig. 2a) and lowering the RbBP5-WDR5 affinity from a *K*_D_ value of 0.62 µM (wildtype WDR5) to 71 µM (WDR5 N225A) (Supplementary Fig. 2d, e). Together, our data show that PER1 binds to both the WIN- and the WBM motif binding pockets of WDR5, but the WIN site interaction is more important for complex formation than the WBM site.

### The PER1 WBM motif is involved in CRY1 binding

We also tested the impact of the PER1 R1111A WIN - and D1197A WBM motif mutations on CRY1 binding. The PER1 1013-1291 R1111A protein had a similar affinity for CRY1 1-496 (PHR) as wildtype PER1, whereas the D1197A mutation lead to a ten-fold higher *K*_D_ value of 157 nM. The R1111A D1197A double mutation affecting the WIN- and WBM motif resulted in a *K*_D_ value of 196 nM, comparable to the D1197A single mutation (Fig. 3c and Supplementary Fig. 5a–c). Hence, the PER1 WBM motif is involved in CRY1 binding, while the WIN motif region is not required for PER1-CRY1 interactions. This result is consistent with the predicted CRY1 binding site of PER1 (based on the CRY1/PER2 crystal structure^19^), which includes the WBM- but not the WIN motif (Fig. 2i and Supplementary Fig. 3a). Notably, the significant impact of the D1197A mutation on the PER1-CRY1 interaction (10-fold reduced affinity) contrasts with the minor effect of the analogous D1206A_PER2_ mutation on PER2-CRY1 interactions (Fig. 2h and Supplementary Fig. 3c) suggesting a more important role of this interface in the PER1/CRY1 complex.

### WDR5 WIN site inhibitors weaken the PER1/WDR5- and MLL1/WDR5 interaction

The prominent role of the PER1 WIN motif in mediating WDR5 interactions prompted us to test the effect of two WDR5 WIN site inhibitors, Inh-4,5 (Fig. 3e), that bind to the WDR5 WIN site with a high affinity^31,32^, on the PER1-WDR5 interaction by SEC (Fig. 3f). Inh-4 binds to the WDR5 WIN site with a *K*_D_ of 0.1 nM and inhibits binding of a FAM-labelled MLL1 WIN motif related 10mer peptide with a Ki value < 0.1 nM in a TR-FRET experiment^31^, whereas the further optimized Inh-5 inhibits binding of the same FAM-labelled peptide with a Ki value < 0.02 nM^32^. Additionally, Inh-5 showed improved aqueous solubility, pharmacokinetics, and oral bioavalability^32^. Both inhibitors led to a partial dissociation of the PER1/WDR5 complex and accumulation of free WDR5 (eluting at ≈16.5 ml). However, Inh5 disrupted the PER1/WDR5 complex more efficiently than Inh-4.

**Fig. 4:**
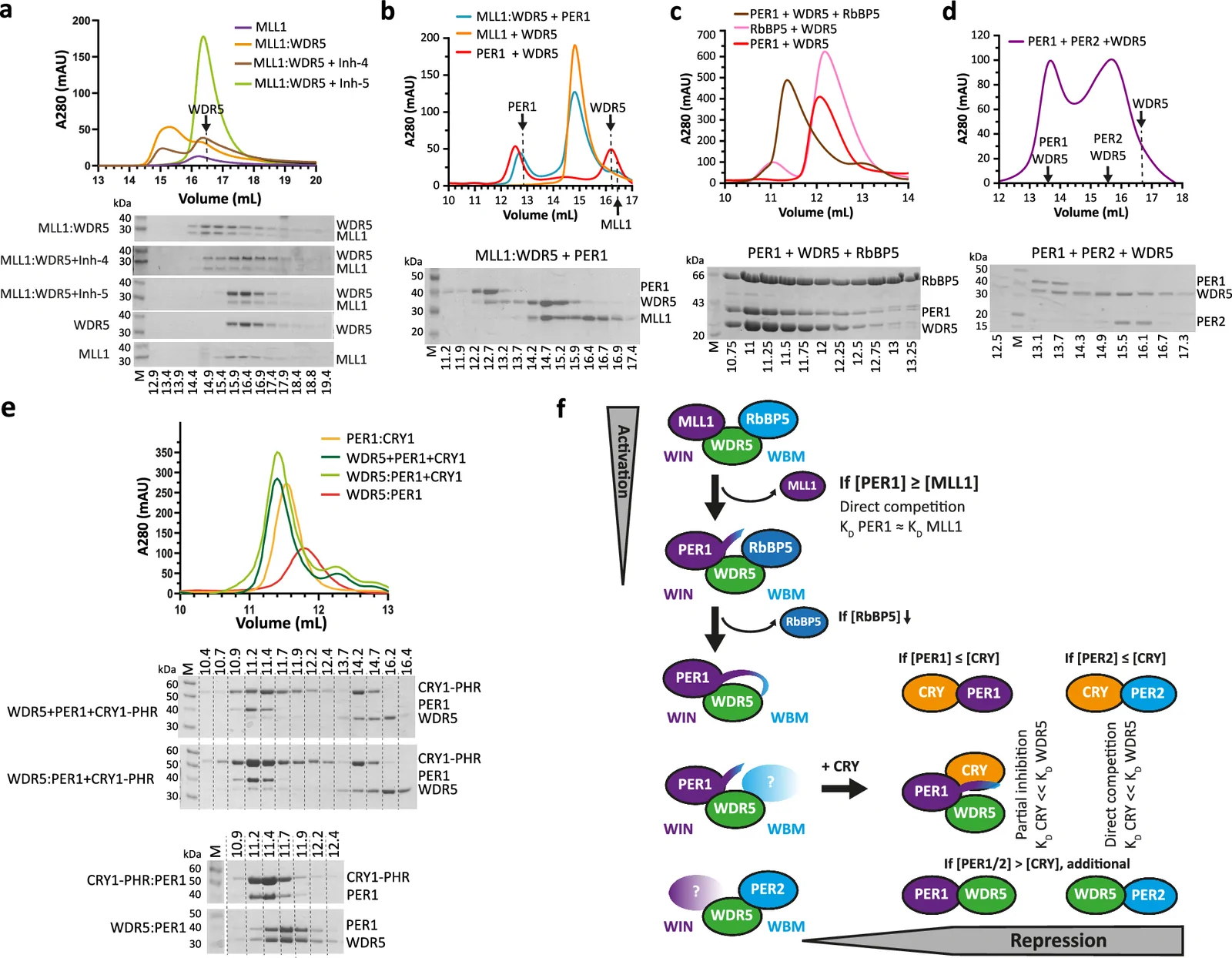
Interaction interplay between PER, WDR5, RbBP5, MLL1 and CRY. **a** Inh-5 disrupts and Inh-4 weakens the interaction of hMLL1 3748-3969 (WIN-SET) with WDR5 22-334 in SEC (S200 10/300). Chromatograms (top) and SDS-PAGE analyses (bottom) of the MLL1/WDR5 complex without (orange) and in presence of Inh-4 (brown) or Inh-5 (green). Free MLL1 (violet) and WDR5 (black arrow) are shown for comparison. SEC results are representative of *n* = 2 independent experiments. **b** Analytical SEC (S200 10/300) of WDR5 22-334, PER1 1013-1291 and hMLL1 3748-3969. Top: chromatograms of PER1/WDR5 (red)- and MLL1/WDR5 (orange) complexes and of the competition experiment of a preformed MLL1/WDR5 complex (MLL1:WDR5) with added equimolar PER1 (light blue). Bottom: SDS-PAGE of the competition experiment. Newly formed PER1/WDR5 complex elutes at ~ 12.5 mL, remaining MLL1/WDR5 complex at ~ 15 mL. PER1 partially displaces MLL1 (~16.5 mL) from the preformed MLL1/WDR5 complex. SEC results are representative of *n* = 3 independent experiments. **c** Analytical SEC (S200 10/300) of equimolar PER1 1013-1291, RbBP5 and WDR5 22-334. Top: chromatograms of PER1/WDR5 (red)-, RbBP5/WDR5 (pink) and PER1/WDR5/RbBP5 (brown) complexes. Bottom: SDS-PAGE of the trimeric PER1/WDR5/RbBP5 complex (SEC peak fractions), that eluted significantly earlier than the dimeric complexes and the individual PER1 and WDR5 proteins (Fig. 3d). Note that a different SEC S200 10/300 column was used in Fig. 3d and **c** than in all other experiments stating S200 10/300. SEC results are representative of *n* = 2 independent experiments. **d** Analytical SEC (S200 10/300) of PER1 1013-1291, PER2 1132-1252 and WDR5 22-334. Chromatogram (top) and SDS-PAGE (bottom) of the SEC run with the PER1 + WDR5 + PER2 mixture. No trimeric PER1/WDR5/PER2 complex was observed, but only dimeric PER1/WDR5- and PER2/WDR5 complexes. Arrows indicate elution volumes of individual PER1/WDR5- and PER2/WDR5 complexes and of WDR5. SEC results are representative of *n* = 2 independent experiments. **e** Chromatograms and SDS-PAGE analyses of SEC (S200 10/300) of the PER1 1013-1291 interaction with CRY1 1-496 (PHR) and WDR5 22-334. SDS-PAGE top: equimolar mixture of PER1, CRY1, and WDR5, 1 h incubation (dark green chromatogram); second: preformed PER1/WDR5 complex (ON), subsequently incubated 1 h with equimolar CRY1 (light green chromatogram). Bottom: dimeric PER1/CRY1 and PER1/WDR5 complexes (orange and red chromatograms) start to elute at 11.2 and 11.4 mL. Some trimeric PER1/WDR5/CRY1 complex is formed, that starts to elute before 11.2 ml. Non-incorporated WDR5 elutes after 16 ml. SEC results are representative of *n* = 3 independent experiments. **f** Schematic of PER-WDR5 interactions and their interplay with CRY, RbBP5 and MLL1. PER2 only binds to the WBM site of WDR5, and another protein could bind to the WDR5 WIN site. PER1 binds to both WDR5 binding sites via its WIN- and WBM motifs, but the WIN site interaction is more important. Alternatively, PER1 can only bind to the WIN site, while another protein binds to the WBM site, for example, RbBP5. Thereby, PER1 could replace MLL1 on the WIN site. PER2 binds to either WDR5 or CRY, whereas PER1 can form a trimeric PER1/WDR5/CRY complex. PER1/WDR5/RbBP5- and PER1/WDR5/CRY complexes could be formed at the transition from the active state (MLL1 bound) to the early repressed state (CRY/PER/WDR5 containing repressive complexes) of CLOCK/BMAL1. PER/WDR5 complex formation requires excess PER or spatial/temporal separation from CRY. Source data for **a**–**e** are provided as a Source data file.

As MLL1 also interacts with the WIN site of WDR5, we also tested the effect of Inh-5 and Inh-4 on MLL1/WDR5 complex formation in SEC. Inh-5 completely disrupted a preformed complex of a human MLL1 3748-3969 fragment (comprising the WDR5 binding WIN motif _3762_GSARAEV_3768_ and the adjacent catalytic SET domain of MLL1) with WDR5, whereas Inh-4 only partially disrupted this complex under SEC conditions (Fig. 4a). We note that the individual MLL1 and WDR5 proteins both elute at about 16.5 ml in SEC S200 10/300 columns. This results in co-elution of MLL1 and WDR5 at 16.5 ml after disruption of the MLL1/WDR5 complex by Inh-4 and Inh-5, whereas the intact MLL1/WDR5 complex elutes at about 15 ml.

### PER1 displaces MLL1 from WDR5 and forms a trimeric complex with WDR5 and RbBP5

In the MLL1 complex, RbBP5 binds to the WBM site and MLL1 to the WIN site of WDR5^24–28^. As both PER1 and MLL1 bind to the WDR5 WIN site and the MLL1 affinity to WDR5 also is in the µM range^24,28^, we hypothesized that PER1 and MLL1 may compete for WDR5 binding on circadian promotors. Indeed, in our analytical SEC experiments conducted with equimolar concentrations of PER1, WDR5 and MLL1, the purified WDR5-binding PER1 1013-1291 fragment partially displaced the MLL1 WIN-SET fragment (hMLL1 3748-3969) from a preformed MLL1/WDR5 complex to form a dimeric PER1/WDR5 complex (Fig. 4b and Supplementary Fig. 6). Furthermore, we observed a trimeric PER1/WDR5/RbBP5 complex in SEC (Fig. 4c), suggesting that in presence of RbBP5, PER1 can bind to the WDR5 WIN site independent of its WBM motif. Thereby PER1 could displace MLL1 from WDR5 (converting the active MLL1/WDR5/RbBP5 complex into a catalytically inactive PER1/WDR5/RbBP5 complex), when PER1 accumulates in the nucleus and its local concentration at circadian promotors becomes equal to or higher than that of the CLOCK/BMAL1 associated MLL1 complex (Fig. 4f). We note that the human hMLL1 3748-3969 WIN-SET fragment used herein only differs from the equivalent mouse MLL1 3745-3966 region by one amino acid exchange C-terminal to the WIN motif. Hence, usage of the human MLL1 homologue is unlikely to affect our results.

### A trimeric PER1/WDR5/PER2 complex is not observed in vitro

We also tested if a trimeric PER1/WDR5/PER2 complex could be formed with PER1 binding to the WIN site and PER2 to the WBM site of WDR5, which could potentially enhance the stability and specificity of a PER1/PER2 complex mediated by additional interactions, e.g., via the PAS domains^16,17,33^. Therefore, we conducted SEC experiments with our purified PER1 1013-1291, PER2 1132-1252 (CBD), and WDR5 22-334 proteins. In the SEC, we did not observe a trimeric PER1/WDR5/PER2 complex but rather found WDR5 binding to either PER1 or PER2 (Fig. 4d). The dimeric PER1/WDR5- and PER2/WDR5 complexes eluted in two well separated peaks at their expected elution volumes of about 13.5 mL (PER1/WDR5) and 15.5 mL (PER2/WDR5) (compare Figs. 1d and 2f) and earlier than free WDR5 (~16.5 mL). The absence of PER1/WDR5/PER2 trimers could be due to binding competition of PER1 and PER2 at the WDR5 WBM site.

### Trimeric PER1/WDR5/CRY complexes can be formed

As the WIN motif of PER1 is located outside of the CRY-binding region of PER1 (Supplementary Fig. 3a), we hypothesized that a trimeric CRY/PER1/WDR5 complex could be formed. Indeed, some trimeric CRY1/PER1/WDR5 complex is observed in SEC, using either equimolar mixtures of purified WDR5 22-334, PER1 1013-1291 and CRY1 1-496 proteins, or adding equimolar CRY1 to a preformed PER1/WDR5 complex (Fig. 4e). The trimeric complex starts to elute earlier than the dimeric CRY1/PER1 complex (11.2 ml) and significantly earlier than the PER1/WDR5 complex (11.4 ml). Moreover, some free WDR5 (≈15 ml), but no (or very little) dimeric PER1/WDR5 complex was observed. For the mouse CRY2-PHR (1-512), we observed a similar behavior: some trimeric CRY2/WDR5/PER1 complex was formed from equimolar CRY2:PER1:WDR5 mixtures or when CRY2 was added to a preformed PER1/WDR5 complex, and WDR5 was partly displaced (Supplementary Fig. 5d).

Given that CRY has a much higher PER1 affinity than WDR5 (Fig. 3a–c), we expect that WDR5 association (*k*_on_) is slower and/or WDR5 dissociation (*k*_off_) is faster than for CRY-PER1 interactions. It is therefore conceivable that under equimolar conditions the system equilibrates to a mixture of fast forming dimeric CRY/PER1 complexes as well as some trimeric CRY/PER1/WDR5 complexes and some free WDR5, whereas formation of PER1/WDR5 complexes is kinetically disfavored. However, if PER1 was in molar excess over CRY, for example due to an earlier nuclear accumulation in the circadian cycle, dimeric PER1/WDR5 complexes could still be formed in addition to PER1/CRY- and PER1/WDR5/CRY complexes. The relatively low amounts of PER1/WDR5/CRY complexes formed under equimolar conditions could be due to a partial inhibition of WDR5 binding to preformed CRY/PER complexes caused by the proximity of the PER1 WIN site to the PER CBD and induced folding of the CBD upon CRY binding.

Together, our data show that the unique locations of the WIN- and WBM motifs as well as different WDR5 interaction interfaces and affinities of PER1 and PER2 translate into distinct interaction interplays with CRY, RbBP5 and MLL1: while PER2 can only form dimeric complexes with either WDR5 or CRY and no trimeric PER2/WDR5/RbBP5- or CRY/PER2/WDR5 complexes due to direct binding competition (Fig. 2f, j), PER1 is able to form trimeric CRY/PER1/WDR5- and PER1/WDR5/RbBP5 complexes, and competes for WDR5 binding with MLL1 (Fig. 4f).

### The PER1 WIN motif mediates interactions of full-length PER1 with WDR5 in cells

**Fig. 5:**
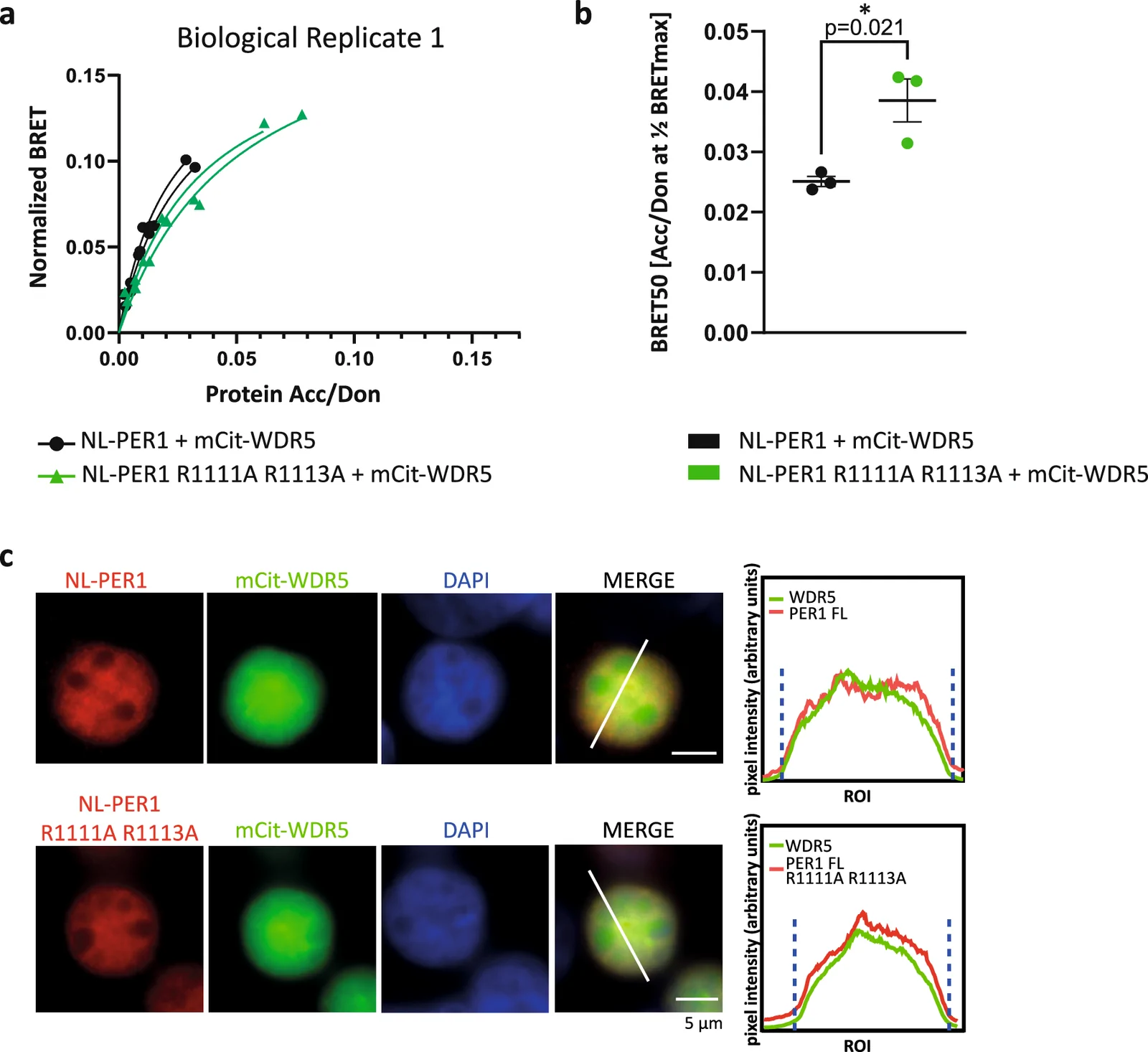
The PER1 WIN motif mediates WDR5 interactions of full-length PER1 in HEK293 cells. **a** Normalized BRET signal (1 of 3 biological replicates) of full-length NL-myc-PER1+mCit-WDR5 (black) or NL-myc-PER1 R1111A R1113A+mCit-WDR5 (green) plotted against the measured ratio of acceptor (mCitrine) over donor (NL, NanoLuc) proteins (Acc/Don). Donor:acceptor plasmid ratios were 2 ng:12.5 ng, 2 ng:25 ng, 2 ng:50 ng, 2 ng:100 ng, 1 ng:100 ng, 1 ng:200 ng. Curves were fit with a one-site specific binding model (GraphPad Prism 9.5.1). **b** BRET50 values of 3 biological replicates of wildtype or mutant PER1 (*n* = 3 biological replicates with total of *n* = 7 technical replicates) are shown as dots. Bars represent the mean values of all three biological replicates ± SEM. Two-tailed unpaired *T*-test analysis shows a *p*-value of 0.021 (*). **c** Representative immunohistochemistry staining of HEK293T cells co-transfected with NL-cmyc-PER1 or NL-cmyc-PER1 R1111A R1113A and mCit-WDR5. Red channel: cmyc-antibody, green channel: mCitrine, blue channel: DAPI staining. The lineplot visualizes co-localization of PER1 and WDR5 in the nucleus. Data are representative of two independent (*n* = 2) transfections. Similar colocalization patterns were observed across multiple fields of view in each experiment. Source data for **a**–**c** are provided as a Source data file.

To investigate the role of the PER1 WIN motif in WDR5 binding in a cellular context and within the full-length mouse PER1 protein, we performed a donor saturation Bioluminescence Resonance Energy Transfer (BRET) assay in HEK293 cells^34^. Thereby, increasing amounts (12.5, 25, 50, 100, 200 ng) of acceptor plasmid encoding mCit-WDR5 with N-terminally fused mCitrine were cotransfected with 1 or 2 ng of donor plasmids encoding either wildtype PER1 or the PER1 R1111A R1113A WIN motif mutant with an N-terminally fused NanoLuciferase (NL), in order to yield increasing acceptor/donor (Acc/Don) ratios (Fig. 5a and Supplementary Fig. 7a). We produced saturation curves for mCit-WDR5 with wildtype or mutant NL-PER1, and calculated the BRET_50_, which is defined as the ratio of the relative expression of the acceptor and donor proteins (Acc/Don) at half of the maximal BRET signal (BRET_max_) reached for the tested protein pair (Supplementary Fig. 7b). A significant correlation between BRET_50_ and K_D_ values has been reported for 25 interaction pairs, suggesting that BRET_50_ values are indicative of protein binding affinities in cells^34^. We observed that the average BRET_50_ for the interaction between wildtype NL-PER1 and mCit-WDR5 is significantly lower than for the NL-PER1 R1111A R1113A mutant and mCit-WDR5 across technical and biological replicates (Fig. 5b and Supplementary Fig. 7b right). This indicates a stronger interaction between wild-type PER1 and WDR5 compared to the PER1 WIN site mutant in cells, consistent with our ITC measurements (Fig.3c).

As WDR5 is a nuclear protein, its interaction with PER1 is expected to occur in the nucleus. Since the wildtype and mutant PER1 constructs reached similar estimated BRET_max_ signals against WDR5 (Supplementary Fig. 7b left) we assumed that the mutation did not alter the subcellular localization of PER1. To confirm this, we performed immunohistochemistry staining in HEK293T cells. Indeed, we observed a similar WDR5 co-localization within the nucleus for both the mutant and the wildtype PER1 protein (Fig. 5c and Supplementary Fig. 7c). We conclude that the PER1 WIN motif is important for the interaction of PER1 and WDR5 in a cellular context, and its mutation also weakens the WDR5 interaction in the context of full-length PER1.

### WDR5 WIN site inhibition affects the circadian clock in a PER1 dependent manner

**Fig. 6:**
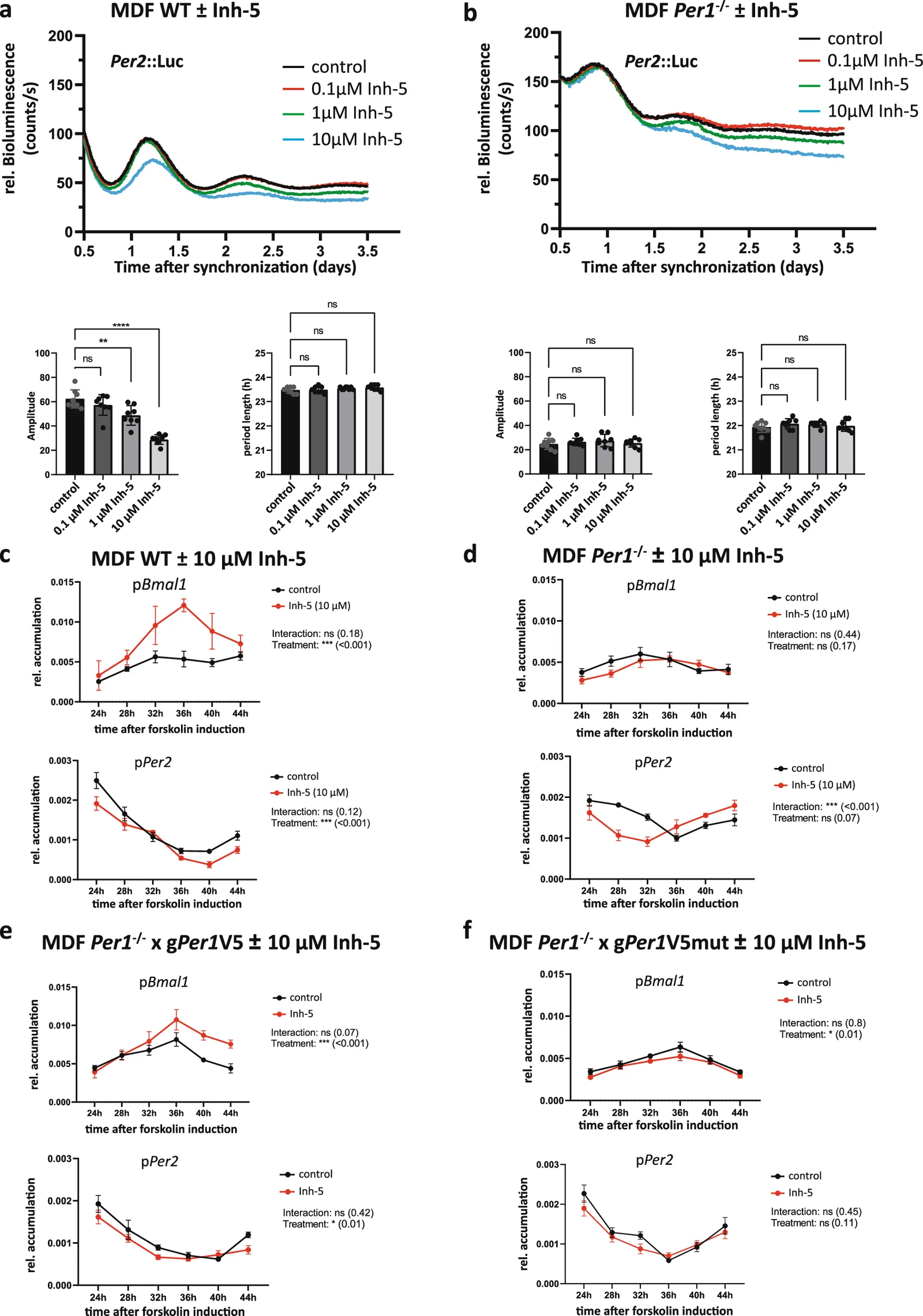
Inh-5 affects the PER2*::*Luc oscillation amplitude and *Bmal1*- and *Per2* transcription in presence of PER1 with an intact WIN motif. Normalized bioluminescence raw counts of early passage (*P* = 4) wildtype (WT) (**a**) or *Per1*^*−/−*^ (**b**) *Per2*::Luc MDF cells with and without Inh-5 (*n* = 8) (top) and the respective amplitude and period length analysis (bottom). The MDF cells were synchronized with forskolin and treated with the indicated concentrations of Inh-5. Bioluminescence was recorded for 3.5 days. Ordinary One-way ANOVA alongside Dunnett’s multiple comparison test relative to control was performed for statistical amplitude and period analysis. Amplitude of wildtype cells: *p*-values for 0.1 µM, 1 µM and 10 µM Inh-5 relative to control were *p* = 0.386 (ns), *p* = 0.002 (**), and p«0.0001 (****), respectively. Period length of wildtype cells: *p*-values for 0.1 µM, 1 µM and 10 µM Inh-5 relative to control were *p* = 0.992, *p* = 0.413, and *p* = 0.134 (ns), respectively. Amplitude of *Per1*^*−/−*^ cells: *p*-values for 0.1 µM, 1 µM and 10 µM Inh-5 relative to control were *p* = 0.736, *p* = 0.39 and *p* = 0.97 (ns), respectively. Period length of *Per1*^*−/−*^ cells: *p*-values for 0.1 µM, 1 µM and 10 µM Inh-5 relative to control were *p* = 0.377, *p* = 0.533 and *p* = 0.921 (ns), respectively. Data are presented as mean values ± SEM. Individual amplitude/period values for 8 independent treatments (*n* = 8) are shown as dots. ns=*P* ≥ 0.05, *=*P* ≤ 0.05, **=*P* ≤ 0.01, ***=*P* ≤ 0.001, ****=*P* ≤ 0.0001. qPCR data of late passage (*P* > 20) MDF wildtype cells (**c**), MDF *Per1*^−/−^ cells (**d**), and MDF *Per1*^−/−^ cells with V5-tagged wildtype PER1 (**e**) or V5-tagged PER1 R1111A R1113A (**f**) expressed from a *Per1* promoter fragment. The relative accumulation of *pPer2* and *pBmal1* pre-mRNA without Inh-5 (control, black) or in MDF cells treated with 10 µM Inh-5 (red) is plotted against the time after forskolin synchronization. Data are presented as mean ± SEM of *n* = 4 independent biological replicates. Two-way ANOVA was employed to test for treatment and interaction *P*-values. For non-significant effects (*ns*, *P* ≥ 0.05) and effects with *P* ≤ 0.05 (*) exact *P*-values are shown in brackets. ns=*P* ≥ 0.05, *=*P* ≤ 0.05, **=*P* ≤ 0.01, ***=*P* ≤ 0.001. Source data for **a**–**f** are provided as a Source data file.

To identify a functional role of WDR5 and the PER1/WDR5 complex in the circadian clock, we performed a PER2::Luc bioluminescence reporter assay in mouse dermal fibroblast (MDF) cells in absence and presence of the WDR5 WIN site inhibitor Inh-5^32^. These MDF cells express a PER2-Luciferase fusion protein and therefore the bioluminescence signal reflects the circadian expression, accumulation and degradation of the PER2 protein^35^. After forskolin synchronization, wildtype MDF cells (early passage, *P* = 4) treated with 1 µM Inh-5 or 10 µM Inh-5 showed a decrease of the relative bioluminescence signal (raw counts) compared to the DMSO control. These effects were not observed in cells treated with 0.1 µM Inh-5 (Fig. 6a top). To analyze the amplitude and period length differences between the different treatments (Fig. 6a bottom), we used a baseline corrected transformation of the data. We found that the inhibitor reduced the amplitude of peak one and two in a dose-dependent manner, up to about 50% in presence of 10 µM Inh-5. The period length was not affected at any of the tested inhibitor concentrations.

To test if the effect of Inh-5 on the circadian clock is dependent on PER1, we generated *Per1*^−/−^ knockout MDF *Per2::*Luc cells from *Per1*^−/−^ mice^36^, where *Per1* is not expressed (Supplementary Fig. 9b). Our *Per1*^−/−^ MDF cells (passage 4) showed weaker circadian rhythms and a dampened oscillation amplitude (Fig. 6b) compared to wildtype passage 4 MDF cells (Fig. 6a). Additionally, our *Per1*^−/−^ MDF cells showed a shorter period than wild-type MDF cells (Fig. 6a, b), as reported earlier for *Per1*^−/−^ fibroblasts obtained from *Per1*^Brdm/Brdm^ mice^12,14^. As in wildtype MDF cells, Inh-5 treatment of *Per1*^−/−^ MDF cells reduced the relative bioluminescence signal of the PER2::Luc reporter but did not affect the period length (Fig. 6b). Different from wildtype MDF cells, however, Inh-5 did not affect the oscillation amplitude of the *Per1*^−/−^ MDF *Per2::*Luc cells, as derived from their weaker but still discernable oscillations between days 0.5 and 2.5 (peak 1 and 2) (Fig. 6b). Our experiments suggest that the effect of Inh-5 on the oscillation amplitude of wildtype MDF cells needs functional PER1.

To further assess the effects of Inh-5 on circadian gene regulation, we performed qPCR experiments targeting the unspliced precursor RNA (p/pre-mRNA) and mature mRNA of *Bmal1*, *Per2*, and *Per1* in later (>20) passage MDF cells. While qPCR experiments targeting the unspliced precursor mRNA directly measure transcriptional effects, mature mRNA experiments report transcriptional and post-transcriptional effects. Different from the PER2::Luc oscillation experiment, the qPCR experiments do not report effects related to PER2 protein accumulation and degradation.

Using MDF cells that can overexpress *Wdr5* under the control of a doxycycline-regulated expression system, we found that *Wdr5* overexpression influences the relative pre-mRNA (“*pBmal1, pPer2, pPer1”*) and mRNA (“*Bmal1, Per2, Per1”*) accumulation of *Bmal1*, *Per2* and *Per1*, with a clear dampening effect on *(p)Bmal1* (Supplementary Fig. 8a). A similar effect of transient *Wdr5* overexpression on *Bmal1* expression had already been described^7^. Inh-5 treatment, however, eliminates the *Wdr5* overexpression effects on the *Bmal1, Per2,* and *Per1* (pre-) mRNA (Supplementary Fig. 8b). This suggests that WDR5 plays a role in the circadian clock and Inh-5 negates its effect.

Next, we tested the effect of Inh-5 on *(p)Per2* and *(p)Bmal1* (pre-)mRNA using later (>20) passage wildtype and *Per1*^*−/−*^ MDF cells that do not overexpress *Wdr5*. Despite their somewhat weaker circadian rhythms (Supplementary Fig. 10c, d) compared to early passage MDF cells (Fig. 6a, b), we used later (>20) passage *Per2::*Luc MDF cells for all our qPCR experiments, because late passage numbers were required to obtain *Per1*^−/−^ rescue cell lines with wildtype and mutant PER1 by lentiviral infection and selection (see below, Fig. 6e, f and Supplementary Fig. 9c, d). As observed in early passage *Per2::*Luc MDF cells (Fig. 6a, b), Inh-5 did not affect the period length of the late passage MDF cells, while it reduced the oscillation amplitude of wildtype MDF cells, but apparently not in *Per1*^−/−^ MDF cells (Supplementary Fig. 10c, d). We note that our conclusions about the lacking effect of Inh-5 on the circadian oscillation amplitude of the later passage *Per1*^−/−^ MDF cells are less reliable due to their weak circadian rhythms, but they are backed up by the statistically more significant observations in the early passage MDF cells (Fig. 6b). The decrease of the relative PER2::Luc bioluminescence signal (raw counts) was observed in Inh-5 treated late passage wildtype MDF cells, but barely discernable in Inh-5 treated late passage *Per1*^−/−^ MDF cells, likely due to the overall decline of the PER2::Luc signal (Supplementary Fig. 10c, d).

In our qPCR experiments Inh-5 treatment led to somewhat decreased levels of *(p)Per2* (pre-)mRNA (Fig. 6c and Supplementary Fig. 9a), which could be interpreted as enhanced repression or reduced activation of CLOCK/BMAL1. Additionally, Inh-5 treatment led to a significant increase of *pBmal1* and *Bmal1* RNA (Fig. 6c and Supplementary Fig. 9a), suggesting an effect on the regulation of *Bmal1* expression, potentially via the interlocked feedback loops involving REV-ERB, ROR, and DBP proteins^2^. Within our MDF *Per1*^−/−^ knockout cell line, however, Inh-5 treatment did not significantly affect (*p)Per2* (pre-) mRNA levels (Fig. 6d and Supplementary Fig. 9b). Furthermore, Inh-5 did not lead to an increased accumulation of (*p)Bmal1* but rather resulted in a somewhat reduced *Bmal1* mRNA accumulation in *Per1*^−/−^ MDF cells (Fig. 6d and Supplementary Fig. 9b). We note that for *pPer2* pre-mRNA, the two-way ANOVA test reports a significant interaction *p*-value between untreated and Inh-5 treated *Per1*^−/−^ MDF cells, which was however not observed for the *Per2* mRNA levels (Supplementary Fig. 9b).

To confirm that the reduced effects of Inh-5 on *(p)Per2* and *(p)Bmal1* (pre-)mRNA levels observed in our *Per1*^*−/−*^ MDF cells are not just a consequence of their weaker circadian rhythms, but rather reflect a dependance on functional PER1, we performed rescue experiments using later (>20) passage *Per1*^−/−^ MDF cells with genomically integrated V5-tagged PER1 under the control of its native *Per1* promoter^37^. To determine the contribution of the PER1 WIN motif, we tested the rescue ability of wildtype PER1 (referred to as gPER1V5) and of mutant PER1 (referred to as gPER1V5mut) carrying the R1111A R1113A WIN motif mutation, which disrupts the PER1 interaction with WDR5 in ITC experiments (Fig. 3c and Supplementary Fig. 4d) and weakens the WDR5 interaction of full-length PER1 in HEK293 cells (Fig. 5a, b and Supplementary Fig. 7a, b). Detection of *Per1* mRNA levels, but no *pPer1* pre-mRNA levels document the successful transfection with wildtype or mutant *Per1* rescue plasmids (Supplementary Fig. 9c, d).

We found that wildtype PER1 partially rescues the increase of *(p)Bmal1 a*nd the decrease of *(p)Per2* (pre-)mRNA, that we observed in wildtype MDF cells (Fig. 6e and Supplementary Fig. 9c). Both effects are not as pronounced as in the wildtype MDF cells, possibly due to the about 50% lower levels of *Per1* mRNA observed in rescued *Per1*^−/−^ MDF cells (Supplementary Fig. 9c) compared to wildtype MDF cells (Supplementary Fig. 9a). Nevertheless, the rescue experiment shows that Inh-5 affects the *Per2* and *Bmal1* RNA levels in a PER1-dependent manner. Interestingly, in presence of PER1 with the R1111A R1113A WIN motif mutation, Inh-5 treatment did not significantly affect *(p)Per2* (pre-)mRNA accumulation and led to a significant decrease of *pBmal1* levels, as observed in *Per1*^−/−^ MDF cells. Hence, mutant PER1 does not rescue the effects of Inh-5 on *(p)Per2* and *(p)Bmal1 (*pre-)mRNA observed in wild-type MDF cells (Fig. 6f and Supplementary Fig. 9d). Thus, the effects of Inh-5 on *(p)Per2* and *(p)Bmal1* (pre-)mRNA levels depend on PER1 with an intact WIN motif and therefore most likely reflect an impact of Inh-5 on the PER1 interaction with the WIN site of WDR5.

### Effects of WDR5 WIN site Inhibitor 4 and WBM site Inhibitor 6 on circadian oscillations

Consistent with its less potent effect on the purified PER1/WDR5- and MLL1/WDR5 complexes in our SEC studies (Figs. 3f and 4a), the WDR5 WIN site inhibitor 4 (Inh-4) was less efficient in reducing the relative bioluminescence signal and amplitude of the PER2::Luc oscillations in later (>20) passage MDF cells compared to Inh-5 (Supplementary Fig. 10a, c). Inh-4 only displayed its effects at 10 µM concentration, but not at 1 µM concentration (Supplementary Fig. 10a). Like Inh-5, Inh-4 did not affect the period length. Furthermore, the effect of Inh-4 on the PER2::Luc oscillation signal and amplitude was not observed in *Per1*^−/−^ MDF cells (Supplementary Fig. 10b). While our conclusions about Inh-4 are limited by the weak circadian rhythms of the late passage *Per1*^−/−^ MDF cells, the suggested PER1 dependance of the Inh-4 effect on circadian amplitude is consistent with the observed effect of Inh-5 (Fig. 6a, b).

In U-2 OS cells with a *Bmal1::*Luc reporter, which express luciferase from a murine *Bmal1* promotor^14^, Inh-4 treatment increased the magnitude of the *Bmal1:*:Luc oscillation in a dose dependent manner (Supplementary Fig. 11a) but did not significantly affect the circadian oscillation amplitude or period (Supplementary Fig. 11b, c).

Our qPCR analysis showed that Inh-4 does not affect the *pPer2* pre-mRNA levels but reduces *Per2* mRNA. Additionally, Inh-4 enhances *(p)Bmal1* RNA accumulation, but not as strongly as observed for Inh-5 (Supplementary Figs. 11d, 9a and Fig. 6c). The enhanced (*p)Bmal1* levels in MDF cells are consistent with the increased oscillation magnitude of the *Bmal1:*:Luc reporter in Inh-4 treated U-2 OS cells.

In *Per1*^−/−^ MDF cells the effect of Inh-4 on the mature *Per2* mRNA is abolished, whereas (*p)Bmal1* mRNA levels are somewhat reduced, as observed for Inh-5 (Supplementary Fig. 11e). Based on our validation of the equivalent effects of Inh-5 by rescue experiments (Fig. 6c–f and Supplementary Fig. 9) we conclude that the effects of Inh-4 on *Per2* and (*p)Bmal1* mRNA that we observed in wildtype MDF cells also depend on the presence of functional PER1.

In contrast to the WIN site inhibitors (Inh-4,5), the WDR5 WBM site inhibitor 6 (Inh-6)^38^ reduced the oscillation amplitude and the bioluminescent signal of both wildtype and *Per1*^*−/−*^ MDF cells (passage >20) at 10 µM concentration, indicating that these effects are not dependent on PER1 (Supplementary Fig. 10e–g). This is consistent with our finding that PER1 predominantly binds WDR5 via the WIN site and the WBM site of WDR5 (targeted by Inh-6) is of minor importance for the PER1/WDR5 interaction. The effect of Inh-6 also documents that our *Per1*^−/−^ MDF cells can selectively respond to inhibitors, that affect clock function in a PER1 independent manner. Thereby, the Inh-6 experiment further supports the significance of the lacking response of *Per1*^−/−^ MDF cells to the WIN site inhibitors Inh-4 and Inh-5, that affect the circadian clock in a PER1 dependent manner. Like Inh-4 and Inh-5, Inh-6 did not affect the period length.

## Discussion

### WDR5 interactions of PER1 and PER2 and their interplay with CRY/PER- and MLL1/WDR5 complexes

To provide molecular mechanistic insights into the role of PER/WDR5 interactions in the mammalian circadian clock, we investigated the molecular interactions of WDR5 with PER1 and PER2 as well as their interplay with CLOCK/BMAL1 activating MLL1/WDR5- complexes and with repressive PER/CRY complexes. We found that the C-terminal PER1 and PER2 regions, including the CRY binding domain (CBD), directly bind to WDR5 (Fig. 1), suggesting a possible crosstalk between PER/WDR5- and PER/CRY interactions. PER2 binds to the WDR5 WBM site with a low ~50 µM affinity (Fig. 2). In contrast, PER1 predominantly interacts with the WDR5 WIN site with a minor contribution of the WBM site interaction, leading to a 15-fold higher WDR5 affinity of PER1 (*K*_D_ ~3 µM) (Fig. 3). Using a BRET assay, we could confirm that the PER1 WIN motif is involved in WDR5 binding of full-length PER1 in HEK293 cells (Fig. 5). We also identified two WDR5 WIN site inhibitors (Inh-4, Inh-5) that weaken the interaction of WDR5 with PER1 and with the WIN-SET domain of MLL1 in SEC experiments (Fig. 3e, f and Fig. 4a).

Notably, PER1 and PER2 have a much higher (nM) affinity to CRY1 than to WDR5 (µM) (Figs. 2e, h and 3c). In the circadian clock, PER/WDR5 complex formation would therefore require a molar excess of nuclear PER over CRY, a temporal or spatial separation of PER/WDR5- and PER/CRY complexes and/or additional regulatory processes that enhance the PER/WDR5- or reduce PER/CRY affinity in vivo. Interestingly, the PER WBM motif binds CRY1 nearby a Zinc interface of the CRY1/PER2 complex (Fig. 2i)^19^, suggesting that the interplay between CRY/PER2- and WDR5/PER2 complexes could be regulated in a redox-dependent manner.

As the WBM motif of PER1/2 lies within the PER/CRY complex interface, whereas the PER1 WIN motif is located N-terminal to the CBD (Figs. 1a and 2i), PER1 can form some trimeric complexes with CRY1/2 and WDR5 (Fig. 4e and Supplementary Fig. 5d), while PER2 cannot (Fig. 2j). We propose that PER1/WDR5/CRY-, PER1/WDR5- and PER2/WDR5 complexes are formed at the transition from active CLOCK/BMAL1 towards repressive CRY/PER containing complexes and could provide an entry route for WDR5 into the early repressive complex upon its assembly on circadian promotors (Supplementary Fig. 12). As PER1 is reported to arrive at circadian promotors before PER2, and CRY2 before CRY1^4,7,9^, it is conceivable that varying nuclear concentrations of these clock components will lead to different combinations of PER1/CRY/WDR5-, PER/WDR5- and PER/CRY complexes at different circadian times, where each complex has the potential for distinct activities and interaction networks, e.g. by recruiting different chromatin modifiers^9^. Direct analyses of the temporal coordination of endogenous PER/WDR5-, PER/CRY- and PER1/WDR5/CRY complexes in rhythmic cells are technically challenging, as SEC analyses are hampered by the fact that WDR5 forms many multiprotein complexes with similar sizes as PER/CRY containing complexes^7^ and Co-IPs of PER1/2 and WDR5 are very inefficient even for overexpressed proteins due to the low µM affinity. However, the existence of different PER/CRY interactions was suggested by genetic experiments, where loss of *Cry2* could rescue the lack of *Per2*, while the loss of *Cry1* rendered *Per1*-deficient mice arrhythmic^39,40^.

Interestingly, we did not observe a trimeric PER1_WIN_/WDR5/PER2_WBM_ complex in our SEC experiments (Fig. 4d). As the nuclear WDR5 concentration likely exceeds nuclear PER1- and PER2 concentrations^41^, individual PER1/WDR5- and PER2/WDR5 complexes may coexist at circadian promotors, provided that the local PER1/2 concentrations are higher than the CRY1/2 concentrations (see above) (Fig. 4f and Supplementary Fig. 12). Furthermore, PER1_WIN_/WDR5- and PER2_WBM_/WDR5 complexes have the potential to interact with distinct additional proteins, either at the WBM site of WDR5 (PER1_WIN_/WDR5/X_WBM_) or the WDR5 WIN site (Y_WIN_/WDR5/PER2_WBM_). For example, PER1 (but not PER2) can form a trimeric PER1_WIN_/WDR5/RbBP5_WBM_ complex with the WBM-site binding MLL1 complex component RbBP5 (Figs. 4c and 2f). Thereby PER1 could*—*due to its early accumulation in the nucleus^4,7^*—*displace MLL1 from the WDR5 WIN site, as soon as the nuclear PER1 concentration exceeds the local concentration of CLOCK/BMAL1-associated MLL1 complex (Supplementary Fig. 12). We therefore propose that the PER1/WDR5/RbBP5 complex succeeds the MLL1/WDR5/RbBP5 activation complex and terminates MLL1-dependent histone H3 K4 tri-methylation and transcriptional activation of circadian genes to allow the timely progression towards a repressive chromatin state. While PER2- and RbBP5 binding to the WDR5 WBM site are mutually exclusive (Fig. 2f) and the RbPB5 affinity to WDR5 is much higher than that of PER2 (Fig. 2d, e and Supplementary Fig. 2d), WDR5/RbBP5- and WDR5/PER2 complexes may*—*from a protein biochemical point of view*—*coexist, as the cellular WDR5 concentration is about 10 times higher than the RbBP5 concentration^41^. However, RbBP5 mRNA levels cycle in antiphase to PER and CRY^42^, suggesting that RbBP5 protein levels decline before PER1 and PER2, leaving binary PER1/WDR5- and PER2/WDR5 complexes behind (Supplementary Fig. 12).

### PER1/WDR5 WIN site interactions impact the primary and secondary feedback loop

Given that PER1 has a higher (possibly physiologically more relevant) WDR5 affinity than PER2 and a more versatile interaction network with activating MLL1- and repressing CRY/PER containing complexes, we focused our functional analyses on WIN site mediated PER1/WDR5 interactions in the circadian clock. We found that Inh-5 reduced the circadian oscillation amplitude of the PER2::Luc reporter in early passage 4 wildtype MDF cells, but not in *Per1* ^−/−^ MDF cells (Fig. 6a, b), suggesting that Inh-5 effects on the circadian amplitude depend on PER1. Inh-4 also reduced the PER2::Luc oscillation amplitude in later (>20) passage wildtype MDF cells, but less efficiently than Inh-5 (Supplementary Fig. 10a, c).

We also observed a general reduction of reporter gene activity in early passage wildtype- and *Per1*^−/−^ MDF cells in presence of 1 µM and 10 µM Inh-5 (Fig. 6a, b), that appears to be PER1 independent. We hypothesize that Inh-5 reduces bioluminescence activity by weakening the MLL1 interaction with the WDR5 WIN site (Fig. 4a), which would lead to reduced histone methyl transferase activity of the MLL1 containing complex^31^ and thereby reduced co-activation of CLOCK/BMAL1-dependant PER2::Luc reporter activity^5^. Likewise, the WDR5 WBM-site inhibitor Inh-6 might reduce the overall PER2::Luc reporter activity and the circadian oscillation amplitude in a PER1 independent manner (Supplementary Fig. 10e, f) by reducing the activity of the MLL1 complex, in this case by weakening the interaction of the MLL1 complex component RbBP5 with the WBM site of WDR5. Alternatively, the potential interference of Inh-6 with the PER2/WDR5 interaction could cause a PER1 independent effect on the reporter gene activity. The relevance of PER2 and its potential interaction with WDR5 (which is 15 times weaker than the PER1/WDR5 interaction) remains to be confirmed in vivo.

Using qPCR we showed that WDR5 overexpression reduces the levels of precursor (p) mRNA and mRNA of *Bmal1* and impacts the circadian *Per1* and *Per2* (p)mRNA accumulation in MDF cells (Supplementary Fig. 8). Notably, these effects are not observed in presence of Inh-5, suggesting that WDR5 overexpression exerts its effect on clock gene expression via WIN site interactions. The reduction of *Bmal1* mRNA levels further suggests that WDR5 plays a role in the secondary loop, where *Bmal1* expression is activated by the nuclear receptor ROR and repressed by REV-ERB^3^ (Supplementary Fig. 12).

In wildtype MDF cells, treatment with Inh-5 significantly increased *Bmal1* (p)mRNA levels, and somewhat decreased *Per2* (p)mRNA levels. In *Per1*^−/−^ MDF cells however, Inh-5 decreased *Bmal1* mRNA levels, and had no significant effect on *Per2* mRNA (Fig. 6c, d and Supplementary Fig. 9a, b). Treatment with Inh-4 showed the same effects (Supplementary Fig. 11d, e), just less pronounced as with Inh-5. The reduced effect of Inh-4 on *(p)Per2* and *(p)Bmal1* (pre)mRNA levels and PER2*::*Luc oscillations compared to Inh-5 is consistent with the lower efficiency of Inh-4 in disrupting PER1/WDR5- and MLL1/WDR5 complexes in SEC (Figs. 3f and 4a), suggesting a functional correlation between PER1/WDR5*—*and/or MLL1/WDR5 complex stability and circadian gene expression and oscillations. Furthermore, the increased *Bmal1* p-mRNA levels after Inh-4 treatment are consistent with the increased magnitude of *Bmal1:*:Luc oscillations in U-2 OS cells (Supplementary Fig. 11a).

Rescue of *Per1*^−/−^ MDF cells with wildtype PER1 partially restored the Inh-5 effects observed in wild-type MDF cells (increased *Bmal1* and decreased *Per2* (p)mRNA). In contrast, rescue of *Per1*^−/−^ MDF cells with PER1 carrying the R1111A/R1113A WIN site mutation, which weakens the PER1-WDR5 interaction in vitro (ITC) and in cells (Figs. 3c, 5a/b and Supplementary Figs. 4d, 7a/b), was not successful. Hence, the effects of Inh-5 on *Bmal1-* and *Per2* (p)mRNA observed in wild-type MDF cells require PER1 with an intact WIN motif. We therefore propose that PER1/WDR5 WIN site interactions play a critical role in regulating the primary CLOCK/BMAL1/CRY/PER feedback loop (readout *Per2* (p)mRNA) as well as the secondary BMAL1/REV-ERB/ROR loop (readout *Bmal1* (p)mRNA) (Supplementary Fig. 12). The more pronounced effect of Inh-4/5 on *Bmal1* (p)mRNA suggests that PER1/WDR5 interactions affect transcriptional and post-transcriptional processes in the secondary loop more strongly than in the primary loop.

### Role of WDR5 and PER1/WDR5 interactions in the primary CLOCK/BMAL1/PER/CRY feedback loop

The reduced *Per2* expression in presence of Inh-5 could in principle be due to reduced MLL1-dependant CLOCK/BMAL1 coactivation. However, as the Inh-5 effect on *Per2* (p)mRNA levels is eliminated in *Per1*^−/−^ MDF cells and rescued by wildtype PER1 but not by PER1 with a mutated WIN motif (Fig. 6c–f and Supplementary Fig. 9), it is more likely that Inh-5 reduces *Per2* expression by weakening the WIN site dependent PER1/WDR5 complex formation (Fig. 3f) and thereby enhancing CLOCK/BMAL1 repression. This implies that PER1/WDR5- or PER1/WDR5/CRY complexes negatively affect CLOCK/BMAL1 repression, perhaps by interfering with the formation or activity of repressive PER- or CRY containing complexes or with repressive chromatin modifications. Additionally, WDR5 overexpression may affect the circadian variation of *Per1* and *Per2* mRNA (Supplementary Fig. 8) by changing the relative concentration of CLOCK/BMAL1 activating or repressing WDR5 WIN site complexes.

### Role of WDR5 and PER1/WDR5 interactions in the secondary BMAL1/REV-ERB/ROR feedback loop

The significantly enhanced *Bmal1* (p)mRNA accumulation that we observed upon Inh-5 treatment of wildtype MDF cells (Fig. 6c) is likely caused by effects on the secondary feedback loop, either by reducing REV-ERB dependent *Bmal1* repression or by enhancing ROR dependent *Bmal1* activation (Supplementary Fig. 12). As *Rev-erb* expression is regulated by CLOCK/BMAL1, Inh-5 could in principle enhance *Bmal1* expression by disrupting the co-activating MLL1/WDR5 complex on the *Rev-erb* promotor. However, the Inh-5 effect on *Bmal1* (p)mRNA levels is completely eliminated in *Per1*^−/−^ MDF cells and rescued by wildtype PER1 but not by PER1 with a mutated WIN motif (Fig. 6c–f and Supplementary Fig. 9), suggesting that the effect primarily depends on PER1 with a functional WIN motif. Reportedly, PER1 or PER2 overexpression reduces *Rev-erb* expression by repressing the CLOCK/BMAL1 activity on the E-box element in the *Rev-erb* promotor^43^, which should enhance *Bmal1* expression. Our RT-PCR data suggest that an intact PER1/WDR5 complex represses *Bmal1,* and this repression is released by Inh-5-dependent disruption of the PER1/WDR5 complex, resulting in *Bmal1* activation (Fig. 6c, e and Supplementary Fig. 9a, c). Possibly, the PER1/WDR5 complex inhibits the PER1 dependent repression of *Rev-erb*, akin to the proposed negative effect of PER1/WDR5 complexes on CLOCK/BMAL1 repression in the primary loop (see above). Inh-5 treatment would then release PER1 to efficiently repress *Rev-erb* and thereby enhance *Bmal1* expression (Supplementary Fig. 12). Conversely, WDR5 overexpression would recruit more PER1 (and PER2) into PER/WDR5 complexes, which should enhance *Rev-erb*- and reduce *Bmal1* expression, consistent with the decreased *Bmal1* (p)mRNA levels that we observed in WDR5 overexpressing MDF cells (Supplementary Fig. 8). Additionally, WDR5 overexpression could enhance *Rev-erb* expression by stabilizing the CLOCK/BMAL1 activating MLL1/WDR5 complex.

A second mechanism, how PER1 could, after its Inh-5 dependent release from PER1/WDR5 complexes, increase *Bmal1* expression, would be a PER1 dependent co-activation of ROR (Supplementary Fig. 12). PER1 has an LXXLL motif in its C-terminal region (^1041^L L E L L^1045^)^43^, which allows co-activators to bind to nuclear receptors and could therefore mediate PER1 interactions with ROR. As this motif is not too far away from the WDR5 binding WIN motif of PER1 (^1110^ARART ^1114^), it is possible that PER1/WDR5 complex formation blocks binding of PER1 to ROR. In WDR5 overexpressing cells, this effect could be even more pronounced, leading to a net reduction of *Bmal1* expression (Supplementary Fig. 8). Our study suggests further investigations of this proposed mechanism, e.g., by analyzing the role of ROR interactions with PER1 and with the PER1/WDR5 complex in vitro and in vivo.

### Potential relevance of additional WDR5 WIN site ligands

Our observation that *Bmal1* mRNA is reduced upon Inh-5 treatment of *Per1*^−/−^ MDF cells (Supplementary Fig. 9b) has to be explained by effects on WDR5 WIN site ligands other than PER1, which could result in enhanced REV-ERB dependent repression or reduced ROR dependent activation of *Bmal1*. As *Ror* expression is activated by the D-box binding protein (DBP), and CLOCK/BMAL1-dependent DBP expression is co-activated by circadian MLL1-dependant histone H3 K4 trimethylation^5^, disruption of the MLL1/WDR5 complex by Inh-5 would reduce circadian DBP expression and thereby reduce *Ror- and* ultimately *Bmal1* activation (Supplementary Fig. 12). However, disrupting the MLL1/WDR5 complex should also reduce CLOCK/BMAL1 dependent *Rev-erb* expression and thereby enhance *Bmal1* expression, but this effect may be overcompensated by the reduced DBP/ROR dependent *Bmal1* activation. Furthermore, it is conceivable that these proposed effects of Inh-5 on the MLL1/WDR5 complex, and perhaps even effects on other yet unknown WDR5 WIN site ligands, also occur in wildtype MDF cells, but are overcompensated by the much more drastic *Bmal1* activating effects of Inh-5 on the PER1/WDR5 complex.

### WDR5 WIN site inhibitors mainly affect the circadian clock via the PER1/WDR5 complex

Given that i) Inh-5 treatment results in a much stronger *Bmal1* activation in wildtype MDF cells compared to the *Bmal1* repression observed in *Per1*^−/−^ MDF cells, ii) the negative effect of Inh-5 on *Per2* expression is eliminated in *Per1*^−/−^ MDF cells, and iii) Inh-5 effects that are lost in *Per1*^*−/−*^ MDF cells can be rescued by wildtype PER1 but not by PER1 with a non-functional WIN site, we conclude that Inh-5 mainly affects the circadian clock by weakening the WIN site mediated PER1/WDR5 complex. Hence, our study has identified the WIN site interface of the PER1/WDR5 complex as a main target of WDR5 WIN site inhibitors, that affect circadian regulation of clock genes in the primary and secondary gene regulatory feedback loop. Thereby, our results might become relevant for the development of WDR5 WIN site inhibitors for therapeutic applications in the treatment of clock-related disorders^44^.

## Methods

### Cloning, recombinant expression and purification of PER, WDR5, CRY, MLL1 and RbBP5 proteins

cDNA constructs encoding mouse PER1, PER2, WDR5 and human (h) RbBP5 and MLL1 fragments were subcloned into pEC3CKan^19^, pCoofy3/4^45^ or pGEX6P1 *E.coli* expression vectors. Mouse CRY1/2 cDNA was subcloned into the pCoofy27^45^ insect cell expression vector (primer list: Supplementary Table 3).

PER1, PER2, WDR5, RbBP5, and MLL1 proteins were expressed overnight at 18 °C in *E. coli* Rosetta (DE3) cells using Terrific Broth (TB) medium. Cells were harvested by centrifugation at 4750 × *g* and resuspended in lysis buffer. Cells were lysed with a Branson® sonifier and subsequently cleared by centrifugation at 50,000 × *g* for 45 min, before loading the supernatant to the respective affinity column.

Cells expressing mouse His-GST-WDR5 22-334 (pCoofy3) and mouse GST-PER1 1013-1291 (pGEX6P1) were lysed in 50 mM Tris pH 8.0, 0.2 M NaCl, 5% glycerol, 14 mM β-mercaptoethanol (ß-ME). *Serratia marcescens* (Sm) nuclease was added to cut remaining DNA/RNA. After centrifugation (45 min at 50,000 × *g*), the supernatant was loaded on a self-packed GSH-affinity column, which was washed with at least 5 column volumes (CV) of lysis buffer. The bound GST fusion protein was cleaved on column overnight by adding 3C-PreScission protease and 7.6 mM MgCl_2_. The cleaved protein was eluted with lysis buffer, concentrated, and subjected to size exclusion chromatography (SEC).

Cells expressing mouse His-PER2 1132-1252 (pEC3CKan)^19^ and full-length His-MBP-hRbBP5 1-538 (human RbBP5, Isoform 2, pCoofy4) were lysed in a lysis buffer containing 50 mM Tris pH 8.0, 0.2 M NaCl, 5% Glycerol, 20 mM imidazole, 10 mM β-ME and Sm nuclease. Affinity purification was carried out using a 5 mL His-Trap column (Cytiva) with an elution gradient from 20 mM to 500 mM imidazole. The eluted protein was cleaved overnight with 3C-PreScission protease (His-MBP-hRbBP5) or directly used for SEC (His-PER2). Affinity purified PER1/2, RbBP5, and WDR5 proteins were run on a Superdex S200 16/60 or S200 10/300 SEC column (Cytiva) with a buffer containing 25 mM HEPES pH 7.5, 150 mM NaCl, 5% glycerol, and 2 mM DTT. We note that full-length human RbBP5 only differs from mouse RbBP5 in five positions distant from the WDR5 interacting WBM motif. The usage of human RbBP5 is therefore unlikely to affect our WDR5 interaction studies.

Cells expressing His-GST-hMLL1 3748-3969 containing the WDR5 binding WIN motif and adjacent catalytic SET domain of human MLL1 (pCoofy3) were lysed in 50 mM Tris pH 7.3, 300 mM NaCl, 10% Glycerol, 10 mM ß-ME supplemented with 10 mg Lysozyme, 20 mg Sodium-Desoxycholate, 1 mM PMSF, 5 mM MgCl_2_ and Sm nuclease, and centrifuged for 45 min at 50,000 × *g*. The supernatant was loaded on a self-packed GSH affinity column. The column was washed with 5 CV wash buffer (50 mM Tris pH 7.3, 300 mM NaCl, 10% Glycerol, 10 mM ß-ME), then high salt buffer (wash buffer with 1 M NaCl), then 2 CV wash buffer, before loading 3C-PreScission protease (molar ratio of 1:100) to the column for overnight on-column cleavage. Cleaved hMLL1 3748-3969 protein was eluted with wash buffer. The protein was concentrated and applied to a Superdex S200 16/60 column (Cytiva) with a buffer containing 50 mM Tris pH 7.5, 150 mM NaCl, 5% glycerol, and 2 mM DTT.

His-tagged mouse CRY2-PHR (1-512) and CRY1-PHR (1-496) (pCoofy27) proteins were recombinantly expressed in *Sf9* insect cells (Thermofisher Scientific, #11496015) using the baculovirus expression system. The cells were harvested by centrifugation at 500 × *g* for 10 min and resuspended in lysis buffer (50 mM Tris pH 7.5, 300 mM NaCl, 10% glycerol, 10 mM β-ME, 20 mM imidazole, supplemented with 1 tablet Protease Inhibitor Cocktail (Roche), 5 mM MgCl_2_, and Sm nuclease), followed by mechanical lysis with a Branson® sonifier. The lysate was centrifuged at 50,000 × *g* for 45 min. The supernatant was applied to a His-Trap Ni-affinity column (Cytiva). The His-CRY proteins were eluted with a gradient from 20 mM to 500 mM imidazole. CRY containing fractions were dialyzed adding His-tagged HRV 3C protease in a 1:100 molar ratio. The protease and cleaved His-Tag were removed by reverse Ni-affinity chromatography. If cation exchange chromatography was additionally required, the protein was diluted to 30 mM NaCl with No-Salt buffer (50 mM Tris-HCl pH 7.5, 10% Glycerol, 5 mM β-Mercaptoethanol), loaded onto a HiTrap SP HP cation exchange column (Cytiva) and eluted with a gradient from 30 mM to 1 M NaCl. The tag-free CRY1/2-PHR protein was further purified via SEC using a Superdex S200 10/300 column (Cytiva) with a buffer containing 25 mM HEPES pH 7.5, 150 mM NaCl, 5% Glycerol, and 2 mM DTT.

### Analytical Size exclusion chromatography of PER/WDR5-, PER/CRY-, WDR5/RbBP5 and PER/WDR5/RbBP5 complexes

For complex formation, 1:3 ratios (His-PER2 1132-1252, PER1 1013-1291 + PER2 1132-1252 or hRbBP5 with 3-fold excess of WDR5 22-334) or equimolar ratios (PER1/2 with CRY1 1-496; PER1 with WDR5; PER1/2 with WDR5 and hRbBP5) of the wildtype or mutant proteins were mixed and incubated overnight on ice. For His-PER2 complexes, 3C-protease was added for the overnight incubation to remove the His-Tag of PER2. The proteins were concentrated to 500 µL and run on a Superdex S200 10/300 or S75 10/300 SEC column (Cytiva) with a buffer containing 25 mM HEPES pH 7.5, 150 mM NaCl, 5% glycerol, and 2 mM DTT. The individual proteins were run on the same column as a reference. SEC chromatograms were collected with ChromLab v.6.1 and analyzed with the Origin® version v7.5 and the OriginPro version 2023 (OriginLab Corporation, Northampton, MA, USA) software. Protein fractions were analyzed by SDS-PAGE.

### Analytical Size exclusion chromatography of PER/WDR5/CRY- and MLL1/WDR5 complexes

For complex formation, proteins were mixed in an equimolar ratio at a final concentration of 90 µM or 100 µM. For PER/CRY/WDR5 complex formation, PER (PER1 1013-1291, PER2 1132-1252), CRY (CRY1 1-496, CRY2 1-512), and WDR5 22-334 proteins were mixed in equimolar ratios, incubated for 60 min at 4 °C, and then injected onto an analytical S200 10/300 SEC column. Alternatively, the PER/WDR5 complex was pre-formed by overnight incubation at 4 °C, and then equimolar amounts of CRY were added and incubated for 60 min at 4 °C before loading onto an analytical S200 10/300 SEC column. To generate an equimolar MLL1/WDR5 complex, we incubated an equimolar mixture of MLL1 (hMLL1 3748-3969, WIN-SET) and WDR5 22-334 proteins at 4 °C for 2 h, ran an S200 10/300 SEC column, and pooled only SEC fractions containing the equimolar MLL1/WDR5 complex. Afterwards, equimolar amounts of PER1 were added to the preformed MLL1/WDR5 complex and incubated for 60 min at 4 °C before loading the mixture onto an analytical S200 10/300 SEC column. The SEC running buffer consisted of 25 mM HEPES pH 7.5, 300 mM NaCl, 5% glycerol, and 1 mM DDT. Protein fractions were analyzed by SDS-PAGE. SEC chromatograms were collected with ChromLab v.6.1, analyzed in Microsoft 365® Excel, and plotted with GraphPad Prism 9.5.1.

### Analytical size exclusion chromatography with WIN site inhibitors

WDR5 22-334 and PER1 1013-1291 or WDR5 22-334 and hMLL1 3748-3969 (WIN-SET) were mixed in equimolar ratios for a final concentration of 120 µM and incubated on ice overnight (PER1 + WDR5) or for 2 h (hMLL1 + WDR5). Inhibitors Inh-4 (MCE, HY-111753) and Inh-5 (MCE, HY-150654) were dissolved at high concentrations in 100 % DMSO and added to the preformed protein complexes. By dilution the final concentrations of the protein complex and the inhibitor decreased to 60 µM and 0.06 % DMSO. The protein complexes with added inhibitors were incubated for 1 h on ice before loading 100 µl onto a SEC S200 10/300 column. The SEC buffer contained 25 mM HEPES pH 7.5, 150 mM NaCl, 5% glycerol, and 2 mM DDT. Protein fractions were analyzed by SDS-PAGE. Chromatograms were collected with ChromLab v.6.1, analyzed in Microsoft 365® Excel, and plotted with GraphPad Prism 9.5.1.

### Site-directed mutagenesis

PER1, PER2 and WDR5 mutants were generated by QuikChange site-directed mutagenesis and verified by sequencing (Mutagenesis primers: Supplementary Table 3). The mutant constructs were expressed and purified to homogeneity essentially as described for the wild-type proteins.

### Crystallization

Crystals of the WDR5 22-334 N225A protein (10 mg/mL) were obtained at 20 °C using a hanging drop setup with a reservoir solution containing 0.1 M HEPES pH 7.5, 50 mM (NH_4_)_2_SO_4_, and 24% PEG 3350. Crystals belong to space group C222_1_ with 1 molecule per asymmetric unit and cell constants *a* = 78.5 Å, *b* = 98.13 Å, *c* = 80.06 Å. The crystals were shock frozen in liquid nitrogen using a cryoprotective solution with 15% PEG 200.

WDR5 22-334 was co-crystallized with a synthetic peptide (Genscript) including residues 1198–1211 of mouse PER2 and an N-terminal tyrosine for absorbance at 280 nm (PER2 Y1198-1211). Crystals of the WDR5/PER2 Y1198-1211 complex (12 mg/mL WDR5, 2 mM PER2 peptide) were obtained at 20 °C using a hanging drop setup with a reservoir solution containing 0.1 M Tris pH 8.0, 50 mM NaCl and 13% PEG 8000. Crystals belong to space group P4_2_ 2_1_ 2 with unit cell constants *a* = *b* = 78.15 Å, *c* = 101.75 Å and 1 complex per asymmetric unit. The crystals were shock frozen in liquid nitrogen with a cryoprotective solution containing 20% PEG 200.

### Data collection, structure determination, and refinement

A 1.38 Å data set was collected of a WDR5 N225A crystal at SLS X06SA (Villigen, Switzerland) and a 1.85 Å data set of a WDR5/PER2Y1198-1211 complex crystal at DESY/EMBL PX13 (Hamburg, Germany). All data sets were processed using XDS (Versions 2017/2020)^46^ and evaluated using the half set correlation in the highest resolution shell. The data were scaled with AIMLESS (CCP4i2)^47^, and a 5% R_free_ dataset was created. All structures were solved using PHASER (CCP4i2)^47,48^ for molecular replacement with Apo-WDR5 (PDB 2H9L) as search model. Model building and refinement were performed with Coot (0.9.8.95)^49^ and REFMAC (CCP4i2)^47,50^. The models of WDR5 N225A and WDR5/PER2Y1198-1211 show very good stereochemistry and *R*_work_/*R*_free_-values of 0.194/0.218 (WDR5 N225A) and 0.1651/0.2007 (WDR5/PER2Y1198-1211) (Supplementary Table 1).

The WDR5/PER2 Y1198-1211 structure consists of 314 amino acids, where WDR5 23-29 and PER2 Y1198-1202 were not visible in the electron density due to conformational disorder. Additionally, the model includes 98 water molecules, 2 Tris molecules, 1 Polyethylenglycol molecule, and 2 Chloride ions. PER2 T1203 was modeled as alanine due to conformational disorder of the side chain. The Ramachandran plot shows 94.19% of the main chain torsion angles in the most favored regions and no residues marked as outliers.

The WDR5 22-334N225A structure consists of 301 amino acids, where residues 23–31 and 334 were not visible in the electron density, and K227 and R181 (near A225) were modeled as alanine due to conformational disorder. 245 waters are included in the model. The Ramachandran plot shows 94.33% of the main chain torsion angles in the most favored regions and 0 amino acids marked as outliers. All figures were generated with PyMOL (0.99rc6)^51^.

### Isothermal titration calorimetry

To determine the K_D_ values for the interactions of PER2 1132-1252 and PER1 1013-1291 with either WDR5 22-334 or CRY1 1-496, and of RbBP5 with WDR5, ITC experiments were performed and analysed using a MicroCal PEAQ-ITC 1.41 Automated system (Malvern Panalytical, Malvern, UK). All used proteins were dialyzed for at least 16 h to a buffer containing 25 mM HEPES pH 7.5, 150 mM NaCl, 5% glycerol, and 1 mM β-ME. A 750 µM solution of WDR5 (WT or N225A, syringe) was titrated to 50 µM solutions of PER1 1013-1291 (WT, R1111A, D1197A, R1111A D1197A, R1111A R1113A), PER2 1132-1252 (WT, I1205G, D1206A, V1207A) or RbBP5 (cell). For the PER-CRY1 interaction, 90 µM solutions of PER1 1013-1291 (WT and mutants) or PER2 1132-1252 (WT and mutants) (syringe) were titrated into an 8 µM CRY1 1-496 solution (cell). The titration consisted of 19 injections of 2 µl every 150 s at 25 °C. As a control, the protein in the syringe was titrated into a cell filled with buffer. The analysis of the data was performed with the Microcal PEAQ-ITC Analysis software, using a one-site binding model. All experiments were performed in triplicate using three distinct samples, and a mean of the individual measurements was used for further interpretation.

### Gateway cloning for BRET

The donor and acceptor vectors pcDNA3.1-cmyc-NL-GOI (Addgene plasmid ID #113446), pcDNA3.1-GOI-NL-cmyc (Addgene plasmid ID #113447), pcDNA3.1, pcDNA3.1-cmyc-NL (Addgene plasmid ID #113442), and pcDNA3.1-PA-mCit (Addgene plasmid ID #113443) were kindly provided by the Wanker Group (Max-Delbrück-Centrum für Molekulare Medizin, Berlin, Germany). Plasmids pcDNA3.1-GOI-His3C-mCit and pcDNA3.1-mCit-His3C-GOI were generated by introducing a Protease 3C cleavage site between the GATEWAY cassette and mCitrine, starting off from plasmids pcDNA3.1-mCit-GOI and pcDNA3.1-GOI-mCit provided by the Wanker lab. Mouse WDR5 22-334, PER1 1-1291 (FL), and PER1 1-1291 R1111A R1113A were cloned into pDONR223 following Gateway BP-cloning protocols from ThermoFisher. Subsequently, they were cloned into pcDNA3.1-cmyc-NL-GOI and pcDNA3.1-mCit-His3C-GOI following the Gateway LR-cloning protocols from ThermoFisher. (primers: Supplementary Table 3)

### Cell treatment for BRET assay

HEK293 cells (DSMZ, ACC305) were grown and maintained in high-glucose (4.5 g/l) DMEM (Thermo Fisher) for Bioluminescence Resonance Energy transfer (BRET) assays. Media were supplemented with 10% fetal bovine serum (Thermo Fisher) and 1% Penicillin/Streptomycin. Cells were grown at 37 °C, 5% CO_2_, and 85% relative humidity (RH). Cells were regularly tested for mycoplasma contamination. Cells were subcultured every 2–3 days and transfected with 0.5 µl lipofectamine 2000 transfection reagent (Invitrogen) per well in Opti-MEM medium (Thermo Fisher) using the reverse transfection method according to the manufacturer’s instructions. For transfections, cells were seeded at a density of 4.0 × 104 cells per well in a white 96-well microtiter plate (Greiner) in phenol-red-free, high-glucose DMEM media (Thermo Fisher) supplemented with 5% fetal bovine serum (Thermo Fisher) and 1% Penicillin/Streptomycin.

### Donor saturation BRET assay

For donor saturation experiments, various donor DNA amounts (1, 2, 4, and 8 ng) encoding Nanoluciferase (NL)-fused proteins were co-transfected with increasing amounts of acceptor DNA (12.5, 25, 50, 100, and 200 ng) encoding mCitrine-fused proteins. Transfections were performed with a total DNA amount of 200 ng per well. If the expression plasmid concentration amount was below 200 ng/well, pcDNA3.1 (+) was used as a carrier DNA to reach the total amount of DNA of 200 ng.

The plate with transfected cells was incubated for 2 days at 37 °C, 5% CO_2_, and 85% RH before measurements. All measurements were made with the Tecan Infinite 200 Pro microplate reader (Software: Tecan i-control, version 3.40_01/15_Infinite). First, 100 µl of the medium was aspirated from each well. The mCitrine fluorescence (FL) was measured in intact cells (excitation/emission 513 nm/548 nm) using a gain of 100 with four measurements per well that were averaged to estimate expression levels of mCit fusion proteins. After measuring the fluorescence, coelenterazine-h (PJK Biotech GmbH) was added to a final concentration of 5 µM. The cells were briefly shaken for 15 s and incubated for 15 min inside the plate reader at 37 °C. After incubation, total luminescence was measured over an integration time of 1 s to estimate expression levels of NL fusion proteins. Subsequently, the BRET was determined by measuring over a short-wavelength (WL) and long-wavelength range the luminescence using the dual filters BLUE1 (370–480 nm) and the GREEN1 (520–570 nm) at 1000 ms integration time. The BRET is the ratio of luminescence from the long over the short wavelength measurement.

Normalized BRET ratios were calculated by subtracting the BRET from cells transfected with pcDNA3.1-cmyc-NL at 2 ng to remove signal from bleedthrough. Fluorescence and total luminescence measurements were corrected for background signal using transfections with pcDNA3.1(+) and subsequently used to estimate amounts of expressed proteins and to plot acceptor/donor ratios on the x-axis of titration plots. We used GraphPad Prism 9.5.1 and the one-site specific binding model for curve fitting. From the fitted models, the BRET50 and BRETmax can be directly extracted.

### Immunocytochemistry

Transfections were performed with the jetPRIME Kit (Polyplus, Illkirch, France) according to protocol: For immunofluorescence analysis, 1.5 × 10^5^ cells were seeded into one well of a 6-well plate and transfected with 1.5 µg plasmid DNA the following day. For immunocytochemical staining, medium was removed from HEK293T cells (ATTC, ACS-4500) 24 h post transfection. Cells were washed once with PBS. Cells were fixed by incubation with 4% paraformaldehyde for 10 min at room temperature. Cells were washed three times with 1x PBS. Formaldehyde was quenched by addition of 50 mM NH4Cl for 10 min at room temperature. Coverslips were incubated for 15 min with PBS-TX (0.3% v/v Triton X-; in PBS) for permeabilisation. For blocking, cells were incubated in Fish Blocking Buffer TX (0.1% w/v Ovalbumin; 0.5% w/v Gelatin from coldwater fish; 0.3% v/v Triton X-; in PBS) for 1 h. Monoclonal (clone 9B11) mouse anti-Myc-Tag antibody (Cell Signaling Technology Cat# 2276; Lot # 24; RRID: AB_331783, 1:5000 dilution) in Fish Blocking Buffer TX was added to the cells and incubated overnight at 4 °C under high humidity. Cells were washed three times with PBS-TX. Alexa Fluor Plus 555 Donkey anti mouse 1:400 (Invitrogen Cat# A31570) and 40,6-Diamidino-2-phenylindol (DAPI, 1:8000; both in Fish Blocking Buffer TX) were added to the cells. After 1 h incubation, cells were washed twice with PBS-TX and once with PBS. Cells were mounted in Fluoromount-G (SouthernBiotech, Birmingham, AL) on a SuperFrost slide and curated for 24 h before imaging. Images of immunocytochemically stained cells were acquired on a Leica DM6000B microscope (Leica Microsystems GmbH, Wetzlar, Germany) with a sCMOS Microscope Camera K5 (Leica Microsystems GmbH, Wetzlar, Germany). The software used for image visualization was Leica Application Suite X (LAS X) (Leica Microsystems GmbH, Wetzlar, Germany). Fluorescence images were processed in Fiji80 with the LIF2TIF image converter. The script to prepare and analyse image micrographs is taken from^52^. Line plots were created using the deposited Macro image preparation protocol (https://doi.org/10.6084/m9.figshare.22140278.v1).

### Bmal1::Luc reporter assay in U-2 OS cells

U-2 OS cells (human, ATCC # HTB-96) stably expressing firefly luciferase from a 0.9-kb *Bmal1* promoter fragment^14^ were generated by lentiviral transduction of the respective expression plasmid. A single subclone was used for further experiments^53^. Reporter cells were cultured essentially as described^54^ in phenol-red-free DMEM medium containing 10% fetal calf serum, antibiotics, and 250 µM D-luciferin (Biothema). Cells were synchronized by incubation with 1 µM dexamethasone (Sigma-Aldrich, #D4902) for 20–30 min under cell tissue culture conditions. Cells were then washed twice with PBS incubated with various concentrations of WDR5-IN-4 TFA (MedChemExpress) or solvent (0.5% DMSO). Bioluminescence recordings were performed at 35–37 °C in 96-well plate luminometers (TopCount, PerkinElmer). Bioluminescence data were analyzed with the ChronoStar software as described^55^.

### Isolation of mouse dermal fibroblasts

Mouse dermal fibroblasts (MDF cells) were obtained from deceased heterozygous *Per2::*Luc mice^35^ or the same mice crossed with *Per1*^−/−^ mice derived from Per1LoxP/LoxP mice that were crossed with a total deleter strain expressing Cre recombinase^36^ to obtain heterozygous *Per2::*Luc mice, according to an established protocol for human skin fibroblasts^56^. Briefly, the ears were chopped into small pieces, slightly digested with Liberase (Roche, Basel, Switzerland), and then left in DMEM medium with 20% fetal calf serum and 100 units/mL of penicillin, 100 μg/mL of streptomycin, 25 μg/mL amphotericin and 100 μg/mL gentamycin (culture medium) until the dermal fibroblasts crawled out from the tissue and attached to the plastic. After two more passages, the dermal fibroblast cultures, when diluted 1–3 were growing to confluency within three days. Early passages (around passage P4) were frozen regularly at −70 °C, because we found that with increasing passage number the bioluminescence of the PER2::Luc reporter somewhat declined (late passages, *P* > 20). Cultures of immortalized cells, indicated by a smaller size and loss of contact inhibition, were discarded because they lost most of their circadian properties. We successfully established cultures from heterozygous *Per2::*Luc mice (referred to as W for wild-type) and *Per1*^−/−^ x heterozygous *Per2*::Luc mice (referred to as M for mutant, i.e., the *Per1*^−/−^ knock out).

### Lentiviral vector construction

The plasmid pLenti6 (Invitrogen) was modified by inserting an *EcoRI*x*SalI*-linker into the original *ClaI* and *AgeI* restriction sites of the vector to remove a 2375 bp insert. A *EcoRI*x*SalI* fragment containing the mouse *Per1* open reading frame with 3 V5 tags^43^ was obtained by PCR. The original plasmid was also mutagenized to convert arginine 1111 and 1113 to alanine (R1111A, R1113A) by introducing a *NotI* restriction site. The mutated plasmid was then used as another template for PCR. Either of these fragments was then cloned into the *EcoRI* and *SalI* restriction sites of the modified vector. A 5748 bp genomic fragment of *Per1* spanning promoters 1 and 2^37^ was flanked by PCR with *EcoRI* restriction sites and cloned into the singular *EcoRI* restriction site of the modified vector with the two different Per1V5 inserts. The correct orientation of the genomic fragment was screened by digestion with *SphI*. The final lentiviral vectors were referred to as pLenti6xgPer1V5 (wild-type PER1) and pLenti6xgPer1V5mut (PER1 R1111A R1113A mutant).

The open reading frame of mouse *Wdr5* was obtained by PCR from reverse-transcribed cDNA starting from mouse dermal fibroblast RNA and flanked with *Nhe1* and *SalI* restriction sites. This fragment was cloned into the corresponding sites within the vector pCW57.1 (Addgene plasmid#41393 from David Root). Primers for cloning and mutagenesis are listed in Supplementary Table 5.

Production of lentiviral particles was done according to^56^. Briefly, 3 μg of pMD2G, 8 μg psPAX2, and 10 μg of either lentiviral vector in 500 μL 150 mM NaCl were mixed with 500 μL of 40 μg/mL Lipofectamine 2000 in 150 mM NaCl, incubated 20 min at room temperature, and used to transfect a 10 cm dish with semi-confluent 293 T cells. Viral vector-containing supernatant was harvested after one and two days. All supernatants from two such transfections were pooled and concentrated by centrifugation at 100,000 × *g* for 2 h. The viral vector pellets were resuspended in 3 mL culture medium and stored as 500 µL aliquots at −70 °C until use.

### Generation of stable mouse dermal fibroblast cell lines

To infect *Per1*^−/−^x *Per2*::Luc mouse dermal fibroblasts, 10 mL of culture medium supplemented with 8 μg/mL protamine sulfate and 500 µL of concentrated lentiviral vector supernatant was applied through a 0.45 PVDF filter directly on the previously emptied culture dish with about 30% confluent cells. After an over-night incubation, the medium was replaced by fresh medium. After a total of three days, the culture was splitted into 2 new 10 cm dishes containing culture medium supplemented with 15 μg/mL blasticidin for pLenti6-based constructs, or 10 μg/mL puromycin for the pCW57.1-based construct. This selection was continued until the surviving cells started to reproduce normally and grew to confluency. To maintain transgene expression, 10 μg/mL blasticidin or 7 μg/mL puromycin was given to the culture medium. We were able to generate rescue cell lines with either the genomic Per1V5 or the genomic *Per1*V5mut. Cells with stable integration of the pCW57.1 x Wdr5 construct (referred to as WT), allowing the doxycycline-depended expression of WDR5, were grown in culture medium with charcoal-stripped fetal calf serum. To induce the expression of the transgene, we added 1 μM doxycycline to the medium.

### Measurement of cellular circadian rhythms using the LumiCycler apparatus

Cells were diluted into 3.5 cm dishes and grown to confluency in culture medium with selective antibiotics. The medium was replaced by DMEM without phenol red supplemented with 2% fetal calf serum, 1% GlutaMax, 1% sodium pyruvate, 100 units/mL of penicillin, 100 μg/mL of streptomycin, 25 μg/mL amphotericin, 100 μg/mL gentamycin, 25 mM HEPES, 100 nM D-luciferin, and 10 µM forskolin (lumicycler medium). The plates were sealed with Parafilm and placed into the LumiCycle apparatus, which holds 32 samples but can measure only four samples at the same time. All samples were processed as quadruplicates using either of the four photomultiplier tubes. The experiment was stopped after 3.5–4 days, when the cells were still alive. The inhibitors 4 and 5 were prepared as stock solutions of 10 mM in DMSO and stored as 10 μL aliquots at −70 °C until use. 10-fold dilutions were obtained starting from 10 μM by adding 1 volume of the highest concentration to 9 volumes of Lumicycler medium with 0.1% DMSO and then continued to obtain the other dilutions. The raw data from the experiment were downloaded into an Excel file. The data obtained from photomultiplier A were scaled by a factor of 1.45, for B 1.25, and for D 1.5 to match the efficiency of photomultiplier C. The synchronization of the luciferase reporter gene with forskolin provoked a drastic increase in bioluminescence for the first 6–10 h. Hence, for better presentation, we excluded the first 12 h of the measurement. We used these normalized raw counts to display the effect of the inhibitors on the reporter gene activity. To calculate amplitude and period length differences, we obtained base-line subtracted data with the software LumiCycle Analysis from Actimetrics (version 2.5). These data were not scaled. Briefly, we used the “Sin fit (damped)” function to determine the period length and amplitude and to calculate the optimal curve fit using the first and second peaks of either curve. The baseline was adjusted according to a polynom of *n*th-order to achieve the best goodness of fit.

### Measurement of cellular circadian rhythms using total RNA

The cells were grown in either 3.5 cm plates or 6-well plates until reaching confluency. The cells were synchronized with Lumicycle medium and harvested at the indicated time points. Briefly, the plate was washed twice with cold PBS, and then 300 μL Trizol added. The cells were then scrapped of the plate and transferred to 1.5 mL reaction tubes. 30 μL of 1-Bromo-3-chloropropane was added, mixed, and the phases let separate for 5 min at room temperature (RT). After a centrifugation of 10 min at 4 °C, 12’000 × *g*, about 180 μL of the liquid phase was loaded into prefilter columns of the NucleoSpin RNA-XS kit (Macherey & Nagel). The purification of total RNA was then proceeded according to the instructions of the kit. 0.5 μg of total RNA was reverse transcribed using the Superscript VILO-cDNA kit (Invitrogen) according to the instruction of the kit. Specific transcripts were detected using real-time PCR with KAPA fast universal 2x master mix and the indicated probes. Quantification of mRNA was carried out using TaqMan (TM) hydrolysis probes with a FAM (6-fluorescin) fluorophore attached to the 5’ end and the black hole quencher 1 (BHQ1) attached to the 3’ end of the oligonucleotide probes (Supplementary Table 4). A Rotorgene RG-6000 machine was used to measure FAM fluorescence (*λ*_ex_: 470 nm, *λ*_em_: 510 nm). The geometric mean of four non-circadian genes (Nono, Sirt2, Cdk5, Atp5h) was used for normalization of the samples. To measure mRNA, we designed primers/TaqMan probe to span exon-exon boundaries within a transcript, while for pRNA (unspliced precursor RNA) we designed the primers/TaqMan probe to span intron-exon boundaries (Supplementary Table 4). Contaminating genomic DNA contributed to less than 10% of the obtained signal for the minimal value.

Two-way ANOVA was employed to determine *P*-values for treatment (effect of WDR5 overexpression (Supplementary Fig. 8), inhibitor 5 (Inh-5; Fig. 6 and Supplementary Fig. 9) or inhibitor 4 (Inh-4; Supplementary Fig. 11) and for time (day-time/circadian variation). Additionally, we employed two-way ANOVA to determine if the effect of WDR5 overexpression (Supplementary Fig. 8), or the treatment with inhibitor 5 (Fig. 6 and Supplementary Fig. 9) and inhibitor 4 (Supplementary Fig. 11) was independent of daytime or not (non-significant or significant interaction, respectively). In case of a non-significant interaction, the effect of the treatment on the RNA accumulation can be considered. Note that the daytime in most cases highly-significantly affected the RNA accumulation because we investigated genes expressed in a diurnal way.

### Statistical analysis

GraphPad Prism 9.5.1 was used for data presentation and statistical analysis (GraphPad Software Inc., California, USA). A Student’s two-tailed *t*-test, an ordinary one-way ANOVA, a Dunnett’s multiple comparison test, a multiple *t*-test or a two-way ANOVA were used when appropriate and as described in figure legends, where *p ≤ 0.05, **p < 0.01, ***p < 0.001, ns≥0.05. Data are shown as means ± S.E.M. unless otherwise specified.

### Reporting summary

Further information on research design is available in the Nature Portfolio Reporting Summary linked to this article.

## Supplementary information

Supplementary Information

Reporting Summary

Transparent Peer Review file

## Source data

Source Data

## Data Availability

Coordinates for the crystal structures of WDR5 N225A (PDB ID 8OK1, https://doi.org/10.2210/pdb8OK1/pdb) and the WDR5/PER2 complex (PDB ID 8OKF, https://doi.org/10.2210/pdb8OKF/pdb) were deposited in the wwPDB database. All other data are deposited in Zenodo, “Lab_Repo_Eva_Wolf”, https://doi.org/10.5281/zenodo.17224236. Source data are provided with this paper.
