# Supplementary material for: Role of PER-WDR5 interactions in the mammalian circadian clock
Source: Nat Commun. 2026 Sep 8;17:10627. doi: 10.1038/s41467-026-77583-0 (PMC13649712; doi:10.1038/s41467-026-77583-0)
Supplement: Supplementary file 1 [file 41467_2026_77583_MOESM1_ESM.pdf]

Role of PER-WDR5 interactions in the mammalian circadian clock

Arne Börgel[1,*], Florian Hof[1,*], Mareen Welzel[2], Audrey Rawleigh[3], Marcel Wedemann[1,7], Rafaela Mororo Cavalcanti Keller[4], Vanessa Maissl[1], Kyra Ricci[3], Sandra Schick[5], Helen L. May-Simera[1], Steven Brown[3], Achim Kramer[4], Katja Luck[2], Jürgen A. Ripperger[6,#] and Eva Wolf[1,#]

[1]Institute of Molecular Physiology (IMP), Johannes Gutenberg-University Mainz, 55128 Mainz, Germany

[2]Integrative Systems Biology Group, Institute of Molecular Biology (IMB), 55128 Mainz, Germany

[3]Chronobiology and Sleep Research Group, Institute of Pharmacology and Toxicology, University of Zürich, 8057 Zürich, Switzerland

[4]Laboratory of Chronobiology, Charité-Universitätsmedizin Berlin, 10117 Berlin, Germany

[5]Chromatin Regulation Group, Institute of Molecular Biology (IMB), 55128 Mainz, Germany

[6]Department of Biology, University of Fribourg, CH-1700 Fribourg, Switzerland

[7]Current address: Technische Hochschule Bingen, 55411 Bingen am Rhein, Germany

* These authors contributed equally to the work

# Correspondence should be addressed to Eva Wolf (evawolf1@uni-mainz.de) or Jürgen Ripperger (juergenalexandereduard.ripperger@unifr.ch)

Supplementary Information

Supplementary Table 1: X-ray data collection and refinement statistics of WDR5 N225A and WDR5/PER2 Y1198-1211 complex crystal structures.

	**WDR5 22-334 N225A**	**WDR5 22-334/PER2 Y1198-1211**
Data collection		
**Beamline**	SLS X06SA	DESY PX13
**Wavelength (Å)**	0.9999	0.9763
**Resolution range (Å)**	48.67–1.38 (1.40–1.38)*	78.15-1.85 (1.89–1.85)*
**Space group**	C 2 2 21	P 42 21 2
**a, b, c (Å)**	78.498, 98.134, 80.055	78.152, 78.152,101.748
**α, β, γ (°)**	90, 90, 90	90, 90, 90
**Unique reflections**	63526 (2949)*	27636 (1653)*
**Multiplicity**	1.9 (2.0)*	14.2 (14.4)*
**Completeness (%)**	99.3 (93.1)*	100 (100)*
**Mean I/σ(I)**	13.0 (1.2)*	18.3 (5.2)*
**$CC_{1/2}$**	0.999 (0.573)*	0.999 (0.955)*
**R_{merge}**	0.028 (0.291)*	0.103 (0.541)*
Refinement		
**Protein atoms**	2349	2406
**Water molecules**	245	98
**R_{work}**	0.1944	0.1651
**R_{free}**	0.2177	0.2007
**RMSD bonds (Å)**	0.013	0.012
**RMSD angles (°)**	1.802	1.647
**B-factors overall**	19.43	20.15
**B-factors**	18.26 (WDR5), 30.74 (H_2O)	19.53 (WDR5), 32.13 (PER2), 23.94 (H_2O)
**Ramachandran**		
**Favored (%)**	94.33	94.19
**Allowed (%)**	5.67	5.81
**Outliers (%)**	0	0

* Numbers in brackets refer to highest resolution shells.

Supplementary Table 2: ITC results of PER1 1013-1291, PER2 1132-1252, WDR5 22-334, CRY1 1-496 and RbBP5 (WT and mutants) protein interactions. Average values of three measurements with standard deviations are shown.

Cell / Analyte	Syringe / Titrant	N (sites)	K_D (µM)	ΔH (kJ/mol)	ΔG (kJ/mol)	-TΔS (kJ/mol)
**PER1** WT	**WDR5** WT	0.8 ± 0.003	3.09 ± 0.20	-46.13 ± 0.62	-31.5 ± 0.17	14.70 ± 0.77
PER1 R1111A	WDR5 WT	1.0 ± 0.01	13.47 ± 1.48	-36.80 ± 1.58	-27.87 ± 2.99	8.93 ± 1.86
PER1 D1197A	WDR5 WT	1.8 ± 0.007	5.65 ± 0.16	-45.87 ± 0.48	-30.00 ± 0.06	15.9 ± 0.54
PER1 R1111A D1197A	WDR5 WT	0.8 ± 0.03	16.17 ± 1.71	-44.10 ± 3.29	-27.40 ± 0.28	16.67 ± 3.55
PER1 R1111A R1113A	WDR5 WT	Weak binding*	> 100			
PER1 WT	WDR5 N225A	0.8 ± 0.01	7.26 ± 0.24	-35,77 ± 0.61	-29.4 ± 0.096	6.38 ± 0.7
**PER2** WT	**WDR5** WT	0.9 ± 0.1	50.73 ± 19.33	-16.87 ± 5.195	-24.93 ± 0.91	-7.99 ± 6.08
PER2 I1205G	WDR5 WT	Not binding				
PER2 D1206A	WDR5 WT	Not binding				
PER2 V1207A	WDR5 WT	Weak binding*	99.07 +/- 34.41			
PER2 WT	WDR5 N225A	Not binding				
**CRY1**	**PER1** WT	0.8 ± 0.004	0.016 ± 0.006	-87.70 ± 3.23	-44.80 ± 1.02	42.87 ± 4.25
CRY1	PER1 R1111A	0.9 ± 0.02	0.0098 ± 0.0035	-76.27 ± 0.73	-46.03 ± 0.86	30.3 ± 0.48
CRY1	PER1 D1197A	1.0 ± 0.02	0.157 ± 0.036	-66.53 ± 1.64	-38.97 ± 0.53	27.53 ± 2.00
CRY1	PER1 R1111A D1197A	1.0 ± 0.006	0.196 ± 0.032	-63.60 ± 0.71	-38.40 ± 0.415	25.23 ± 1.08
**CRY1**	**PER2** WT	0.9 ± 0.05	0.035 ± 0.013	-54.38 ± 5.41	-43.28 ± 1.15	11.10 ± 6.06
CRY1	PER2 I1205G	0.8 ± 0.01	0.086 ± 0.012	-80.25 ± 2.16	-40.43 ± 0.31	39.83 ± 2.20
CRY1	PER2 D1206A	0.8 ± 0.04	0.069 ± 0.025	-46.93 ± 3.41	-41.48 ± 0.95	5.45 ± 3.86
CRY1	PER2 V1207A	0.9 ± 0.06	0.037 ± 0.013	-63.73 ± 0.75	-48.53 ± 6.70	15.20 ± 7.20
**RbBP5**	**WDR5** WT	1.0 ± 0.003	0.61 ± 0.014	-35.97 ± 0.22	-35.50 ± 0.063	0.48 ± 0.27
RbBP5	WDR5 N225A	Weak binding 1.6 ± 0.1	71.00 ± 18.12	-9.09 ± 1.71	- 23.87 ± 0.67	-14.73 ± 2.31

* N, ΔH, ΔG and -TΔS were omitted due to very weak binding.

Supplementary Table 3: Primers used for cloning and mutagenesis of recombinantly expressed mouse (m) PER1/2, CRY1/2 and WDR5 constructs and human (h) MLL1 and RbBP5 constructs and primers used for BRET Gateway cloning of mPER1 and WDR5.

Name	Sequence (5'-->3')
**mPER2 1132 fw**	CCAGGGGCCCGACTCGATGTACGTCTTGCAGGACCCCATCTGG
**mPER2 1252 rv**	CAGACCGCCACCGACTGCTTACCTGGGGCCTGTCAGCTGTTC
**mPER1 1013 fw**	AAGTTCTGTTCCAGGGGCCCAGTGAGGAGACTGCTGAGCC
**mPER1 1291 rv**	CCCCAGAACATCAGGTTAATGGCGTCAGCTGGTGCTGTTTTCTTCTGCAG
**mCRY2 1 fw**	CTGGAAGTTCTGTTCCAGGGGCCCATGGCGGCGGCTGC
**mCRY2 512 rv**	CCCCAGAACATCAGGTTAATGGCGTCAGTATCTCGACAGCTGTTGGTAG
**mCRY1 1 fw**	AAGTTCTGTTCCAGGGGCCCATGGGCGTCAATGCT
**mCRY1 496 rv**	CCCCAGAACATCAGGTTAATGGCGTCATAACCCGCGATACCTTG
**mWDR5 22 fw**	AAGTTCTGTTCCAGGGGCCCTCAGCCACACAGAGCAAGCCCACA
**mWDR5 334 rv**	CCCCAGAACATCAGGTTAATGGCGTTAGCAGTCACTCTTCCACAGTTT
**hMLL1 3748 fw**	AAGTTCTGTTCCAGGGGCCCCACAAGCCAGAGGAGG
**hMLL1 3969 rv**	CCCCAGAACATCAGGTTAATGGCGTTAGTTTAGGAACTTCCGGCATTTCTTGG
**hRbBP5 fw**	AAGTTCTGTTCCAGGGGCCCATGAACCTCGAGTTGCTG
**hRbBP5 rv**	CCCCAGAACATCAGGTTAATGGCGTTATAACAGTTCTGAGATTGCTCC
**Mut mPer2 I1205G fw**	CCTACGGCCGGCGATGTGACAGGCTGTGTTTAC
**Mut mPer2 I1205G rv**	TGTCACATCGCCGGCCGTAGGCAGGCC
**Mut mPer2 D1206A fw**	CTACGGCCATCGCGGTGACAGGCTGTGTTTACTGC
**Mut mPer2 D1206A rv**	GCCTGTCACCGCGATGGCCGTAGGCAGGCCCCC
**Mut mPer2 V1207A fw**	GCCATCGATGCGACAGGCTGTGTTTACTGCGAG
**Mut mPer2 V1207A rv**	CACAGCCTGTCGCATCGATGGCCGTAGGCAGGCC
**Mut mPer1 R1111A fw**	GGGGCTGCTGCGGCCAGGACTGAGCCTGGGGACCAG
**Mut mPer1 R1111A rv**	CAGTCCTGGCCGCAGCAGCCCCAGCTTCAGCCTCGG
**Mut mPER1 R1111A R1113A fw**	GCTGCTGCGGCCGCGACTGAGCCTGGGGACCAG
**Mut mPER1 R1111A R1113A rv**	CTCAGTCGCGGCCGCAGCAGCCCCAGCTTCAGC
**Mut mPer1 D1197A fw**	CGGGCCCTTGCTGTGATGGCGTGTGTGGACTGTGGC
**Mut mPer1 D1197A rv**	CGCCATCACAGCAAGGGCCCGAGGCAGCTGGCCCTTC
**Mut mWDR5 N225A fw**	TTCTCTCCAGCTGGCAAATACATCCTGGCT
**Mut mWDR5 N225A rv**	GTATTTGCCAGCTGGAGAGAACTTCACGAAG
**mPER1 attB1 -BRET**	GGGGacaagtttGTACAAAAAAGCAGGCTGCATGAGTGGTCCCCTAGAAGG
**mPER1 attB2 - BRET**	GGGGaccactttgtacaagAAAGCTGGGTCCTAGCTGGTGCTGTTTTCTTC
**mWDR5 attB1 - BRET**	GGGGacaagtttGTACAAAAAAGCAGGCTGCTCAGCCACACAGAGCAAGC
**mWDR5 attB2 - BRET**	GGGGaccactttgtacaagAAAGCTGGGTCTTAGCAGTCACTCTTCCACAGT

Supplementary Table 4: Primers used for real-time qPCR experiments.

Name	Sequence (5'-->3')	TaqMan Probes
**Nono_Fw**	TCT TTT CTC GGG ACG GTG GAG	FAM-CGT GCA GCG TCG CCC ATA CTC CGA GC-BHQ1
**Nono_rev**	GTC TGC CTC GCA GTC CTC ACT	
**Sirt2_fw**	CAG GCC AGA CGG ACC CCT TC	FAM-TGA TGG GCC TGG GAG GTG GCA TGG A-BHQ1
**Sirt2_rev**	AGG CCA CGT CCC TGT AAG CC	
**Cdk5_fw**	GGT GAA CGT CGT GCC CAA GC	FAM-TGC CAC GGG GAG GGA CCT GTT GCA-BHQ1
**Cdk5_rev**	TGA GAT GCG CTG CAC AGG GT	
**Atp5h_fw**	TGC CCT GAA GAT TCC TGT GCC T	FAM-TCT CCT CCT GGT CCA CCA GGG CTG TGT-BHQ1
**Atp5h_rev**	ACT CAG CAC AGC TCT TCA CAT CCT	
**pBmal1_fw**	GAT CCG AGT GCG GGT GCG	FAM-CGG GCG CTC GCA GCG AGC CA-BHQ1
**pBmal1_rev**	CGC AGC CAT GCC GAC ACT CA	
**Bmal1_fw**	GCA ATG CAA TGT CCA GGA AG	FAM-ACC GTG CTA AGG ATG GCT GTT CAG CA-BHQ1
**Bmal1_rev**	GCT TCT GTG TAT GGG TTG GT	
**pPer2_fw**	CAC CCA CCC ACC CAC GTG AT	FAM-CCC TCG TGC AGG TAC CTG GAG AGC-BHQ1
**pPer2_rev**	GGC TGG GAA CTC GCA CTT CCT T	
**Per2_fw**	TCC ACA GCT ACA CCA CCC CTT A	FAM-CCG CTG CAC ACA CTC CAG GGC G-BHQ1
**Per2_rev**	TTT CTC CTC CAT GCA CTC CTG A	
**pPer1_fw**	CCC TGG CTC CTC CAG TGA T	FAM-TGG AGC AGG AGG CCC AGG CCC-BHQ1
**pPer1_rev**	GCC GTG GAA GCC GTT ACT CA	
**Per1_fw**	ACC AGC TGG GCC GGT TTT GTG	FAM-CCC CTG GAG CCG CAA GGT GGC-BHQ1
**Per1_rev**	CTC ATT CAG GGG TGC CGT GC	
**Wdr5_fw**	CCA CAC AGA GCA AGC CCA C	FAM-GCC CTG AAG TTC ACC CTG GCT GGC C-BHQ1
**Wdr5_rev**	CAC AGA GGA CAC AGC TTT GGT GT	

Supplementary Table 5: Primers for cloning and mutagenesis for MDF cell experiments.

Name	Sequence (5'-->3')
**PER1 R1111A R1113A (*NotI*) fw**	**ccgc**GACTGAGCCTGGGGACCAG
**PER1 R1111A R1113A (*NotI*) rv**	**ccgc**AGCAGCCCCAGCTTCAGC
**PER1 full-length fw (*EcoRI*)**	CG**GAATTC**ATGAGTGGTCCCCTAGAAGGGGCCGATG
**PER1 full-length rv (*SalI*)**	GA**GTCGAC**TCATGTGCTGTCCAGGCCAAGCAGAGG G
**gPER1 promoter fragment (*EcoRI*) fw**	GA**GAATTC**TTTTAGGGCAGGGCTGGCATTTGCGTC
**gPER1 promoter fragment (*EcoRI*) rv**	GA**GAATTC**CATCGGCCCCTTCTAGGGGACCACTCA
**Linker (*EcoRI*/*SalI*) fw**	C**GAATTC**CATCAGAAGGCTGTAGACAA**GTCGAC**G
**Linker (*EcoRI*/*SalI*) rv**	CCGGC**GTCGAC**TTGTCTACAGCCTTCTGATG**GAATT**
**WDR5 full-length (*NheI*) fw**	GG**GCTAGC**GAGCCATGGCCAC
**WDR5 full-length (*SalI*) rv**	GC**GTCGAC**TTAGCAGTCACTC
**Wdr5 reverse transcription primer**	CAATCCGGGTGGCTTCCTTAA

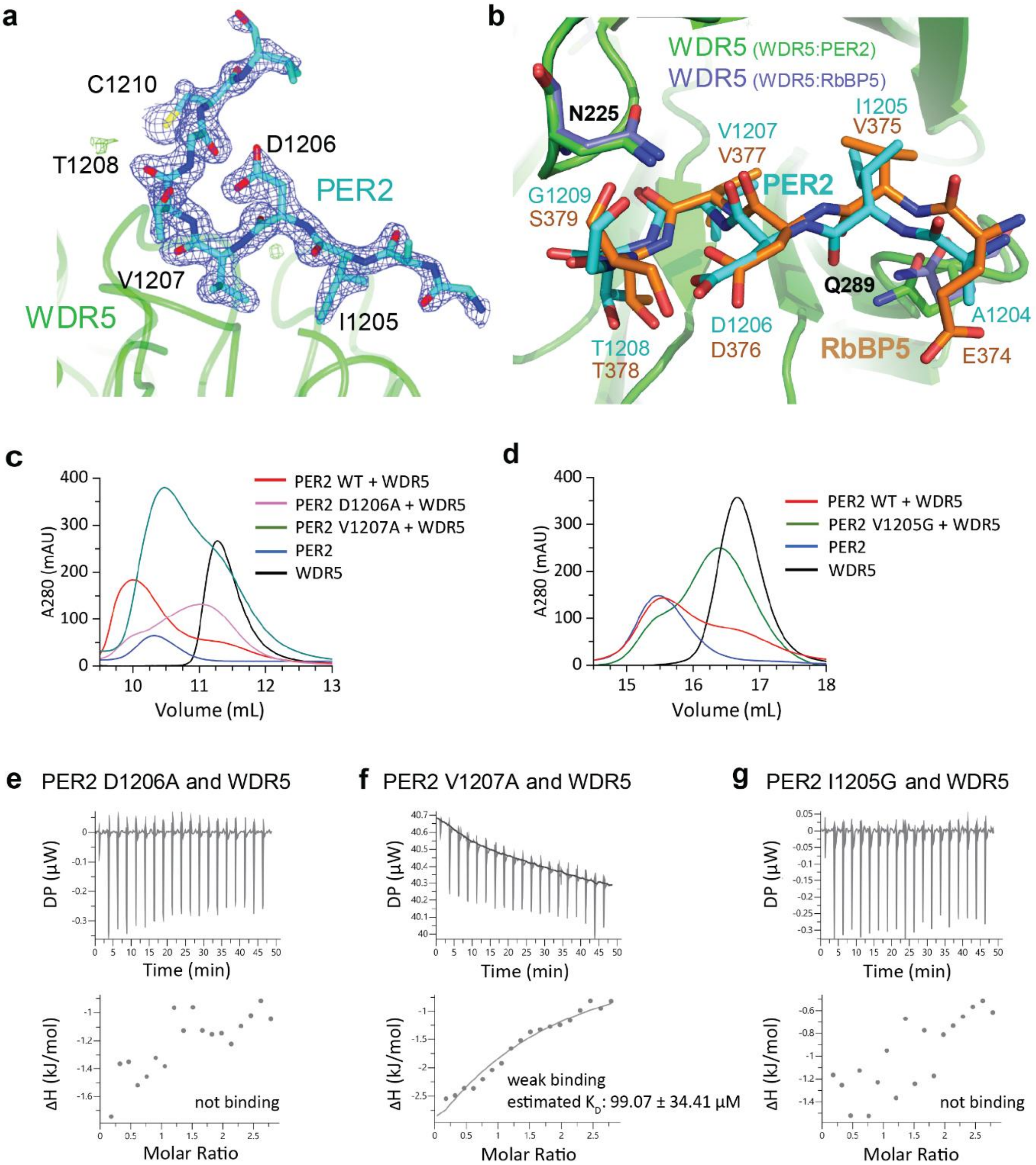

Supplementary Figure 1: Interaction of PER2 with WDR5.

a 2Fo-Fc electron density map of the WDR5-bound PER2 WBM motif peptide (Y^{1198}TGGLPTAIDVTGCV^{1211}) at 1.35σ (blue). Residues T1203 to V1211 of the PER2 peptide (cyan) are well resolved in the electron density. T1203 is modelled as alanine. WDR5 is shown in green.

b Close-up view of the WBM motif of RbBP5 (orange, PDB 2XL3) [1] and PER2 (cyan) binding to WDR5. WDR5 of the WDR5/PER2 complex is shown in green. N225 and Q289 of the WDR5/RbBP5 complex are shown in purple. The structures were superimposed on WDR5 with an RMSD of 0.271 Å.

c,d PER2 WBM motif mutations (I1205G, D1206A, V1207A) disrupt WDR5 interactions with the PER2 CRY binding domain (CBD, 1132-1252) in SEC. **c** SEC chromatograms of excess WDR5 22-334 mixed 3:1 with PER2 1132-1252 WT (red), PER2 D1206A (magenta)

or PER2 V1207A (green). WDR5 22-334 alone (black) and PER2 1132-1252 WT alone (blue) are shown for comparison. An S75 10/300 column was used. **d** SEC chromatograms of excess WDR5 mixed 3:1 with PER2 WT (red) or PER2 I1205G (green). WDR5 22-334 alone (black) and PER2 1132-1252 WT alone (blue) are shown for comparison. An S200 10/300 column was used. SEC results are representative of n=2 independent experiments.

e,f,g PER2 WBM motif mutations disrupt WDR5 interactions with the PER2 CBD in ITC. Representative ITC experiments (one of three independent replicates, n=3) for **e** PER2 1132-1252 D1206A, **f** V1207A and **g** I1205G with WDR5 22-334 as titrant are shown. The K_D value for PER2 1132-1252 V1207A **(f)** is the means of the triplicates +/- SD, also shown in Supplementary Table 2 and Fig. 2e. Upper panel: raw data, lower panel: integrated heat (points) with fit for a one-site binding model (line). For PER2 1132-1252 D1206A **(e)** and I1205G **(g)** no binding was observed.

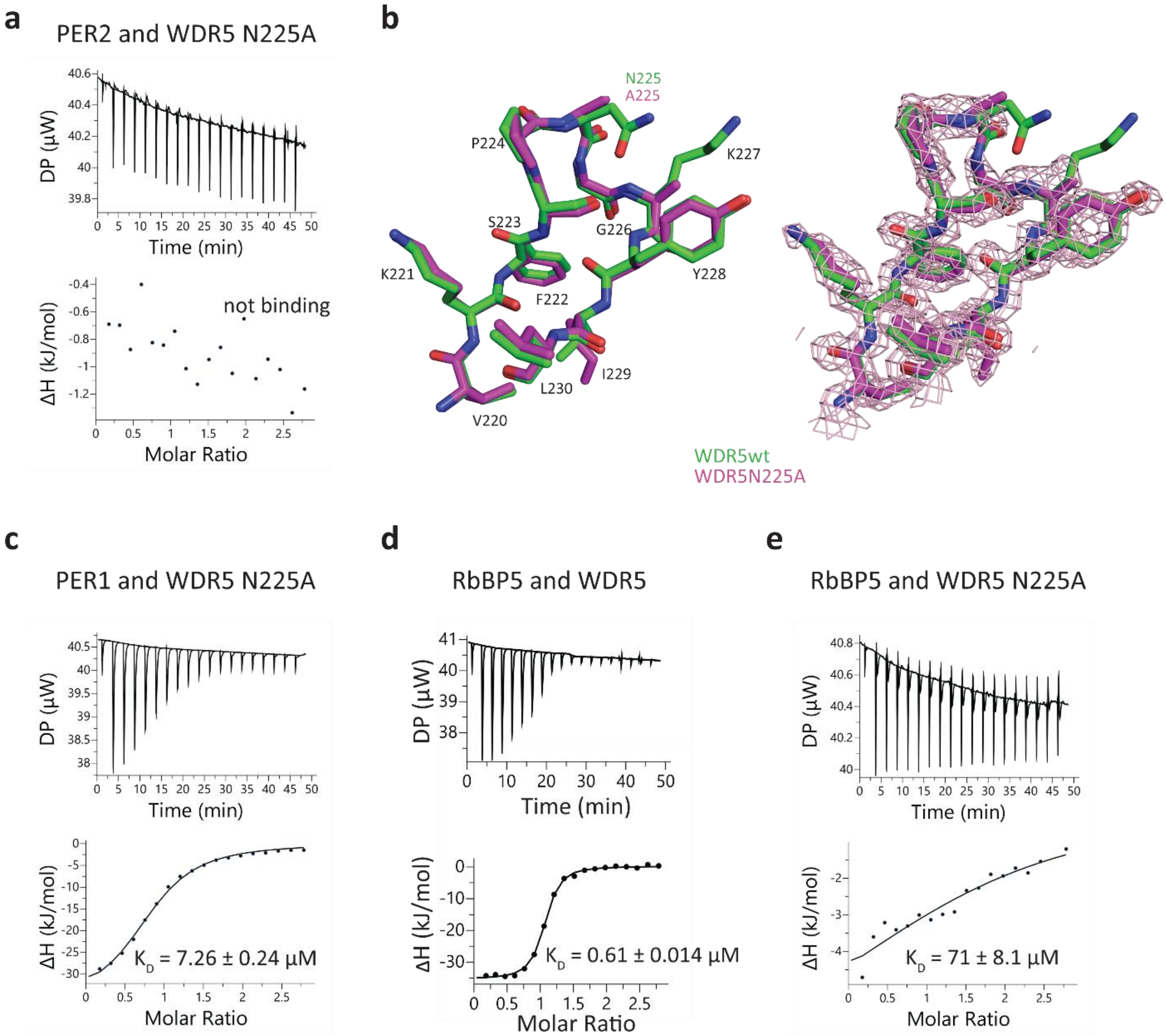

Supplementary Figure 2: Impact of WDR5 N225A mutation on PER2-WDR5- , PER1-WDR5- and RbBP5-WDR5 interaction.

a,c,d,e A representative ITC experiment for **a** PER2 1132-1252 with WDR5 22-334 N225A, **c** PER1 1013-1291 with WDR5 22-334 N225A, **d** RbBP5 with WDR5 22-334 WT, and **e** RbBP5 with WDR5 22-334 N225A is shown. The K_D values are the means of the triplicates with standard deviations (+/- SD), also shown in Supplementary Table 2. Upper panel: raw data, lower panel: integrated heat (points) with fit for a one-site binding model (line). For PER2 1132-1252 with WDR5 N225A (a) no binding was observed (Supplementary Table 2, Fig. 2e). ITC results are representative of n=3 independent experiments.

b Close-up view of the WDR5 N225A mutant crystal structure (magenta) superimposed with the WDR5 WT structure (green, PDB 2H9L) [2] around amino acid N225 (RMSD=0.343 Å). K227 is modeled as alanine in the WDR5 N225A structure due to conformational disorder. Right: 2Fo-Fc electron density map at 1.35σ (magenta) mapped to the WDR5 N225A structure (magenta) and overlayed with WDR5 WT structure (green).

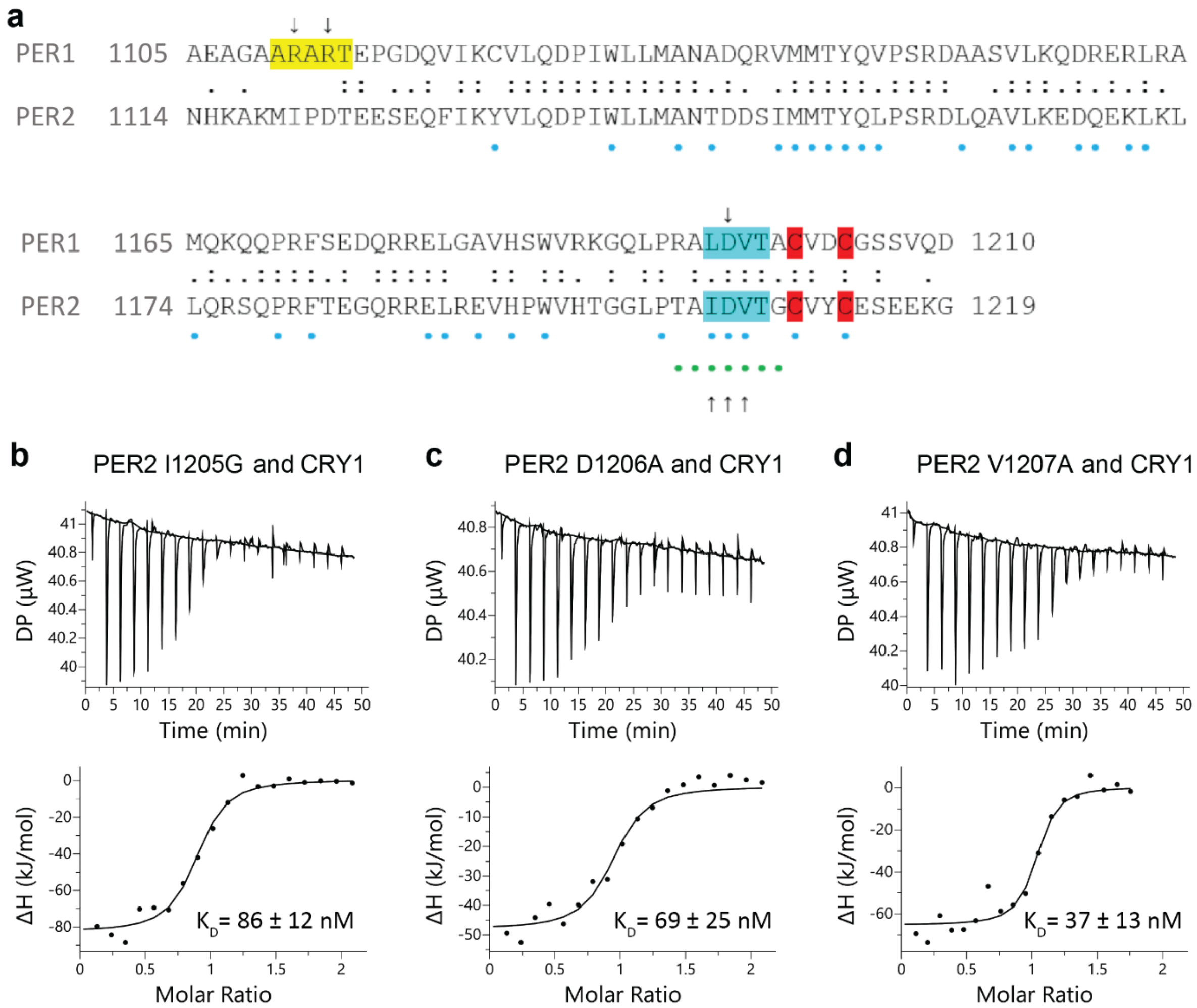

Supplementary Figure 3: Sequence alignment of PER1 and PER2 and interaction of PER2 1132-1252 with CRY1.

a Alignment of PER1 1105-1210 and PER2 1114-1219. Identical amino acids are shown by ":" and similar amino acids by ".". The WDR5 binding motifs are shown in cyan (WBM) and yellow (WIN). Important amino acids of PER2 for interaction with CRY1 (blue) (Schmalen *et al,* 2014) or WDR5 (green) (Fig. 2) are highlighted as dots. Amino acids mutated within this study are indicated by arrows under (PER2) or above (PER1) the alignment. Amino acids that interact with CRY1 or with the WDR5 WBM site are mostly conserved between PER1 and PER2. The WIN motif of PER1 is not conserved in PER2.

b-d A representative ITC experiment for **b** PER2 1132-1252 I1205G, **c** PER2 D1206A and **d** PER2 V1207A (titrant) with CRY1 1-496 (PHR) is shown. The K_D values are the means of the triplicates +/- SD, also shown in Supplementary Table 2 and Fig. 2h. Upper panel: raw data, lower panel: integrated heat (points) with fit for a one-site binding model (line). ITC results are representative of n=3 independent experiments.

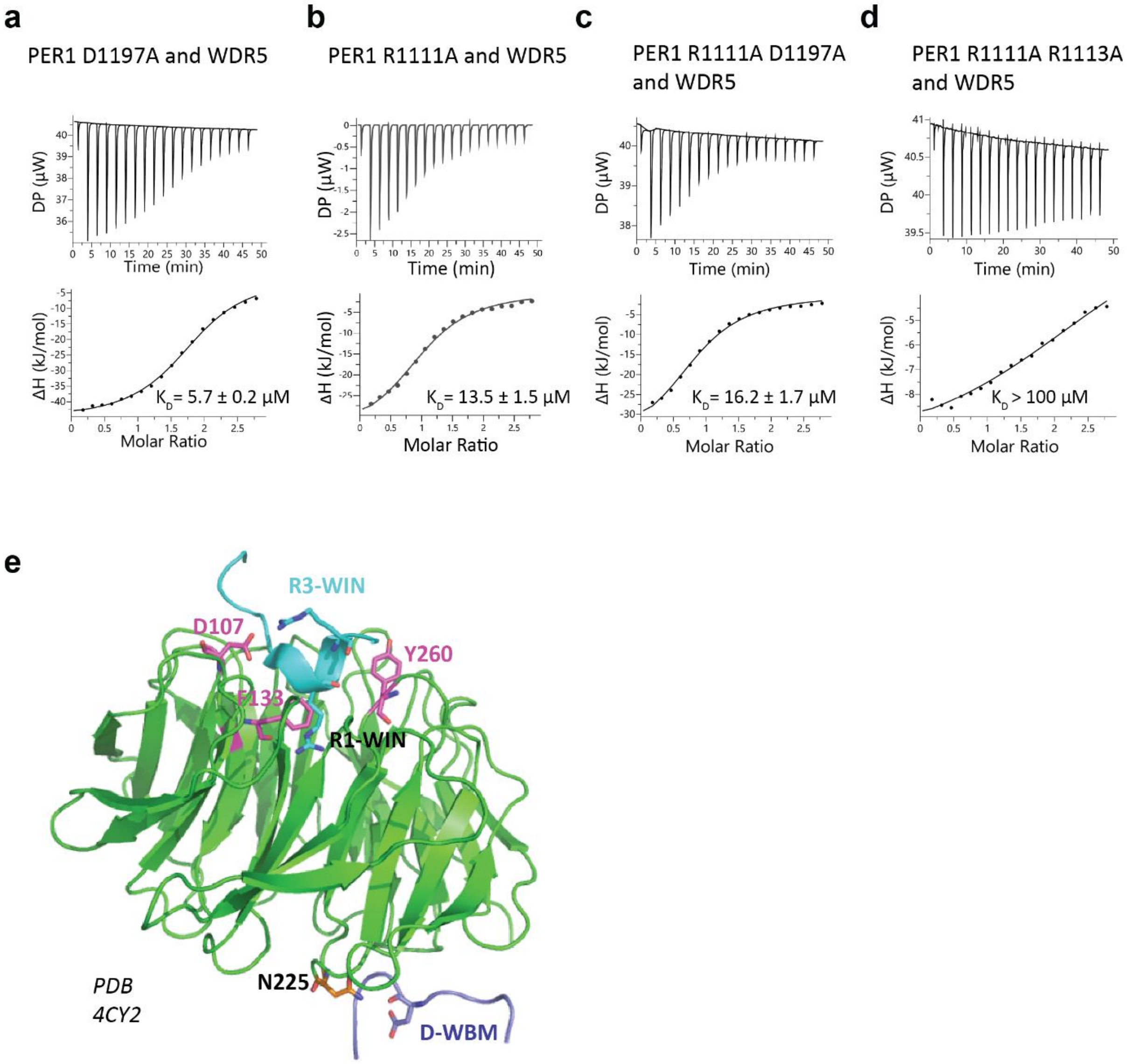

Supplementary Figure 4: Interaction of PER1 1013-1291 with WDR5.

a-d A representative ITC experiment for **a** PER1 1013-1291 D1197A, **b** PER1 R1111A, **c** PER1 R1111A D1197A and **d** PER1 R1111A R1113A with WDR5 22-334 (titrant) is shown. The K_D values are the means of the triplicates +/- SD, also shown in Supplementary Table 2 and Fig. 3c. Upper panel: raw data, lower panel: integrated heat (points) with fit for a one-site binding model (line). For PER1 R1111A R1113A and WDR5 (d) only very weak binding was observed, with an estimated K_D > 100 μM. ITC results are representative of n=3 independent experiments.

e Overview of the structure of WDR5 (green) bound to a KANSL1 WIN motif peptide (cyan) in the WIN motif binding pocket and to a KANSL2 WBM motif peptide (purple) in the WBM motif binding pocket of WDR5 (PDB 4CY2) [3]. Important amino acids in the WIN motif binding pocket of WDR5 (D107, F133, Y260) are shown in pink. The two WIN motif arginine residues of KANSL1 (denoted R1-WIN, R3-WIN) correspond to R1111 (R1) and R1113 (R3) of PER1. N225 in the WBM motif binding pocket of WDR5 is highlighted in orange. The conserved aspartate (denoted D-WBM) in the KANSL2 WBM motif peptide corresponds to D1197 in PER1 and D1206 in PER2.

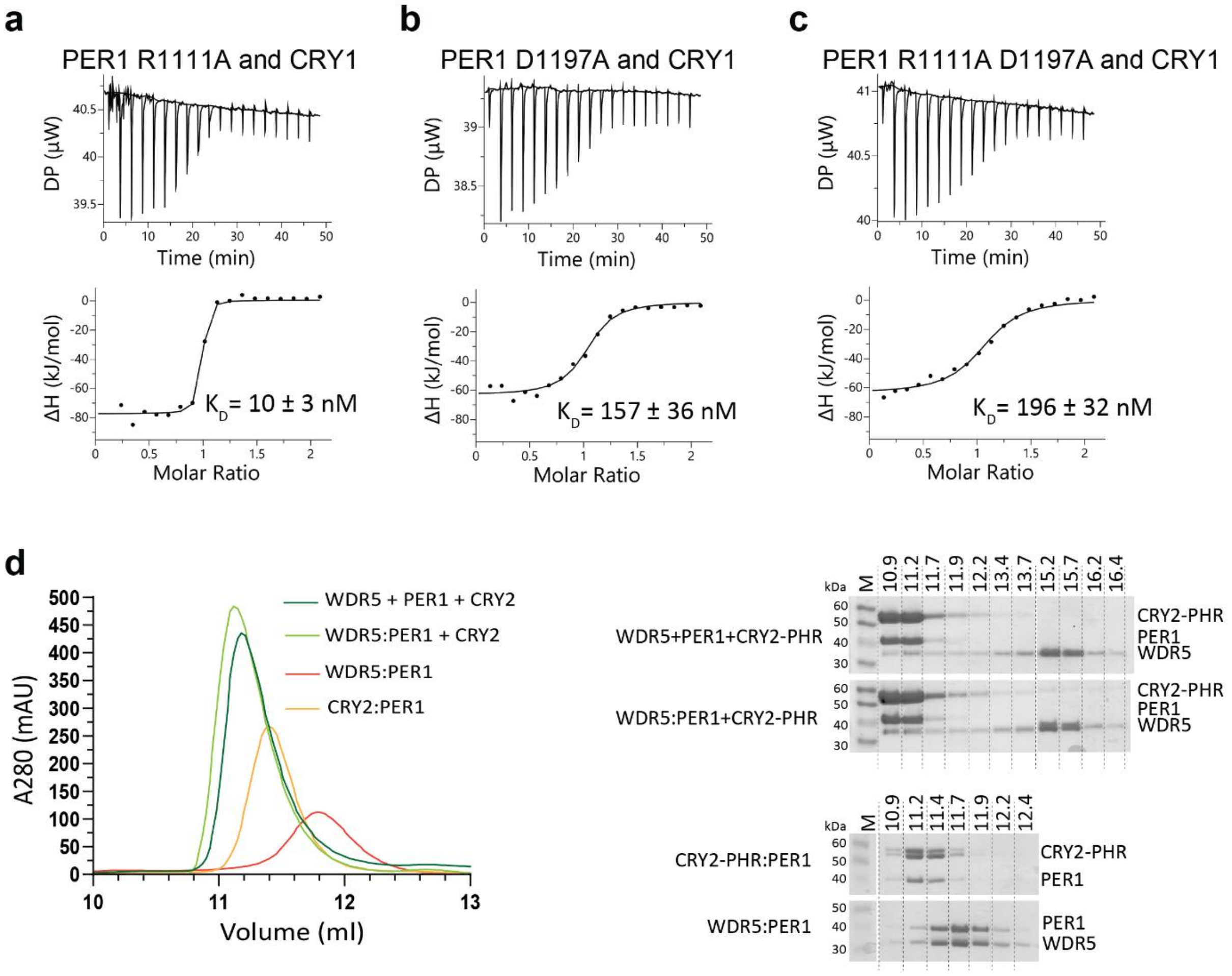

Supplementary Figure 5: **Interaction of PER1 1013-1291 with CRY1 and CRY2.**

a-c A representative ITC experiment for **a** PER1 1013-1291 R1111A, **b** PER1 D1197A and **c** PER1 R1111A D1197A (titrant) with CRY1 1-496 (PHR) is shown. The K_D values are the means of the triplicates +/- SD, also shown in Supplementary Table 2 and Fig. 3c. Upper panel: raw data, lower panel: integrated heat (points) with fit for a one-site binding model (line). ITC results are representative of n=3 independent experiments.

d Chromatograms (left) and SDS-PAGE analyses (right) of SEC (S200 10/300) of the PER1 1013-1291 interaction with CRY2 1-512 (PHR) and WDR5 22-334. SDS-PAGE top: equimolar mixture of PER1, CRY2 and WDR5, 1 h incubation (dark green chromatogram); second: preformed PER1/WDR5 complex (ON), subsequently incubated with equimolar CRY2 for 1h (light green chromatogram). bottom: dimeric PER1/CRY2 and PER1/WDR5 complexes (orange and red chromatograms) start to elute at 11.2 and 11.4 mL. Some trimeric PER1/WDR5/CRY2 complex is formed, that starts to elute before 11.2 mL. Non-incorporated WDR5 elutes after 15 ml. SEC results are representative of n=3 independent experiments.

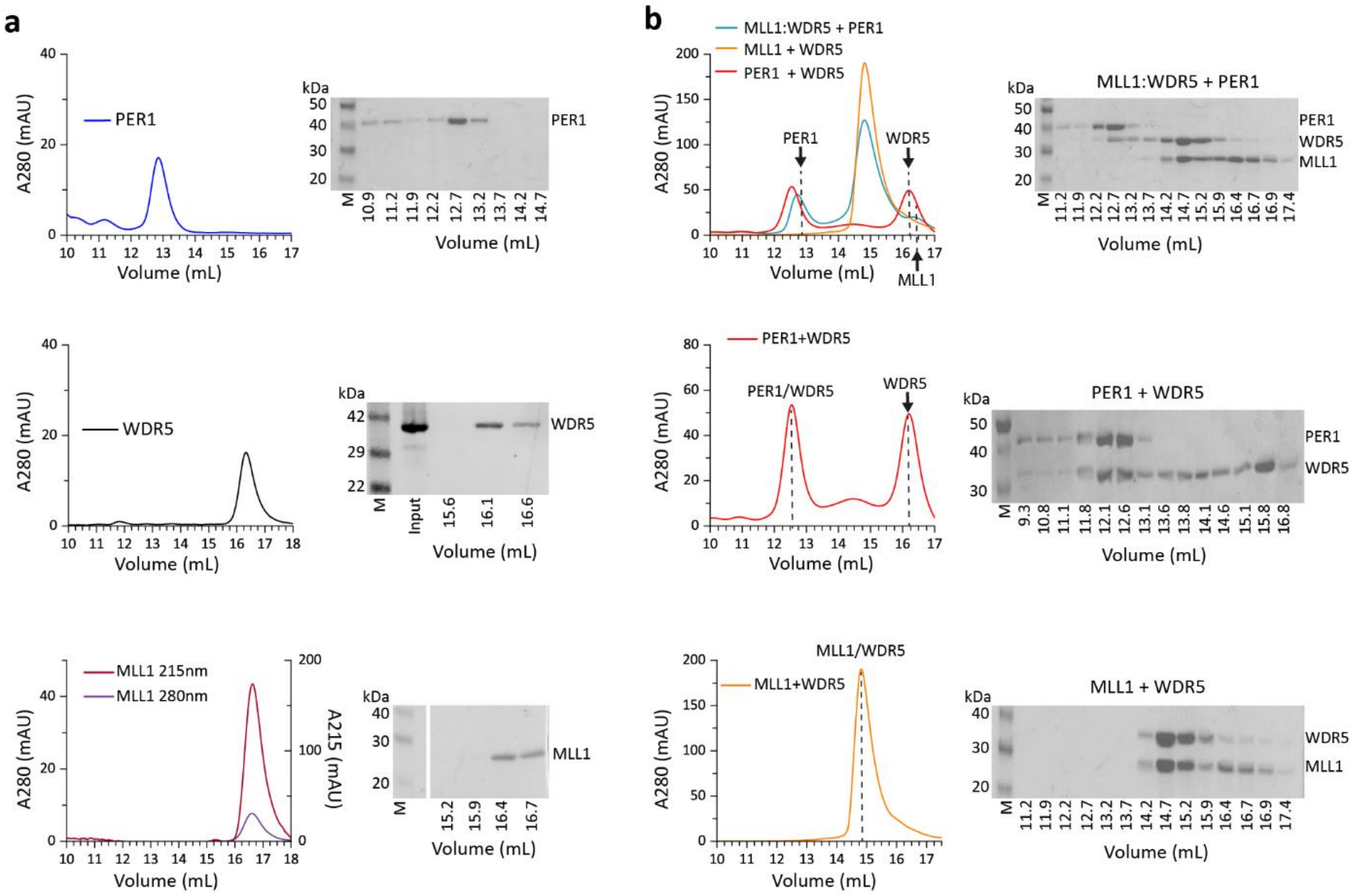

Supplementary Fig. 6: Interaction interplay of PER1 1013-1291 and MLL1 3748-3969 on WDR5.

a Chromatograms (left) and SDS-PAGE analyses (right) of the SEC (S200 10/300) runs with the individual proteins PER1 1013-1291 (top), WDR5 22-334 (middle) and hMLL1 3748-3969 (WIN motif + SET domain) (bottom). As MLL1 has a very low absorbance at 280 nm, we also show the chromatogram for absorbance at 215 nm.

b Chromatograms (left) and SDS-PAGE analyses (right) of SEC (S200 10/300) runs with dimeric PER1/WDR5 (middle) and MLL1/WDR5 (bottom) complexes. Top: preformed MLL1/WDR5 complex incubated with equimolar PER1 (light blue chromatogram). PER1 partially displaces MLL1 from the preformed MLL1/WDR5 complex (identical to Fig. 4b, added for direct comparison with SEC runs of dimeric PER1/WDR5- and MLL1/WDR5 complexes and individual PER1, MLL1 and WDR5 proteins). SEC results are representative of n=3 independent experiments.

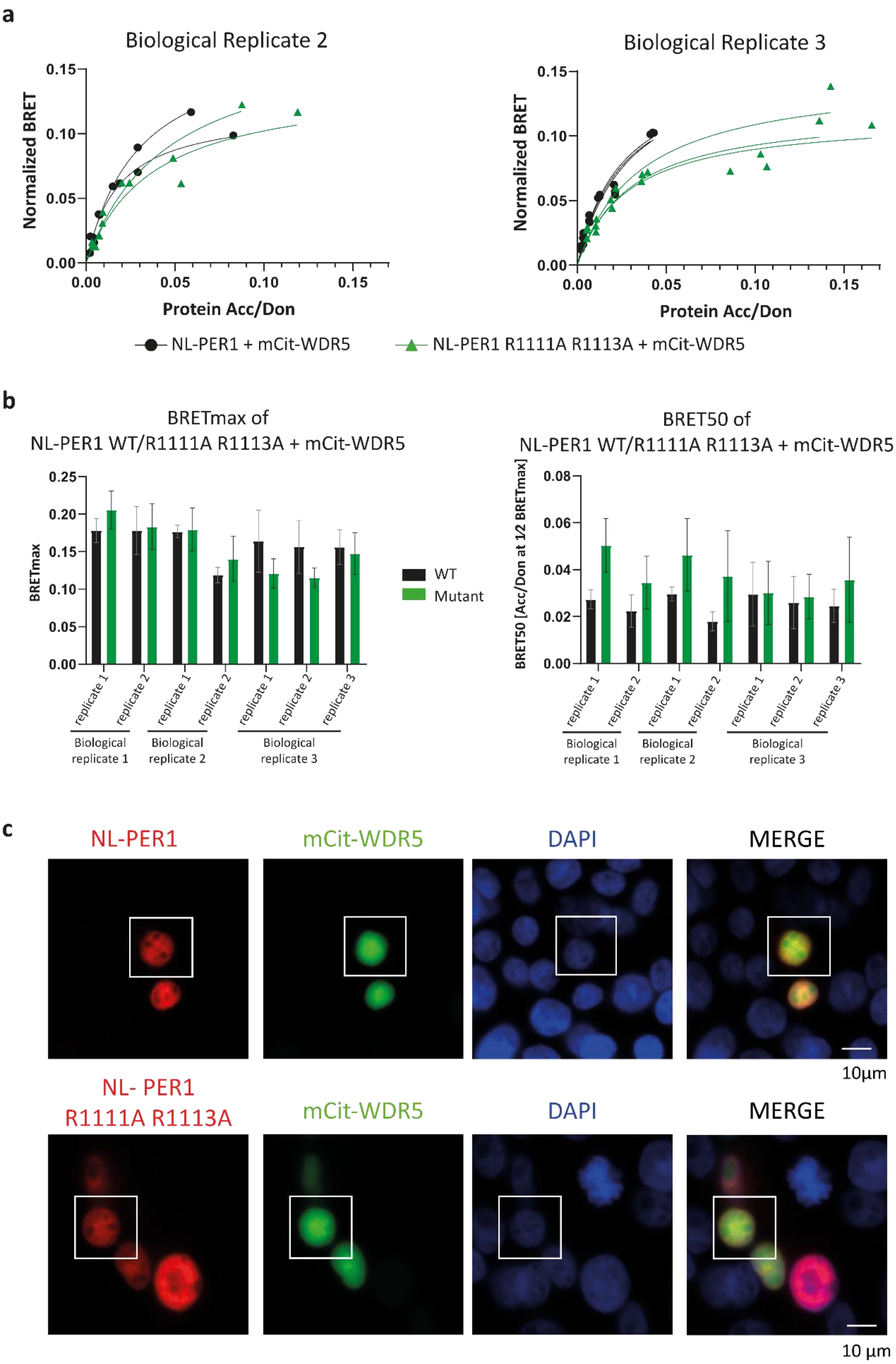

Supplementary Figure 7: The PER1 WIN motif mediates WDR5 interactions of full-length PER1 in HEK293 cells

a Normalized BRET signal (remaining 2 of 3 biological replicates) of full-length NL-myc-PER1+mCit-WDR5 (black) or NL-myc-PER1 R1111A R1113A+mCit-WDR5 (green) plotted

against the measured ratio of acceptor (mCitrine) over donor (NL, Nano-Luc) proteins (Acc/Don). Donor:acceptor plasmid ratios were 2ng:12.5ng, 2ng:25ng, 2ng:50ng, 2ng:100ng, 1ng:100ng, 1ng:200ng. Curves were fit with a one-site specific binding model (GraphPad Prism v9.5.1).

b $BRET_{max}$ (left) and $BRET_{50}$ (Acc/Don ratio at half of $BRET_{max}$, right) values of individual biological and technical replicates as estimated from fits in **a** and Fig. 5a. Error bars show +/- SE of curve fit to data points.

c Colocalization of WDR5 with wildtype PER1 and PER1 R1111A R1113A WIN site mutant. Immunohistochemistry staining of HEK293T cells co-transfected with mCit-WDR5 and wildtype (top) or mutant (bottom) NL-cmyc-PER1 (full-length). Data are representative of two independent (n=2) transfections. Similar colocalization patterns were observed across multiple fields of view in each experiment. Red channel: cmyc-antibody, green channel: mCitrine, blue channel= DAPI staining. White box: cropped region with one cell as depicted in Fig. 5c.

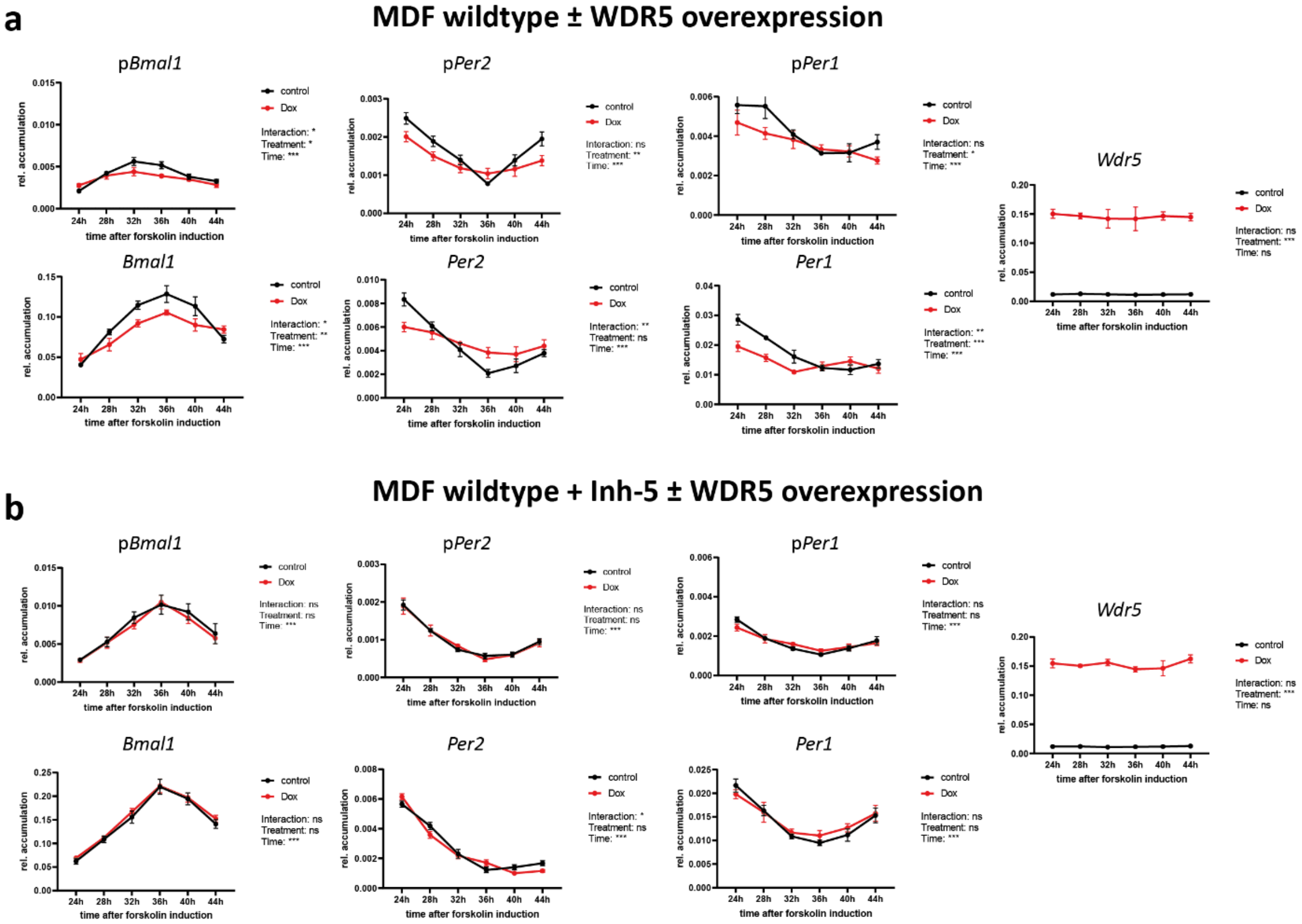

Supplementary Figure 8: WDR5 overexpression impacts *Bmal1, Per2* and *Per1* expression in absence of Inh-5.

a qPCR data of MDF wildtype cells without doxycycline treatment (control, black) and treated with doxycycline (Dox, red), resulting in Dox-induced overexpression of WDR5. The relative accumulation of unspliced precursor pre-mRNA (*pBmal1, pPer2, pPer1*) and mRNA (*Bmal1, Per2, Per1, Wdr5)* is plotted against the time after forskolin synchronization. Data are presented as mean +/- SEM of n=4 independent biological replicates. Two-way-ANOVA was employed to test for treatment, interaction and time P-values. Exact p-values are shared within the source data file for the respective figure. ns= $P \geq 0.05$, *=P≤0.05, **=P≤0.01, ***=P≤0.001.

b as (**a**), but MDF cells were constantly treated with 10 µM Inh-5. Exact p-values are shared within the source data file for the respective figure.

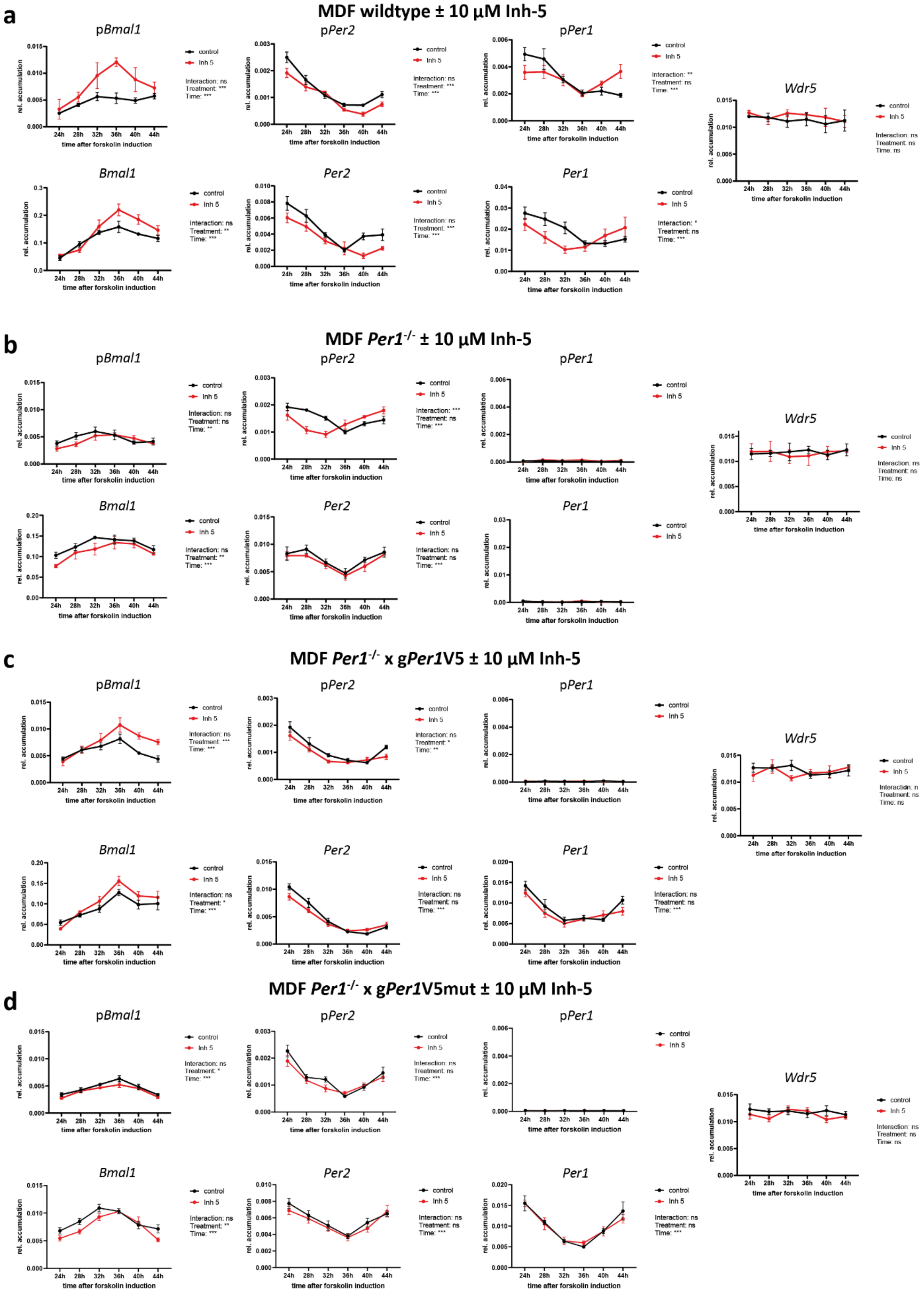

Supplementary Figure 9: Inh-5 enhances *Bmal1* transcription and reduces *Per2* transcription in presence of PER1 with an intact WIN motif. qPCR data of wildtype MDF cells **(a)**, MDF *Per1*$^{-/-}$ cells **(b)** and MDF *Per1*$^{-/-}$ cells expressing V5-tagged PER1 **(c)** or the V5-tagged PER1 R1111A R1113A mutant **(d)** from a *Per1* promoter fragment. The relative

accumulation of pre-mRNA (*pBmal1, pPer2, pPer1*) and mRNA (*Bmal1, Per2, Per1, Wdr5*) without Inh-5 treatment (control, black) and treated with 10 μM Inh-5 (red) is plotted against the time after forskolin synchronization. Data are presented as mean +/- SEM of n=4 independent biological replicates. Two-way-ANOVA was employed to test for treatment, interaction and time P-values. Exact p-values are shared within the source data file for the respective figure. ns=P≥0.05, *=P≤0.05, **=P≤0.01, ***=P≤0.001.

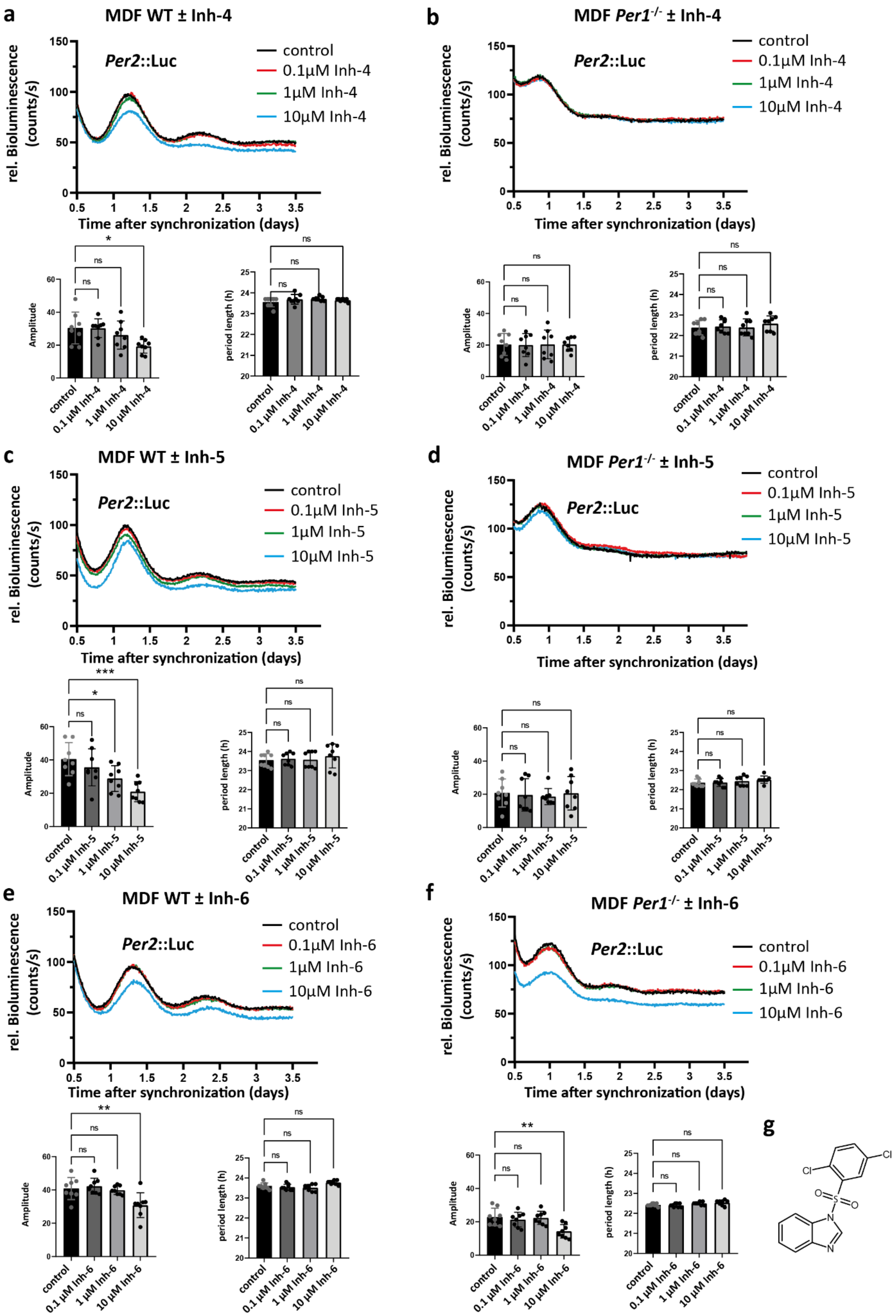

a
MDF WT ± Inh-4
Per2::Luc
control
0.1µM Inh-4
1µM Inh-4
10µM Inh-4
rel. Bioluminescence (counts/s)
Time after synchronization (days)
Amplitude
period length (h)
control
0.1 µM Inh-4
1 µM Inh-4
10 µM Inh-4
b
MDF Per1-/- ± Inh-4
c
MDF WT ± Inh-5
0.1µM Inh-5
1µM Inh-5
10µM Inh-5
d
MDF Per1-/- ± Inh-5
e
MDF WT ± Inh-6
0.1µM Inh-6
1µM Inh-6
10µM Inh-6
f
MDF Per1-/- ± Inh-6
g
Cl
O=S=O
N

Supplementary Figure 10: Impact of WDR5 WIN site- and WBM site inhibitors on PER2::Luc accumulation.

a,b Normalized bioluminescence raw counts of late passage (P > 20) MDF wildtype or MDF *Per1*$^{-/-}$ *Per2*::Luc cells with and without the WDR5 WIN site inhibitor Inh-4 (n = 8 independent treatments). Ordinary One-way ANOVA alongside Dunnett's multiple comparison test relative to control was performed for statistical amplitude and period length analysis shown below each graph. Amplitude of wildtype cells: p-values for 0.1µM, 1µM and 10µM Inh-4 relative to control were p= >0.999 (ns), p=0.519 (ns) and p=0.014 (*), respectively. Period length of wildtype cells: p-values for 0.1µM, 1µM and 10µM Inh-4 relative to control were p=0.289 (ns), p=0.177 (ns) and p=0.534 (ns), respectively. Amplitude of *Per1*$^{-/-}$ cells: p-values for 0.1µM, 1µM and 10µM Inh-4 relative to control were p=0.999 (ns), p= >0.999 (ns) and p= >0.999 (ns), respectively. Period length of *Per1*$^{-/-}$ cells: p-values for 0.1µM, 1µM and 10µM Inh-4 relative to control were p=0.973 (ns), p=0.999 (ns) and p=0.568 (ns), respectively. Data are presented as mean values +/- SEM. Individual amplitude/period values for 8 independent treatments (n=8) are shown as dots. **c,d** Normalized bioluminescence raw counts of late passage (P > 20) MDF wildtype or MDF *Per1*$^{-/-}$ *Per2*::Luc cells with and without the WDR5 WIN site inhibitor Inh-5 (n = 8 independent treatments). Ordinary One-way ANOVA alongside Dunnett's multiple comparison test relative to control was performed for statistical amplitude and period analysis shown below each graph. Amplitude of wildtype cells: p-values for 0.1µM, 1µM and 10µM Inh-5 relative to control were p= 0.566 (ns), p=0.038 (*) and p=0.0004 (***), respectively. Period length of wildtype cells: p-values for 0.1µM, 1µM and 10µM Inh-5 relative to control were p=0.975 (ns), p=0.997 (ns) and p=0.626 (ns), respectively. Amplitude *of Per1*$^{-/-}$ cells: p-values for 0.1µM, 1µM and 10µM Inh-5 relative to control were p=0.985 (ns), p=0.922 (ns) and p= >0.999 (ns), respectively. Period length of *Per1*$^{-/-}$ cells: p-values for 0.1µM, 1µM and 10µM Inh-5 relative to control were p=0.999 (ns), p=0.750 (ns) and p=0.310 (ns), respectively. Data are presented as mean values +/- SEM. Individual amplitude/period values for 8 independent treatments (n=8) are shown as dots. **e,f** Normalized bioluminescence raw counts of late passage (P > 20) MDF wildtype or MDF *Per1*$^{-/-}$ *Per2*::Luc cells with and without the WDR5 WBM site inhibitor Inh-6 (n = 8 independent treatments). Ordinary One-way ANOVA alongside Dunnett's multiple comparison test relative to control was performed for statistical amplitude and period analysis shown below each graph. Amplitude of wildtype cells: p-values for 0.1µM, 1µM and 10µM Inh-6 relative to control were p= 0.924 (ns), p=0.971 (ns) and p=0.004 (**), respectively. Period length of wildtype cells: p-values for 0.1µM, 1µM and 10µM Inh-6 relative to control were p=0.720 (ns), p=0.488 (ns) and p=0.057 (ns), respectively. Amplitude of *Per1*$^{-/-}$ cells: p-values for 0.1µM, 1µM and 10µM Inh-6 relative to control were p=0.806 (ns), p=0.988 (ns) and p=0.002 (**), respectively. Period length of *Per1*$^{-/-}$ cells: p-values for 0.1µM, 1µM and 10µM Inh-6 relative to control were p=0.914 (ns), p=0.287 (ns) and p=0.065 (ns), respectively. Data are presented as mean values +/- SEM. Individual amplitude/period values for 8 independent treatments (n=8) are shown as dots. **a-f** The MDF wildtype or MDF *Per1*$^{-/-}$ *Per2*::Luc cells were synchronized with forskolin, treated with the indicated concentrations of inhibitor, and the PER2::Luc bioluminescence was recorded for 3.5 days. **g** Chemical structure of Inh-6. ns=P≥0.05, *=P≤0.05, **=P≤0.01, ***=P≤0.001.

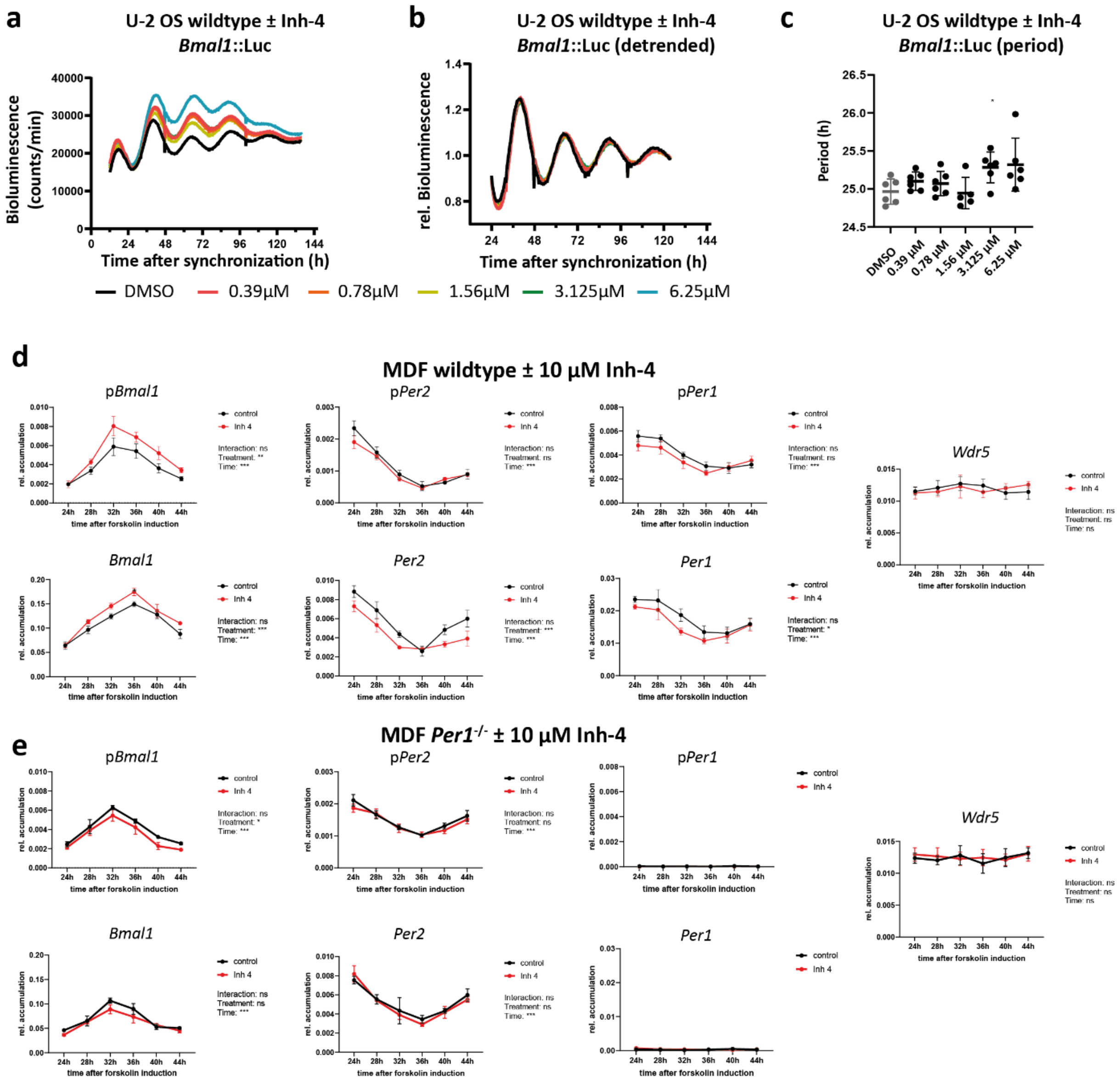

Supplementary Figure 11: Inh-4 impacts *Bmal1*::Luc circadian oscillations and affects *Bmal1*- and *Per2* transcription in a PER1 dependent manner.

a Bioluminescence raw counts of U-2 OS wildtype cells (n =5-6 independent treatments). The U-2 OS *Bmal1*::Luc cells were synchronized with 1 µM dexamethasone, washed and treated with different Inh-4 concentrations and the bioluminescence was recorded for 5 days.

b Detrended data from (**a**).

c Period length analysis of (**b**) was performed using the Chronostar software [4]. Subsequently, the period was plotted against increasing concentrations of Inh-4. Data are presented as mean +/- SD.

d,e qPCR data of MDF wildtype cells **(d)** and MDF *Per1*$^{-/-}$ cells **(e).** The relative accumulation of pre-mRNA (*pBmal1*, *pPer2*, *pPer1*) and mRNA (*Bmal1*, *Per2*, *Per1*, *Wdr5*) without Inh-4 treatment (control, black) and treated with 10 µM Inh-4 (red) is plotted against the time after forskolin synchronization. Data are presented as mean +/- SEM of

n=4 independent biological replicates. Two-way ANOVA was employed to test for treatment, interaction and time P values. Exact p-values are shared within the source data file for the respective figure. ns=P≥0.05, *=P≤0.05, **=P≤0.01, ***=P≤0.001.

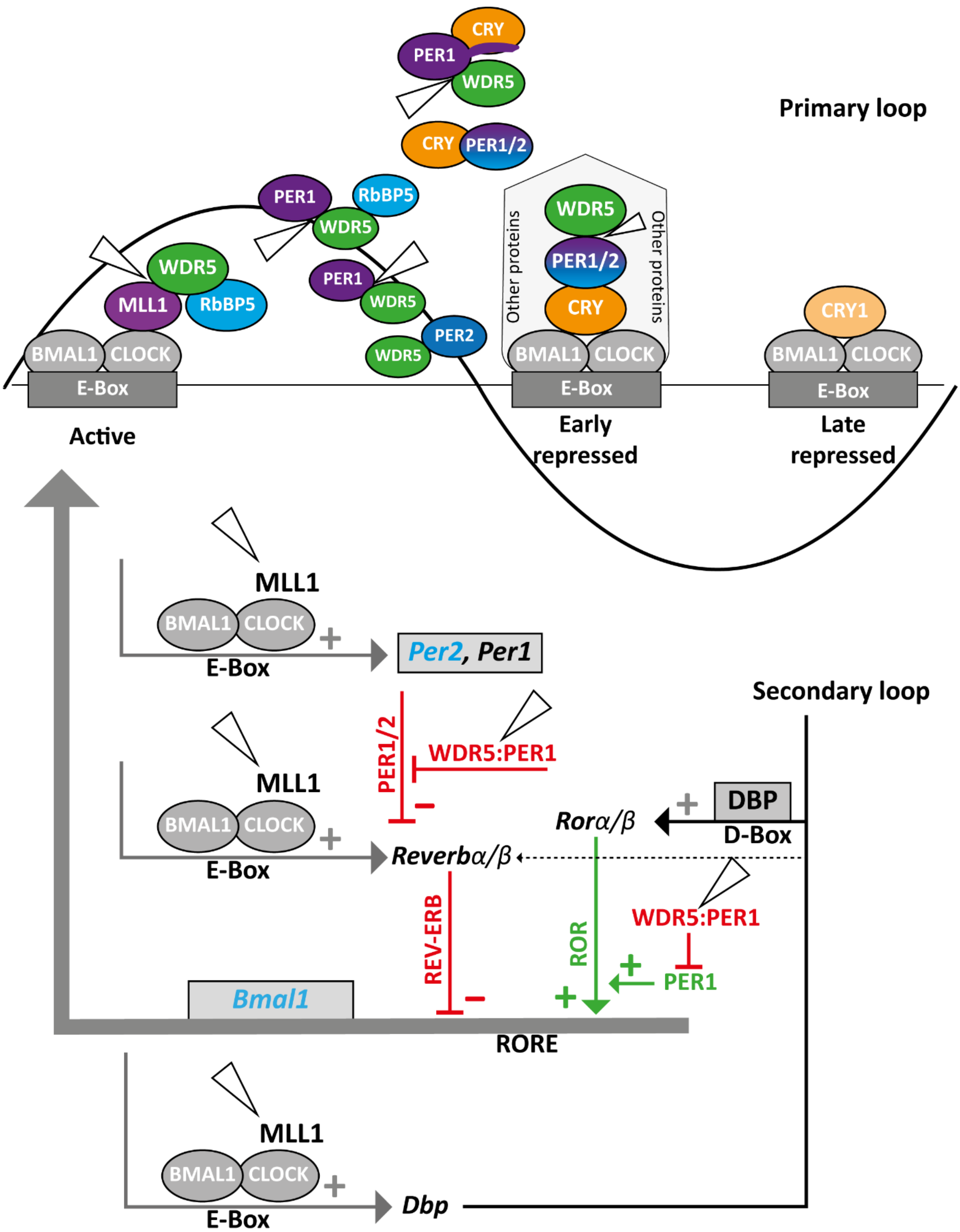

Supplementary Figure 12: Suggested roles of the PER1/WDR5 complex in the primary CLOCK/BMAL1/PER/CRY- and secondary BMAL1/REVERB/ROR/DBP feedback loop of the mammalian circadian oscillator.

Primary loop (top): WDR5 (green) is an MLL1 complex component associated with active CLOCK/BMAL1 and was found in a repressive PER/CRY containing complex. PER1/WDR5-, PER2/WDR5-, PER1/CRY-, PER2/CRY-, PER1/WDR5/RbBP5- and PER1/WDR5/CRY-complexes are depicted at the transition from the active state to the early repressed state

of CLOCK/BMAL1. These complexes may or may not coexist and could re-equilibrate dynamically over time, depending on the local relative concentration of the binding partners. Inh-5 could reduce *Per2* expression in rhythmic MDF cells by disrupting the activating MLL1/WDR5 complex or by disrupting the PER1/WDR5 complex and thereby enhancing CLOCK/BMAL1 repression. As Inh-5 effects on *Per2* RNA levels are not observed in *Per1*$^{-/-}$ MDF cells, we propose that PER1/WDR5 complexes negatively affect CLOCK/BMAL1 repression and thereby enhance *Per2* expression, and Inh-5 negates this effect (see discussion in main text).

Secondary loop (bottom): *Bmal1* expression is activated by ROR and repressed by REV-ERB. *Reverb* expression is activated by CLOCK/BMAL1 and repressed by CRY and PER1/2. *Ror* expression is activated by DBP, which itself is regulated by BMAL1/CLOCK. PER1/WDR5 complexes may negatively regulate PER1-dependant *Reverb* repression and/or PER1 coactivation of ROR, which would reduce *Bmal1* expression. Inh-5 could enhance *Bmal1* expression by disrupting the PER1/WDR5 complex. Additionally, Inh-5 could enhance *Bmal1* expression by disrupting the MLL1/WDR5 complex, which would reduce *Reverb* expression. In *Per1*$^{-/-}$ ^{MDF} MDF cells, Inh-5 may lead to reduced *Bmal1* expression by reducing DBP-dependent *Ror* activation (via MLL1 activation of DBP). Potential effects of Inh-5 on PER1/WDR5- or MLL1/WDR5 complexes are indicated by white triangles. See discussion in main text for further explanations.

References for Supplementary

1. Odho, Z., Southall, S. M. & Wilson, J. R. Characterization of a novel WDR5-binding site that recruits RbBP5 through a conserved motif to enhance methylation of histone H3 lysine 4 by mixed lineage leukemia protein-1. *The Journal of biological chemistry* **285,** 32967–32976; 10.1074/jbc.M110.159921 (2010).

2. Schuetz, A. *et al.* Structural basis for molecular recognition and presentation of histone H3 by WDR5. *The EMBO journal* **25,** 4245–4252; 10.1038/sj.emboj.7601316 (2006).

3. Dias, J. *et al.* Structural analysis of the KANSL1/WDR5/KANSL2 complex reveals that WDR5 is required for efficient assembly and chromatin targeting of the NSL complex. *Genes & development* **28,** 929–942; 10.1101/gad.240200.114 (2014).

4. Maier, B., Lorenzen, S., Finger, A.-M., Herzel, H. & Kramer, A. Searching Novel Clock Genes Using RNAi-Based Screening. *Methods in molecular biology (Clifton, N.J.)* **2130,** 103–114; 10.1007/978-1-0716-0381-9_8 (2021).
