# Supplementary material for: Role of PER-WDR5 interactions in the mammalian circadian clock
Source: Nat Commun. 2026 Sep 8;17:10627. doi: 10.1038/s41467-026-77583-0 (PMC13649712; doi:10.1038/s41467-026-77583-0)
Supplement: Supplementary file 3 [file 41467_2026_77583_MOESM3_ESM.pdf]

nature portfolio

Peer Review File

Role of PER-WDR5 interactions in the mammalian circadian clock

Corresponding Author: Professor Eva Wolf

This manuscript has been previously reviewed at another journal. This document only contains information relating to versions considered at Nature Communications.

This file contains all reviewer reports in order by version, followed by all author rebuttals in order by version.

Version 0:

Reviewer comments:

Reviewer #1

(Remarks to the Author)
The authors have done an excellent job revising the manuscript. The data are convincing, and all of my questions have been thoroughly and satisfactorily addressed.

Reviewer #2

(Remarks to the Author)
Overall, the manuscript has much improved. It makes a convincing argument that WDR5-PER1 interaction affect circadian clock regulation. I still have two concerns. First, the MDF data (Fig. 6) are not very convincing. To start with, compared to other fibroblast setups (see, e.g., the cited Brown paper – REF 14) the cells are not very rhythmic and dampen fast. This makes it difficult to assess circadian parameters. In line with this, the detected amplitude effect of Inh5 treatment cannot be seen in the traces (panel a). Moreover, Per1 deficient MDFs do not show stable rhythms so analysing amplitude (or even period) remains ambiguous (panel b). In addition, qPCR data rather point at amplitude enhancing effects of Inh5 treatment (panel c). Further, clear effects remain to be seen in Per1 deficient MDFs (panel d), albeit these are not easily explained.

Second, while the authors provide various reasons for doing so, I am somewhat concerned with the fact that so many – partially contradictory - data sets have been removed from the revised manuscript. While it is understandable that a consistent story is expected, they might provide important evidence for the interpretation of the results. The reader of the published paper will not have this information which, in the worst case, may misguide his/her understanding of the findings.

I understand the technical reasons for not directly testing for the temporal switch in WDR5 interactions. I recommend specifically mentioning this in the discussion and amending the interpretation of the findings accordingly.

Open Access This Peer Review File is licensed under a Creative Commons Attribution 4.0 International License, which permits use, sharing, adaptation, distribution and reproduction in any medium or format, as long as you give appropriate credit to the original author(s) and the source, provide a link to the Creative Commons license, and indicate if changes were made.
In cases where reviewers are anonymous, credit should be given to 'Anonymous Referee' and the source.
The images or other third party material in this Peer Review File are included in the article's Creative Commons license, unless indicated otherwise in a credit line to the material. If material is not included in the article's Creative Commons license and your intended use is not permitted by statutory regulation or exceeds the permitted use, you will need to obtain permission directly from the copyright holder.
To view a copy of this license, visit https://creativecommons.org/licenses/by/4.0/

Response to reviewer 2

Reviewer 2: Overall, the manuscript has much improved. It makes a convincing argument that WDR5-PER1 interaction affect circadian clock regulation. I still have two concerns. First, the MDF data (Fig. 6) are not very convincing. To start with, compared to other fibroblast setups (see, e.g., the cited Brown paper – REF 14) the cells are not very rhythmic and dampen fast. This makes it difficult to assess circadian parameters. In line with this, the detected amplitude effect of Inh5 treatment cannot be seen in the traces (panel a). Moreover, Per1 deficient MDFs do not show stable rhythms so analysing amplitude (or even period) remains ambiguous (panel b).

We thank the reviewer for the comments which helped us to further improve the manuscript.
The reduced rhythms of our MDF cells reflect the advanced passage numbers of the mouse dermal fibroblasts tested here. Advanced passages (>20) were necessary to obtain the rescue cell lines by lentiviral infection and selection. For consistency, we therefore measured all qPCR data (wildtype, *Per1*$^{-/-}$ and rescued *Per1*$^{-/-}$ cells) and our PER2::Luc oscillation data using MDF cells from similar (P>20) passage numbers.
Unfortunately, we were not able to retrieve a protocol, or extract from the paper REF-14 (Brown *et al, PLoS Biol* 2005, [1]) their exact method of infection and selection. The mouse strains used in the two studies (clearly mixed C57BL/6/SV129 background in REF-14 although stated isogenic, pure C57BL/6 in our study) essentially carry the same deletion of the *Per1* gene. We do not know the age of the animals used in REF-14, while we use as donors animals >3 month old. And they used tail fibroblasts, while we use ear fibroblasts. Steve Brown and colleagues did apparently not use primary cells after passage number 4 and their lentiviral infection did not necessitate a selection procedure afterwards because they observed a gain of reporter gene activity (personal communication Steve Brown with Jürgen Ripperger). To match closer to the results of REF-14, we therefore replaced Fig.6a,b with a PER2::Luc oscillation experiment using early passage (P=4) MDF cells, in which wildtype and *Per1*$^{-/-}$ MDF cells display more robust circadian rhythms. This allowed us to determine amplitude and period changes more confidently with less variation between replicates (smaller SEM, dots less spread out) and better statistics (new Fig. 6a,b) than with the data obtained from later passage MDF cells (Supplementary Fig. 10c,d). The original PER2::Luc oscillation data of later passage (P>20) Inh-5 treated MDF cells were placed for comparison in Supplementary Fig.10c,d together with the original PER2::Luc oscillation data with Inh-4 (Supplementary Fig.10a,b; former EDF 10d-g), and new data of later passage MDF cells treated with inhibitor 6 (Inh-6), which is a WDR5 WBM site inhibitor (Supplementary Fig.10e-g).
We can state the following: the effect of the WIN site inhibitor Inh-5 on early or late passage MDFs causes a reduced amplitude of PER2::Luc in wildtype MDF cells, but not in *Per1*$^{-/-}$ MDF cells, i.e. the effect is PER1-dependent. This effect is the same with the (weaker) WIN site inhibitor Inh-4. By contrast, the WBM site inhibitor Inh-6 reduced the amplitude of both wildtype MDFs and *Per1*$^{-/-}$ MDF cells, consistent with the minor role of the WDR5 WBM site for PER1 binding (Fig. 3, this manuscript). Hence, the MDF-*Per1*$^{-/-}$ cells can react to a WDR5 inhibitor if the inhibitor effect is not dependent on PER1. This finding further supports the significance of the lack of response of our *Per1*$^{-/-}$ MDF cells towards the WIN site inhibitors Inh-4/5, which target the clock in a PER1 dependent

manner. The period length of either MDF cells was not significantly affected by any inhibitor.

Reviewer 2: In addition, qPCR data rather point at amplitude enhancing effects of Inh5 treatment (panel c). Further, clear effects remain to be seen in Per1 deficient MDFs (panel d), albeit these are not easily explained.

Differences in the observed phenotypes are due to what we measured. The PER2::Luc reporter (Fig. 6a,b; new Supplementary Fig. 10) is a fusion PROTEIN of the PER2 protein with firefly luciferase. It shows translation, post-translational modifications and half-life of the PER2 protein (i.e. accumulation of the PER2::Luc fusion protein over time), not necessarily its transcriptional regulation. In the qPCR experiments we measured as proxy for transcription the accumulation of *pPer2* and *pBmal1* RNA (and *pPer1* as control). As we only measured qPCR data for 20 h, we cannot determine an amplitude value for the RNA changes but only speak of changes in magnitude (RNA/expression levels).
The *Bmal1::Luc* reporter in U-2 OS cells (new Supplementary Fig. 11a-c) is a transcriptional reporter, and the phenotype observed (increase in magnitude of expression) is what we observed in the qPCR experiments in MDFs on the *Bmal1* pRNA/mRNA level (increase in magnitude of expression/RNA levels rather than change in amplitude; Inh-4: Supplementary Fig. 11d left; Inh-5: Fig. 6c top; Supplementary Fig. 9a,c left). Unfortunately, U-2 OS cells deficient for *Per1* did not produce sufficient bioluminescence to be analyzed. Essentially, experiments with immortalized MDFs yielded the same observation: no rhythmicity in *Per1*$^{-/-}$ MDFs. This incapacity is the reason why we chose to use the MDFs instead, even if these cells were inferior in their oscillations.
We agree with the reviewer that the lack of effects of Inh-5 on *(p)Per2* and *(p)Bmal1* RNA observed in the qPCR experiments with *Per1*$^{-/-}$ MDF cells (Fig. 6d) is not easily explained and could be related to the weaker circadian rhythms of the later passage *Per1*$^{-/-}$ MDF cells used for the qPCR experiments (Supplementary Fig. 10c,d). However, the rescue experiments using *Per1*$^{-/-}$ MDF cells rescued with wildtype PER1 or with the PER1 R111A R1113A WIN site mutant (Fig. 6e,f; Supplementary Fig. 9) validate the significance of the Inh-5 effects in wildtype and *Per1*$^{-/-}$ MDF cells and additionally highlight the importance of the PER1 WIN motif for this PER1-specific effect. Thus, we argue that the lack of Inh-5 effect on *pPer2* and *pBmal1* RNA observed in the *Per1*$^{-/-}$ MDF cells and the *Per1*$^{-/-}$ cells rescued with mutant PER1 is real and not just an artifact resulting from the weaker circadian rhythmicity of late passage *Per1*$^{-/-}$ MDF cells. Moreover, as stated above, the PER2::Luc signal observed in the oscillation experiment (measuring PER::Luc protein abundance) is not equivalent to the *pPer2* and *pBmal1* RNA levels measured in the qPCR experiments.

In the results part of the revised manuscript (p.13-17) we

a) Clarify the different parameters measured in PER2::Luc and *Bmal1*::Luc oscillation experiments and in qPCR experiments (p.13,14,17).
b) Describe the reason for using later passage MDF cells (p.15).
c) Mention that due to the weaker rhythms observed in the late passage *Per1*$^{-/-}$ MDFs it is difficult to state that they don't react to inhibitors Inh-4 and Inh-5 and describe

the limitations of the conclusions that can be drawn only based on the later passage *Per1*$^{-/-}$ cells (p. 15, 17).

d) Describe how our new data (stronger oscillating early passage Inh-5 treated MDF cells and Inh-6 effects) and rescue experiments strengthen our conclusions on the late passage MDF cells, and validate the lack of response of *Per1*$^{-/-}$ MDF cells to the WIN site inhibitors Inh-4,5 (p.15, 17).

e) Emphasize the impact of our PER1 rescue experiments more, as these experiments clearly show that the effects of Inh-5 on *Per2* and *Bmal1* RNA oscillations are dependent on PER1 with a functional WIN motif (Results p. 15 and Discussion p. 21, 22, 24).

Here is a detailed list of the changed parts in the revised manuscript:

Ad a: clarify the different measured parameters (p.13, 14,17).
p. 13:
"To identify a functional role of WDR5 and the PER1/WDR5 complex in the circadian clock, we performed a PER2::Luc bioluminescence reporter assay in mouse dermal fibroblast (MDF) cells in absence and presence of the WDR5 WIN site inhibitor Inh-5. These MDF cells express a PER2-Luciferase fusion protein and therefore the bioluminescence signal reflects the circadian expression, accumulation and degradation of the PER2 protein."
p. 14:
"To further assess the effects of Inh-5 on circadian gene regulation, we performed qPCR experiments targeting the unspliced precursor RNA (p/pre-mRNA) and mature mRNA of *Bmal1*, *Per2* and *Per1* in later (>20) passage MDF cells. While qPCR experiments targeting the unspliced precursor mRNA directly measure transcriptional effects, mature mRNA experiments report transcriptional and post-transcriptional effects. Different from the PER2::Luc oscillation experiment, the qPCR experiments do not report effects related to PER2 protein accumulation and degradation."
p. 17:
"In U-2 OS cells with a *Bmal1*::Luc reporter, which express luciferase from a murine *Bmal1* promotor[1], Inh-4 treatment increased the magnitude of the *Bmal1*::Luc oscillation in a dose dependent manner (Supplementary Fig. 11a) but did not significantly affect the circadian oscillation amplitude or period (Supplementary Fig. 11b,c).
Our qPCR analysis showed that Inh-4 does not affect the *pPer2* pre-mRNA levels but reduces *Per2* mRNA. Additionally, Inh-4 enhances *(p)Bmal1* accumulation, but not as strongly as observed for Inh-5 (Supplementary Fig. 11d, 9a, Fig. 6c). The enhanced *(p)Bmal1* RNA levels in MDF cells are consistent with the increased oscillation magnitude of the *Bmal1*::Luc reporter in Inh-4 treated U-2 OS cells."

Ad b: describe the reason for using later passage MDF cells.
p.15:
"Next, we tested the effect of Inh-5 on *(p)Per2* and *(p)Bmal1* (pre-)mRNA using later (>20) passage wildtype and *Per1*$^{-/-}$ MDF cells that do not overexpress *Wdr5*. Despite their somewhat weaker circadian rhythms (Supplementary Fig. 10c,d) compared to early passage MDF cells (Fig. 6a,b), we used later (>20) passage *Per2*::Luc MDF cells for all our qPCR experiments, because late passage numbers were required to obtain *Per1*$^{-/-}$ rescue

cell lines with wildtype and mutant PER1 by lentiviral infection and selection (see below, Fig. 6e,f; Supplementary Fig. 9c,d)."

Ad c,d: describe the limitations of the conclusions that can be drawn only based on the later passage *Per1*$^{-/-}$ cells, and how our new data (stronger oscillating early passage Inh-5 treated MDF cells, Inh-6 effects) and rescue experiments strengthen our conclusions on the late passage MDF cells treated with Inh-4 or Inh-5.
p.15, Inh-5:
"As observed in early passage *Per2*::Luc MDF cells (Fig. 6a,b), Inh-5 did not affect the period length of the late passage MDF cells, while it reduced the oscillation amplitude of wildtype MDF cells, but apparently not in *Per1*$^{-/-}$ MDF cells (Supplementary Fig. 10 c,d). We note that our conclusions about the lacking effect of Inh-5 on the circadian oscillation amplitude of the later passage *Per1*$^{-/-}$ MDF cells are less reliable due to their weak circadian rhythms, but they are backed up by the statistically more significant observations in the early passage MDF cells (Fig. 6b)."
p.17, Inh-4:
"While our conclusions about Inh-4 are limited by the weak circadian rhythms of the late passage *Per1*$^{-/-}$ MDF cells, the suggested PER1 dependance of the Inh-4 effect on circadian amplitude is consistent with the observed effect of Inh-5 (Fig. 6a,b)."

"In MDF *Per1*$^{-/-}$ cells the effect of Inh-4 on the mature *Per2* mRNA is abolished, whereas *(p)Bmal1* mRNA levels are somewhat reduced, as observed for Inh-5 (Supplementary Fig. 11e). Based on our validation of the equivalent effects of Inh-5 by rescue experiments (Fig. 6c-f; Supplementary Fig. 9) we conclude that the effects of Inh-4 on *Per2* and *(p)Bmal1* mRNA that we observed in wildtype MDF cells also depend on the presence of functional PER1."
p.17, Inh-6:
"The effect of Inh-6 also documents that our *Per1*$^{-/-}$ MDF cells can selectively respond to inhibitors, that affect clock function in a PER1 independent manner. Thereby the Inh-6 experiment further supports the significance of the lacking response of *Per1*$^{-/-}$ cells to the WIN site inhibitors Inh-4 and Inh-5, that affect the circadian clock in a PER1 dependent manner."

Ad e: emphasize the impact of our PER1 rescue experiments more.
Results p.15:
"To confirm that the reduced effects of Inh-5 on *(p)Per2* and *(p)Bmal1* (pre-)mRNA levels observed in our *Per1*$^{-/-}$ MDF cells are not just a consequence of their weaker circadian rhythms, but rather reflect a dependance on functional PER1**,** we performed rescue experiments using later (>20) passage *Per1*$^{-/-}$ MDF cells with genomically integrated V5-tagged PER1 under the control of its native *Per1* promoter. To determine the contribution of the PER1 WIN motif, we tested the rescue ability of wildtype PER1 (referred to as gPER1V5) and of mutant PER1 (referred to as gPER1V5mut) carrying the R1111A R1113A WIN motif mutation, which disrupts the PER1 interaction with WDR5 in ITC experiments (Fig. 3c, Supplementary Fig. 4d) and weakens the WDR5 interaction of full-length PER1 in HEK293 cells (Fig. 5a,b, Supplementary Fig. 7a,b)."
Discussion p. 21:
"Role of WDR5 and PER1/WDR5 interactions in the primary CLOCK/BMAL1/PER/CRY feedback loop

The reduced *Per2* expression in presence of Inh-5 could in principle be due to reduced MLL1-dependant CLOCK/BMAL1 coactivation. However, as the Inh-5 effect on *Per2* (p)mRNA levels is eliminated in *Per1*$^{-/-}$ MDF cells and rescued by wildtype PER1 but not by PER1 with a mutated WIN motif (Fig. 6c-f, Supplementary Fig. 9), it is more likely that Inh-5 reduces *Per2* expression by weakening the WIN site dependent PER1/WDR5 complex formation (Fig. 3f) and thereby enhancing CLOCK/BMAL1 repression."

Discussion p. 22:

"Role of WDR5 and PER1/WDR5 interactions in the secondary BMAL1/REV-ERB/ROR feedback loop

As *Rev-erb* expression is regulated by CLOCK/BMAL1, Inh-5 could in principle enhance *Bmal1* expression by disrupting the co-activating MLL1/WDR5 complex on the *Rev-erb* promotor. However, the Inh-5 effect on *Bmal1* (p)mRNA levels is completely eliminated in *Per1*$^{-/-}$ MDF cells and rescued by wildtype PER1 but not by PER1 with a mutated WIN motif (Fig. 6c-f, Supplementary Fig. 9), suggesting that the effect primarily depends on PER1 with a functional WIN motif."

Discussion p. 24:

"WDR5 WIN site inhibitors mainly affect the circadian clock via the PER1/WDR5 complex

Given that i) Inh-5 treatment results in a much stronger *Bmal1* activation in wildtype MDF cells compared to the *Bmal1* repression observed in *Per1*$^{-/-}$ MDF cells, ii) the negative effect of Inh-5 on *Per2* expression is eliminated in *Per1*$^{-/-}$ MDF cells, and iii) Inh-5 effects that are lost in *Per1*$^{-/-}$ MDF cells can be rescued by wildtype PER1 but not by PER1 with a non-functional WIN site, we conclude that Inh-5 mainly affects the circadian clock by weakening the WIN site mediated PER1/WDR5 complex."

Reviewer 2: Second, while the authors provide various reasons for doing so, I am somewhat concerned with the fact that so many – partially contradictory - data sets have been removed from the revised manuscript. While it is understandable that a consistent story is expected, they might provide important evidence for the interpretation of the results. The reader of the published paper will not have this information which, in the worst case, may misguide his/her understanding of the findings.

Effect of Inh-4 (former C6) on U2OS cells:

For the revised manuscript, we chose to present only the independently replicated experiment from the Kramer laboratory. In these experiments, U-2 OS *Bmal1*::Luc reporter cells exhibited a baseline circadian period of ~25 h, closer to the expected value for this cell line, and treatment with Inh-4 resulted in a dose-dependent increase in reporter magnitude (Supplementary Fig. 11a). Importantly, this effect on magnitude correlates with the increase in *Bmal1* RNA levels observed in rhythmic MDF cells (Supplementary Fig. 11d, left), providing additional support for the biological relevance of the observed response. The original Brown laboratory experiment suggested a dose-*in*dependent period-shortening effect with a longer baseline period (~29 h). Differences in baseline and experimental outcomes are likely attributable to variations in cell clones, passage number, synchronization protocols, medium composition, or other laboratory-specific conditions. Because these differences make a direct comparison difficult, and because the Kramer experiment was independently reproduced under conditions closer to expected baseline behavior and showed a mechanistically interpretable magnitude effect, we decided to include only the Kramer data in the main manuscript. For

transparency, the Brown observations are mentioned here in the Rebuttal, but they are not part of the primary dataset presented in the revised manuscript.

Effect of WDR5 overexpression in U2-OS – and MDF cells

We now see that WDR5 overexpression reduces *Bmal1* RNA in MDF cells (Supplementary data Fig. 8a) which is in line with previous transient transfection experiments (Brown *et al, Science* 2005[2], REF-7). In contrast, the experiment conducted in the Brown laboratory (old Figure 1c) showed that WDR5 overexpression enhances the *Bmal1*::Luc oscillation amplitude in U-2 OS cells. The apparently controversial outcome of these two experiments could be due to different cell lines and reporters (*Bmal1*::Luc reporter in U2-OS cells *vs Bmal1* RNA (qPCR) in our MDF cells), but also due to different WDR5 (over)expression levels. In the U-2 OS cell experiment, the amount of (over)expressed WDR5 was not clearly defined and likely differs from the WDR5 overexpression level used in our new qPCR experiments conducted in MDF cells. With our new qPCR experiments (also suggested by this reviewer) we could quantify WDR5 overexpression on RNA level (Supplementary Fig. 8, right) and show that WDR5 overexpression effects are negated by Inh-5 (Supplementary Fig. 8b), i.e. they are dependent on the WDR5 WIN site. So, we feel that our new experiments resulted in much more interpretable data than the Brown Lab experiment.

Furthermore, the Brown Lab experiment has several uncertainties as also identified by both reviewers: one reviewer raised the concern that only detrended data were shown in old Fig. 1c, from which we cannot conclude that the absolute oscillation amplitude is higher upon WDR5 overexpression. Raw data before detrending could however not be obtained any more as the Brown Lab was closed. Also, dose response curves as suggested by this reviewer could not be obtained as the Brown Lab was closed. Hence, we only show our new results in the revised manuscript.

Co-IP experiments

We decided to remove all Co-IP experiments conducted in the Brown Lab, because both reviewers had very justified doubts about the quantification of the presented PER-WDR5 Co-IPs. One reviewer noted that the background and hence the signal to background ratio cannot be determined with confidence, and the used normalization procedure (normalize on the precipitated WDR5) is conceptually not right. Furthermore, due to the low μM affinity between PER and WDR5, PER-WDR5 Co-IP studies were very inefficient and could only be performed by fixing the cells with formaldehyde. Hence, we cannot conclude anything about the mutant effects on WDR5 affinity from the Co-IP experiments. Co-IPs between wildtype PER1/2 and WDR5 are already published in Brown *et al*, *Science* 2005[2]. As there is nothing to be learned from the Co-IP studies, we decided to remove them entirely from the revised manuscript. We report affinity effects of all PER1/2 mutants based on quantitative ITC studies, so the relevant information is maintained.

Additionally, we show donor saturation Bioluminescence Resonance Energy Transfer (BRET) experiments to prove that the PER1 R1111A R1113A WIN site mutant weakens the WDR5 interaction of the full-length PER1 protein in HEK293 cells (new Fig. 5a,b; Supplementary Fig. 7a,b). The BRET assay is more quantitative than Co-IP experiments, but also a lot of work, so we decided to only characterize the functionally most important

PER1 R1111A R1113A WIN site mutant by BRET and refer to the ITC studies for the other PER mutants.

Due to the technical and conceptual problems associated with all the eliminated studies on Inh-4/C6, WDR5 overexpression and the Co-IPs (see above), we do not think that these data provide important evidence for the interpretation of the results. We rather think that including these data could misguide the reader's understanding of the findings, unless we would clearly describe the problems associated with these old data and down weight them with respect to the more reliable new data. This, in turn, would in our eyes not be a nice behavior towards our former colleague Steve Brown.

Reviewer 2: I understand the technical reasons for not directly testing for the temporal switch in WDR5 interactions. I recommend specifically mentioning this in the discussion and amending the interpretation of the findings accordingly.

We added the following statement in the discussion p. 19:
"Direct analyses of the temporal coordination of endogenous PER/WDR5-, PER/CRY- and PER1/WDR5/CRY complexes in rhythmic cells are technically challenging, as SEC analyses are hampered by the fact that WDR5 forms many multiprotein complexes with similar sizes as PER/CRY containing complexes (Brown *et al, Science* 2005[2]) and Co-IPs of PER1/2 and WDR5 are very inefficient even for overexpressed proteins due to the low μM affinity."

References

1. Brown, S.A., Fleury-Olela, F., Nagoshi, E., Hauser, C., Juge, C., Meier, C.A., Chicheportiche, R., Dayer, J.-M., Albrecht, U., and Schibler, U. (2005). The period length of fibroblast circadian gene expression varies widely among human individuals. PLoS biology *3,* e338. 10.1371/journal.pbio.0030338.
2. Brown, S.A., Ripperger, J., Kadener, S., Fleury-Olela, F., Vilbois, F., Rosbash, M., and Schibler, U. (2005). PERIOD1-associated proteins modulate the negative limb of the mammalian circadian oscillator. Science (New York, N.Y.) *308,* 693–696. 10.1126/science.1107373.
